# Global, regional and national burden of interstitial lung disease and pulmonary sarcoidosis, 1990–2021 and projection to 2040

**DOI:** 10.3389/fmed.2025.1650997

**Published:** 2025-10-27

**Authors:** Xinxin Zhang, Yanting Zhuang, Yizi Xie, Gang Liao, Huiqiu Liang, Wujin Wen, Yuguang Chen, Xiufang Huang, Leshen Lian, Xusheng Qian, Shaofeng Zhan

**Affiliations:** ^1^The First Affiliated Hospital, Guangzhou University of Chinese Medicine, Guangzhou, China; ^2^The First Clinical Medical School, Guangzhou University of Chinese Medicine, Guangzhou, China; ^3^Lingnan Medical Research Centre of Guangzhou University of Chinese Medicine, Guangzhou, China; ^4^Guangdong Provincial Clinical Research Academy of Chinese Medicine, Guangzhou, China; ^5^Shenzhen Hospital of Integrated Traditional Chinese and Western Medicine, Shenzhen, China; ^6^Dongguan Hospital Affiliated to Guangzhou University of Chinese Medicine, Dongguan, China

**Keywords:** interstitial lung disease and pulmonary sarcoidosis, global burden of disease, epidemiology, prediction, trend

## Abstract

**Background:**

Interstitial lung disease and pulmonary sarcoidosis (ILD&PS) represent a group of respiratory diseases characterized by high heterogeneity and substantial burden. In this study, we conducted a comprehensive analysis of burden with ILD&PS and provided estimates for 2040.

**Methods:**

Prevalence, incidence, disability-adjusted life years (DALYs), and deaths were analyzed at global, regional, and national levels using GBD 2021 data. Subgroup analyses were performed by age and gender to assess the quantity of global burden and trends. The BAPC model was used to forecast the worldwide disease load until 2040.

**Results:**

From 1990 to 2021, global prevalence, incidence, DALYs, and mortality rates of ILD&PS increased by 128% (1887445.26 to 4306627.72), 148% (157,441.17 to 390,267.11), 169% (1,501,028.43 to 4,042,150.49), and 242% (54,967.23 to 188,222.37), respectively. Furthermore, the corresponding age-standardized rates (ASRs) also showed an upward trend. Additionally, the burden in Australasia and Andean Latin America varied greatly at the regional level, with ASRs burden increasing highest in high sociodemographic index (SDI) region over the previous 32 years. Correlation analysis revealed a positive correlation between ASR burden and SDI. Subgroup analysis showed a higher burden in adults over 50 and consistently greater burden in males than females.

**Conclusion:**

The overall burden of ILD&PS increased from 1990 to 2021, and by 2040, the ASRs burden were expected to progressively normalize. Policymakers should give prevention and treatment measures top priority, paying special attention to high-burden areas and populations.

## 1 Introduction

Interstitial lung disease and pulmonary sarcoidosis (ILD&PS) are major respiratory health issues that affect people all over the world. They are defined by chronic conditions that affect lung function and oxygen exchange due to scarring or inflammation (1, 2). Based on the Global Burden of Disease (GBD) 2019 study, an estimated 4.71 million people worldwide are afflicted by ILD&PS in 2019, resulting in negative health and economic impacts (3).

The rare idiopathic pulmonary fibrosis (IPF) is one of the many ailments that fall under the umbrella of interstitial lung diseases (ILDs), and there are differences in the ways that these conditions are treated. IPF patients usually experience a relentless progression of their illness (4). Regardless of their distinct diagnostic labels, a notable percentage of other types of ILDs, between 15 and 40%, are expected to develop pulmonary fibrosis (5). In addition, connective tissue disease-associated ILD is also a subtype of ILDs (6). There is a certain association between ILDs and pulmonary sarcoidosis (PS), especially when it comes to smoking history and pathological characteristics (7). Even though the inflammatory infiltrate of PS may eventually go away, persistent disease activity can result in pulmonary fibrosis (8). Furthermore, over 10% of individuals with PS are likely to experience a progressive disease (9). It is well-known treatment expenditures for IPF patients increase significantly over the course of 36 months, with per capita drug prices growing from €1,442 to €11,000 in Germany (10). In the context of ongoing research efforts into the detection, diagnosis, and therapeutic management of ILD&PS, the substantial burden of these conditions continues to highlight the need for heightened attention and further research.

The GBD study is a timely assessment of key health outcomes that currently covers thousands of diseases, injuries and risk factors in more than 200 countries and 20 territories, giving us a unique perspective on the overall picture of disease and trends (11, 12). Based on data from GBD 2021, this study analyzed the trends in health burden of ILD&PS at the global, regional, and national levels between 1990 and 2021 by stratifying the data by age and gender. Prior research on GBD has largely focused on the disability-adjusted life year (DALYs) of the disease and regional levels of SDI (13). The analysis will be expanded in this study to include age-standardized prevalence, incidence, DALYs and deaths. Additionally, this study also evaluated and projected the disease burden through 2040 to help guide the development of prevention and control strategies and to offer more insights into disease trends.

## 2 Materials and methods

### 2.1 Data source

The GBD 2021 collects information from a number of nations and areas, including census data, health service contact data, and other reports and registered data. The basic characteristics of 371 illnesses and injuries are captured in this dataset, which spans 204 countries and territories and 811 subnational regions (14). Using GBD2021 (accessed on 27 October 2024),^1^ our study extracted pertinent data on ILD&PS (Code: D86, J84) at the global, regional and national levels from 1990 to 2021. The exact ICD-10 classification codes can be accessed via the Institute for Health Metrics and Evaluation’s (IHME) official online platform.^2^ As a multifaceted indicator, DALYs quantified the impact of diseases, injuries, and risk factors on an individual’s health by accounting for both Years Lived with Disability (YLD) and Years of Life Lost (YLL) (15). We focused on the prevalence, incidence, DALYs and deaths for different age groups and genders in the ILD&PS data. In more detail, we included data on the number and rate for the following age groups: (0–14 years, 15–19 years, 20–24 years, 25–29 years, 30–34 years, 35–39 years, 40–44 years, 45–49 years, 50–54 years, 55–59 years, 60–64 years, 65–69 years, 70–74 years, 75–79 years, 80–84 years, 85–89 years, 90–94 years and 95 + years), gender (female and male) and regions for analysis. 21 GBD regions, such as Eastern Europe, Central Asia, and High-income Asia Pacific, were included in the GBD geographical framework (16). Furthermore, to represent the social and economic standing of various regions, the 204 countries worldwide were categorized into five quintiles based on SDI. The SDI regions, which were ranked from 0 to 1 (low, low-middle, middle, high-middle, and high), were in line with the degree of economic development (17).

### 2.2 Statistics analysis

The results of the trends over time were more reliable since age-standardized rates (ASRs) were used to account for differences in age structure among various populations (18). Percentage change (PC) and estimated annual percentage change (EAPC) were calculated using both ASRs per 100,000 and cases. PC was calculated using the following precise formula based on the results obtained from the GBD study for the years 1990 and 2021: (Value in 2021−Value in 1990)/1990. Furthermore, we applied a log-linear regression model, transforming the measured value y with a logarithmic conversion. The model was constructed as follows: *y = α + βx + ϵ*, where year was the variable *x*, *α* was the intercept, *β* represented the annual change rate, and *ϵ* was the error term. Then, using the regression model, the EAPC and its 95% confidence interval (CI) were determined to be 100 × (exp(*β*) − 1) (19). The 95% of the uncertainty interval (UI) was generated from 1,000 random selections of the 2.5th and 97.5th percentile values (20). Spearman rank test was used to assess the correlation between burden indicators (prevalence, incidence, DALYs, deaths) and SDI in different regions (21).

The worldwide burden of ILD&PS was predicted until 2040 using the Bayesian age-period-cohort (BAPC) model. The BAPC model was a statistical model that considered the effects of age, period, and cohort. It merged Bayesian statistical methods with integrated nested Laplace approximation (INLA) technology (3). The birth year of each cohort was calculated as the calendar year minus the central age within each 5-year age group. Our study captured cohorts born between 1895 (oldest participants in 1990) and 2025 (youngest projected group in 2040), while the core observational data encompassed the 1935–2006 birth years (median: 1970).

Data analysis and visualization were generated in R (version 4.3.1), and the R packages “tidyverse”, “ggplot2”, “map”, “sf”, “ggsci”, “segmented”, “INLA”, “BAPC”, and others were used.

## 3 Results

### 3.1 Global burden

Globally, the number of prevalent cases rose by 128% from 1990 to 2021, from 1887445.26 (95% UI, 1,609,368.79 to 2,206,969.22) to 4306627.72 (95% UI, 3,802,950.84 to 4,898,714.45). The age-standardized prevalence rate (ASPR) was 45.99 (95% UI, 39.42 to 53.78) in 1990 and 50.01 (95% UI, 44.24 to 56.77) per 100,000 in 2021 (Table 1; Supplementary Table S1). Besides, the number of incidence cases caused by ILD&PS increased from 157,441.17 (95% UI, 136,251.29 to 179,471.82) in 1990 to 390,267.11 (95% UI, 346,393.42 to 433,403.27) in 2021, a considerable increase of 148%. The age-standardized incidence rate (ASIR) was recorded at 3.77 (95% UI, 3.27 to 4.28) per 100,000 population in 1990 and soared to 4.54 (95% UI, 4.05 to 5.04) in 2021 during this period (Supplementary Tables S1, S2). The number of DALYs grew by 169% between 1990 and 2021, with the case rising from 1,501,028.43 (95% UI, 1,221,196.88 to 1,850,556.94) to 4,042,150.49 (95% UI, 3,489,794.64 to 4,516,882.92; Supplementary Tables S1, S3). The age-standardized DALY rate (ASDR) increased from 37.15 (95% UI, 30.62 to 45.37) in 1990 to 47.62 (95% UI, 41.26 to 53.16) in 2021 per 100,000 people. Furthermore, there was a 242% increase in the number of deaths from 1990 to 2021, from 54,967.23 (95% UI, 44,761.39 to 68,391.19) to 188,222.37 (95% UI, 161,405.66 to 212,251.52) in 2021. Additionally, the age-standardized mortality rate (ASMR) was 1.52 (95% UI, 1.25 to 1.87) in 1990 and 2.28 (95% UI, 1.96 to 2.56) in 2021 (Supplementary Tables S1, S4). Meanwhile, there was a notable rising trend worldwide in the EAPC for ASPR, ASIR, ASDR and ASMR. The EAPC for ASPR, ASIR, ASDR and ASMR per 100,000 population was specifically 0.36 (95% UI, 0.28 to 0.45), 0.72 (95% UI, 0.63 to 0.82), 0.95 (95% UI, 0.85 to 1.05) and 1.55 (95% UI, 1.41 to 1.70) for 1990–2021, respectively (Supplementary Tables S1–S4).

**Table 1:** Number and age-standardized prevalence of ILD&PS, 1990 vs. 2021 (Global, SDI Quintiles and GBD region).

Location	Number in 1990 (95% UI)	ASPR in 1990 (per 100,000, 95% UI)	Number in 2021 (95% UI)	ASPR in 2021 (per 100,000, 95% UI)	EAPC of ASPR (95% CI)
Global	1,887,445.26(1,609,368.79–2,206,969.22)	45.99(39.42–53.78)	4,306,627.72(3,802,950.84–4,898,714.45)	50.01(44.24–56.77)	0.36(0.29–0.44)
SDI quintile					
Low SDI	64,790.26(54,155.17–76,331.04)	25.68(21.67–30.09)	152,795.77(131,758.78–177,190.27)	26.57(23.17–30.40)	0.16(0.12–0.21)
Low–middle SDI	236,665.38(198,226.58–278344.47)	35.77(30.26–41.93)	600,252.87(524,699.56–690,438.27)	39.70(34.81–45.31)	0.43(0.38–0.47)
Middle SDI	324,844.49(271,302.89–385,904.55)	28.48(24.08–33.82)	902,157.69(787,325.52–1,037,654.94)	33.03(28.83–37.97)	0.64(0.56–0.73)
High–middle SDI	351,805.94(300,237.53–413,791.59)	34.10(29.26–39.96)	704,695.64(626,654.48–800,962.81)	36.39(32.23–41.24)	0.38(0.27–0.49)
High SDI	907,883.91(783,539.02–1,058,221.31)	84.18(72.93–97.79)	1,944,285.73(1,717,676.01–2,193,796.91)	98.58(87.80–111.30)	0.55(0.46–0.63)
GBD region					
Andean Latin America	15,934.55(14,531.35–17,510.08)	76.60(69.74–84.04)	79,686.64(73,987.56–85,343.50)	135.98(126.12–145.58)	2.37(2.21–2.52)
Australasia	8,621.66(7,526.31–9,869.87)	36.58(31.86–42.01)	32,955.28(29,674.83–36,524.70)	61.80(55.39–68.95)	1.77(1.58–1.95)
Caribbean	4,238.12(3,555.30–4,976.85)	15.36(13.00–18.01)	11,197.79(9,896.85–12,639.02)	21.09(18.65–23.82)	1.16(1.07–1.26)
Central Asia	17,284.03(15,188.42–19,815.22)	34.69(30.71–39.36)	31,350.37(27,947.89–35,422.05)	35.86(32.32–40.28)	0.14(−0.1–0.38)
Central Europe	56,133.54(48,055.34–65,348.86)	38.21(32.69–44.77)	70,262.47(62,022.23–79,643.06)	38.39(33.44–44.05)	0.24(0.15–0.32)
Central Latin America	35,145.83(29,813.37–40966.97)	39.05(33.59–45.27)	116,100.80(103,368.79–130,258.37)	45.91(41.07–51.56)	0.45(0.41–0.49)
Central Sub-Saharan Africa	4,361.76(3,559.19–5,232.33)	16.45(13.78–19.46)	12,477.51(10,450.99–14,821.53)	18.30(15.77–21.18)	0.42(0.29–0.54)
East Asia	260,268.45(213,538.74–316,111.96)	27.06(22.44–32.71)	647,955.24(552,500.17–759,163.33)	29.30(25.22–34.20)	0.58(0.4–0.75)
Eastern Europe	90,878.88(75,716.69–10,7683.28)	33.43(27.72–39.85)	52,794.09(43,745.32–62,988.74)	17.88(14.66–21.42)	−2.28(−2.36–−2.21)
Eastern Sub-Saharan Africa	11,900.92(9,599.75–14,376.06)	13.24(10.93–15.74)	30,904.05(25,480.24–36,978.12)	14.57(12.34–17.07)	0.31(0.29–0.33)
High-income Asia Pacific	274,572.22(234,702.35–319,642.00)	134.65(115.30–156.65)	642,118.40(564,109.76–731,621.74)	151.60(134.19–172.06)	0.43(0.3–0.56)
High-income North America	393,390.79(338,217.63–460,044.31)	116.11(100.03–135.71)	787,778.92(695,019.71–893,373.99)	127.50(113.42–143.85)	0.19(0.13–0.26)
North Africa and Middle East	48,260.51(40,001.29–57,469.52)	24.62(20.90–28.99)	185,663.20(160,882.75–214498.06)	35.72(31.40–40.69)	1.38(1.29–1.47)
Oceania	1,717.74(1,500.73–1962.74)	42.55(37.75–48.11)	4,879.76(4,390.83–5410.87)	49.16(44.66–54.30)	0.39(0.34–0.44)
South Asia	293,637.91(24,6481.45–34,5905.57)	47.25(39.91–55.76)	791,408.05(688,352.14–911271.94)	51.07(44.55–58.79)	0.32(0.27–0.38)
Southeast Asia	37,165.64(30,049.99–45,335.01)	13.22(10.95–15.79)	120,933.17(103,686.65–141,729.09)	17.40(15.03–20.22)	0.9(0.88–0.91)
Southern Latin America	27842.24(25030.60–30972.49)	59.53(53.60–66.25)	85,609.49(78,824.93–92,601.20)	99.07(91.46–107.24)	1.67(1.57–1.78)
outhern Sub-Saharan Africa	13,391.60(11,191.11–15,781.66)	45.27(38.30–53.16)	25,862.77(22,187.02–30,076.82)	41.18(35.60–47.56)	−0.42(−0.57–−0.28)
Tropical Latin America	28,813.11(23,850.30–34,561.91)	27.45(22.97–32.54)	52,675.16(45,618.33–60,595.19)	20.45(17.76–23.47)	−1.12(−1.28–−0.96)
Western Europe	249,467.13(219,645.03–284,530.89)	46.08(40.10–53.29)	491,892.59(44,1706.92–547,311.03)	58.14(52.00–65.11)	0.98(0.79–1.16)
Western Sub-Saharan Africa	14,418.65(11,653.98–17,390.25)	14.07(11.53–16.78)	321,21.97(26,136.06–38,830.43)	12.79(10.70–15.15)	−0.29(−0.35–−0.23)

ILD&PS, Interstitial lung disease and pulmonary sarcoidosis; SDI, Socio - demographic index; ASIR, Age - standardized incidence rate; EAPC, Estimated annual percentage change; UI, Uncertainty interval; CI, Confidence interval.

### 3.2 SDI region burden

**Figure 1:**
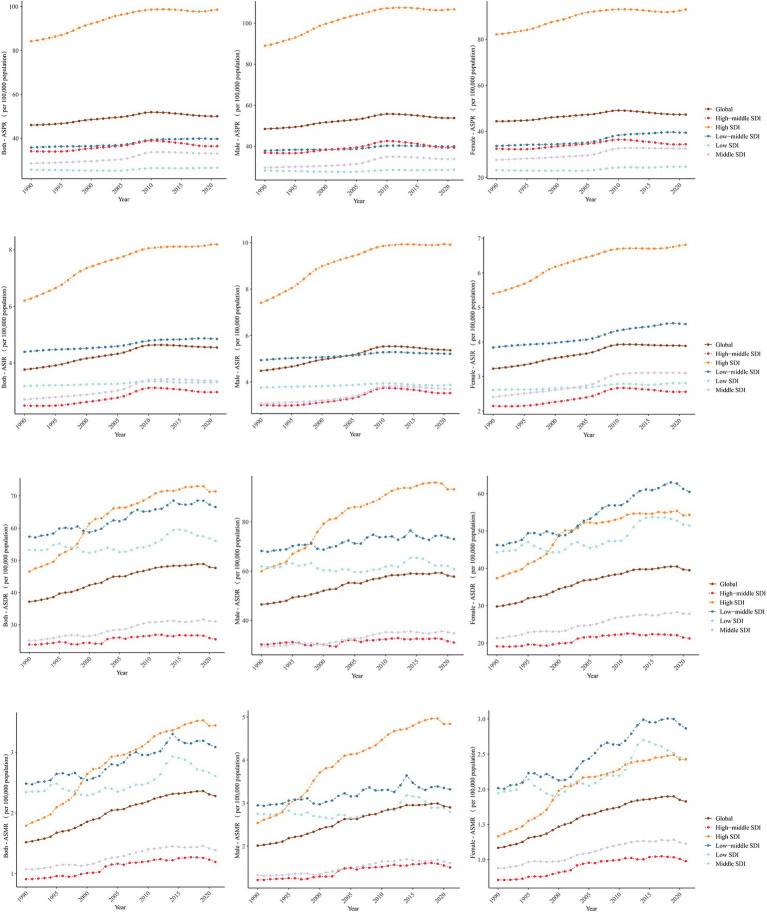
Age-standardized rates of ILD&PS by global and SDI regions, 1990 to 2021. ILD&PS, Interstitial lung disease and pulmonary sarcoidosis; SDI, socio-demographic index; ASPR, Age-standardized prevalence rate; ASIR, Age-standardized incidence rate; ASDR, Age-standardized DALYs rate; ASMR, Age-standardized mortality rate.

The Middle SDI region showed the biggest percentage growth between 1990 and 2021, with absolute prevalence, incidence, and deaths rates rising 1.78, 1.93, and 2.01 times, respectively. However, the fastest rise in PC mortality cases (3.07 times) was observed in the high SDI region, from 20,063.97 (95% UI, 18,621.72 to 20,871.04) in 1990 to 81,732.30 (95% UI, 71,243.83 to 88,091.88) in 2021 (Supplementary Tables S1–S4). From 1990 to 2021, the high SDI region exhibited the most significant increasing percentage change trend across all four parameters when taking ASRs into account. The high SDI region stood out with increases of 17% in prevalence (EAPC = 0.55), 32% in incidence (EAPC = 0.92), 53% in DALYs (EAPC = 1.54) and 92% in mortality (EAPC = 2.3). In all five SDI regions, ASPR, ASIR, ASDR, and ASMR generally displayed an ascending trend over the last 32 years (Table 1; Figure 1; Supplementary Tables S1–S4).

### 3.3 GBD region burden

Between 1990 and 2021, there was an estimated positive rise in ILD&PS prevalence cases in 95.2% of the locations (Supplementary Figure S1). The three regions with the biggest shifts in prevalence were Andean Latin America, North Africa and Middle East, and Australasia (4.00, 2.85, and 2.82 times respectively). The absolute cases in 1990 were 15,934.55 (95% UI, 14,531.35 to 17,510.08), 48,260.51 (95% UI, 40,001.29 to 57,469.52) and 8,621.66 (95% UI, 7,526.31 to 9,869.87), respectively. In 2021, however, they climbed to 79,686.64 (95% UI, 73,987.56 to 85,343.50), 185,663.20 (95% UI, 160,882.75 to 214,498.06) and 32,955.28 (95% UI, 29,674.83 to 36,524.70), respectively (Table 1; Supplementary Tables S1, S2). Australasia, on the other hand, was second only to Andean Latin America in terms of incident cases and had the greatest variation in DALYs cases (4.79 times) and deaths (6.23 times; Supplementary Table S1). At the regional level in 2021, High-income Asia Pacific had the highest ASPR (151.60, [95% UI, 134.19 to 172.06] per 100,000 population), while Andean Latin America had the highest ASIR, ASDR and ASMR (20.47, 209.34 and 11.37 per 100,000 population, respectively). In contrast, the Western Sub-Saharan Africa showed the lowest ASPR (12.79 [95%UI, 10.70 to 15.15]) and Eastern Europe experienced the lowest ASIR (1.04 [95%UI, 0.89 to 1.21]) in 2021. Southeast Asia also showed far lower ASDR (8.93 [95%UI, 5.18 to 16.51]) and ASMRs (0.33 [95%UI, 0.17 to 0.66]; (Table 1; Supplementary Tables S2–S4).

### 3.4 National burden

**Figure 2:**
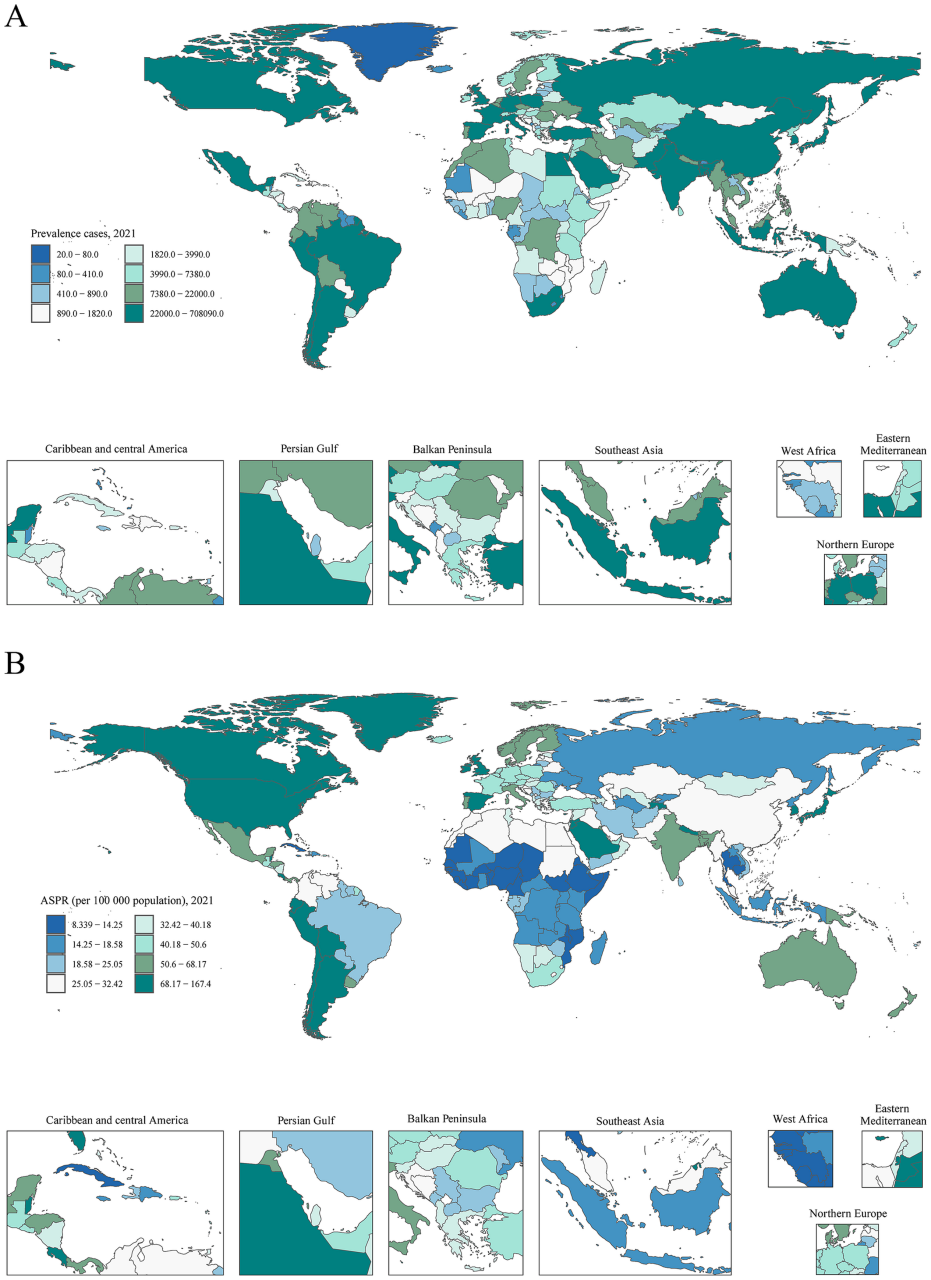
Prevalence and age-standardized rates of ILD&PS in 204 countries and territories. **(A)** Prevalence cases in 2021; **(B)** ASPR per 100,000 population in 2021. ILD&PS, Interstitial lung disease and pulmonary sarcoidosis; ASPR, Age-standardized prevalence rate.

From 1990 to 2021, over 90% of countries experienced an upward trend in the cases prevalence, incidence, DALYs and mortality rates of ILD&PS (Supplementary Figure S1; Supplementary Table S5). In 2021, United States of America had the highest prevalence and incidence among 204 countries (708,091.63 [95% UI, 621,857.11 to 808,616.38] and 59,754.49 [95% UI, 52,045.56 to 67,737.27]), followed by India (654,924.60 [95% UI, 565,718.36 to 757,500.39] and 81,114.27 [95% UI, 70,536.27 to 91,985.16]), and China (628,382.72 [95% UI, 534,993.12 to 737,822.25] and 48,513.74 [95% UI, 41,541.45 to 55,949.02]; (Figure 2; Supplementary Figure S2; Supplementary Tables S6, S7). Besides, it was estimated that India, United States of America, and Japan exhibited the highest absolute numbers of DALYs (1,124,247.84 [95%UI, 750,835.18 to 1,523,498.82], 524,808.48 [95%UI, 478,755.29-560,666.59] and 383,903.14 [95% UI, 335,660.18 to 419,247.70]) and deaths (47,336.08 [95%UI, 30,672.40 to 65,803.94], 26,601.58 [95% UI, 23,244.93 to 28,216.49] and 24,025.65 [95% UI, 19,890.74 to 26,344.87]; (Supplementary Figures S3, S4; Supplementary Tables S8, S9). In addition, Peru had the greatest rates of ASPR, ASIR, ASDR, and ASMR resulting from ILD&PS at 167.38 (95% UI, 155.61 to 179.28), 24.73 (95% UI, 23.23 to 26.22), 246.21 (95% UI, 178.27 to 317.79) and 13.31 (95% UI, 9.20 to 17.55) per 100,000 populations, respectively. Conversely, Ukraine’s ASPR (−66%) and ASIR (−67%) showed declining trends, with EAPCs of −4 (95% CI, −17.73 to 12.03) and −4.15 (95% CI, −19.51 to 14.15), respectively. Latvia was found to have the most notable decline in ASDR (−89%), while the Republic of Moldova showed the largest loss at −93% in ASMR (Supplementary Figures S2–S6; Supplementary Tables S5–S9).

### 3.5 Association of burden with SDI

**Figure 3:**
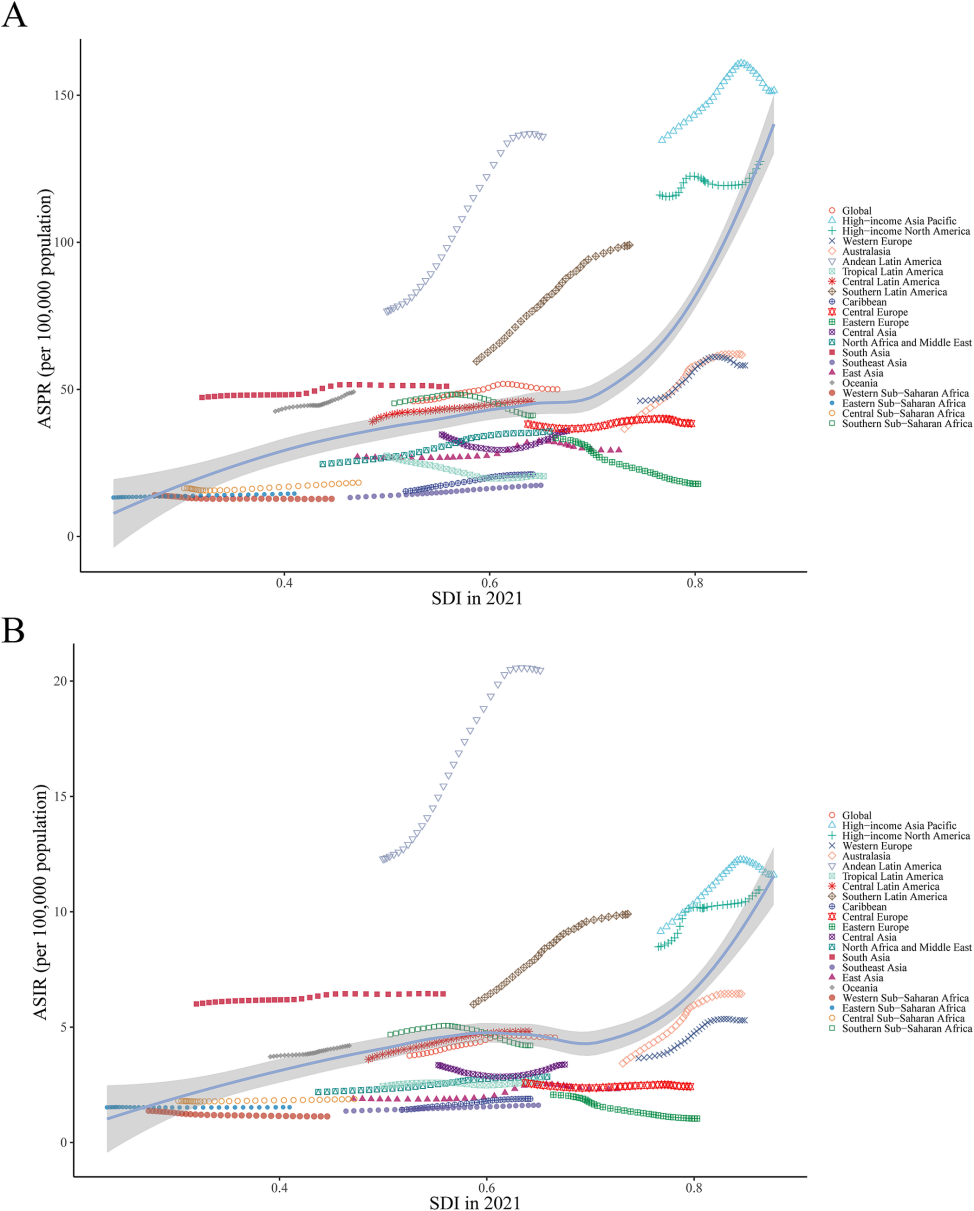
ASPR and ASIR of ILD&PS by SDI in 2021, globally and in 21 regions, 1990 to 2021. **(A)** ASPR across 21 regions according to SDI in 2021; **(B)** ASIR across 21 regions according to SDI in 2021. ASPR, Age-standardized prevalence rate; ASIR, Age-standardized incidence rate; ILD&PS, Interstitial lung disease and pulmonary sarcoidosis; SDI, Socio-demographic index.

ASPR (*p* < 0.001, *r* = 0.57), ASIR (*p* < 0.001, *r* = 0.44), ASDR (*p* < 0.001, *r* = 0.17) and ASMR (*p* < 0.001, *r* = 0.19) were found to positively correlate with SDI at the regional level. South Asia, Oceania, Andean Latin America, and Southern Latin America all had burden values in 2021 that were higher than anticipated for their respective SDI levels. On the contrary, during the measurement period, burden levels were lower than expected for each of the following regions: Western Europe, Tropical Latin America, the Caribbean, Central Europe, Eastern Europe, Central Asia, North Africa, Middle East, Southeast Asia and East Asia (Figure 3; Supplementary Figure S7).

**Figure 4:**
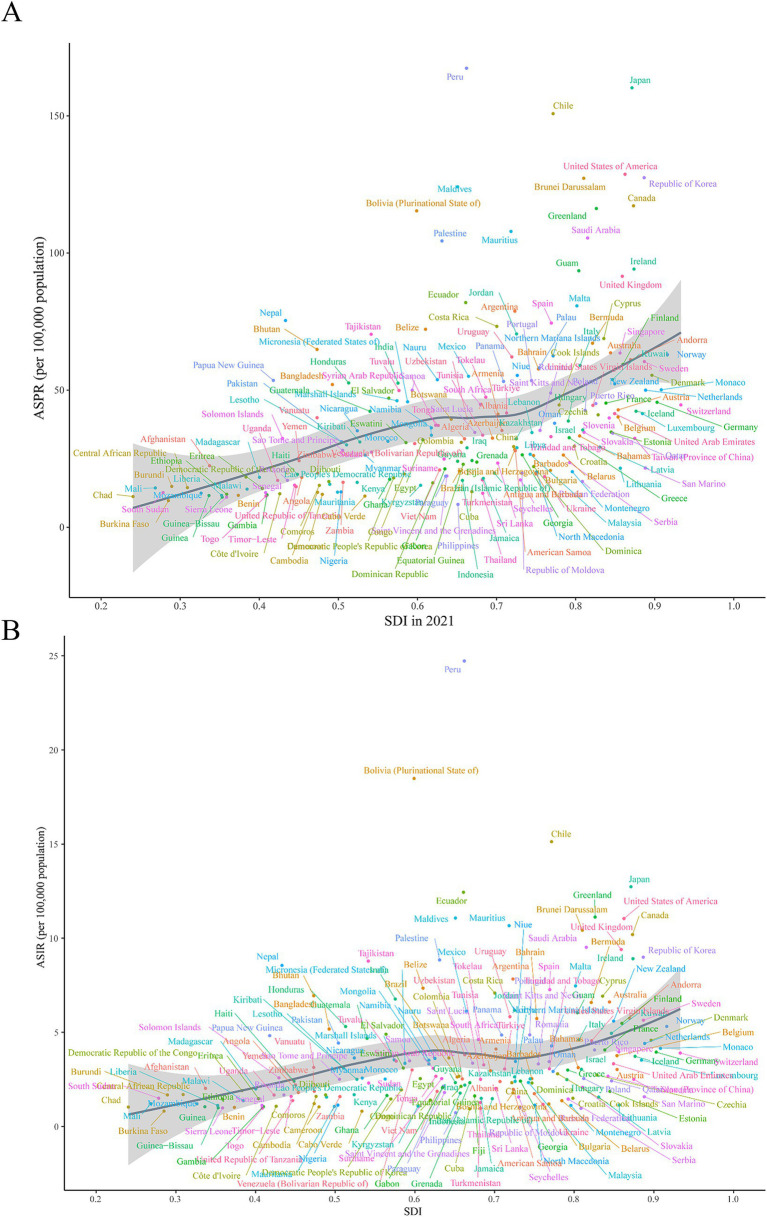
ASPR and ASIR of ILD&PS by SDI in 2021 in 204 countries and territories, 1990 vs. 2021. **(A)** ASPR in 204 countries and territories; **(B)** ASIR in 204 countries and territories. ASPR, Age-standardized prevalence rate; ASIR, Age-standardized incidence rate; ILD&PS, Interstitial lung disease and pulmonary sarcoidosis; SDI, socio-demographic index.

**Figure 5:**
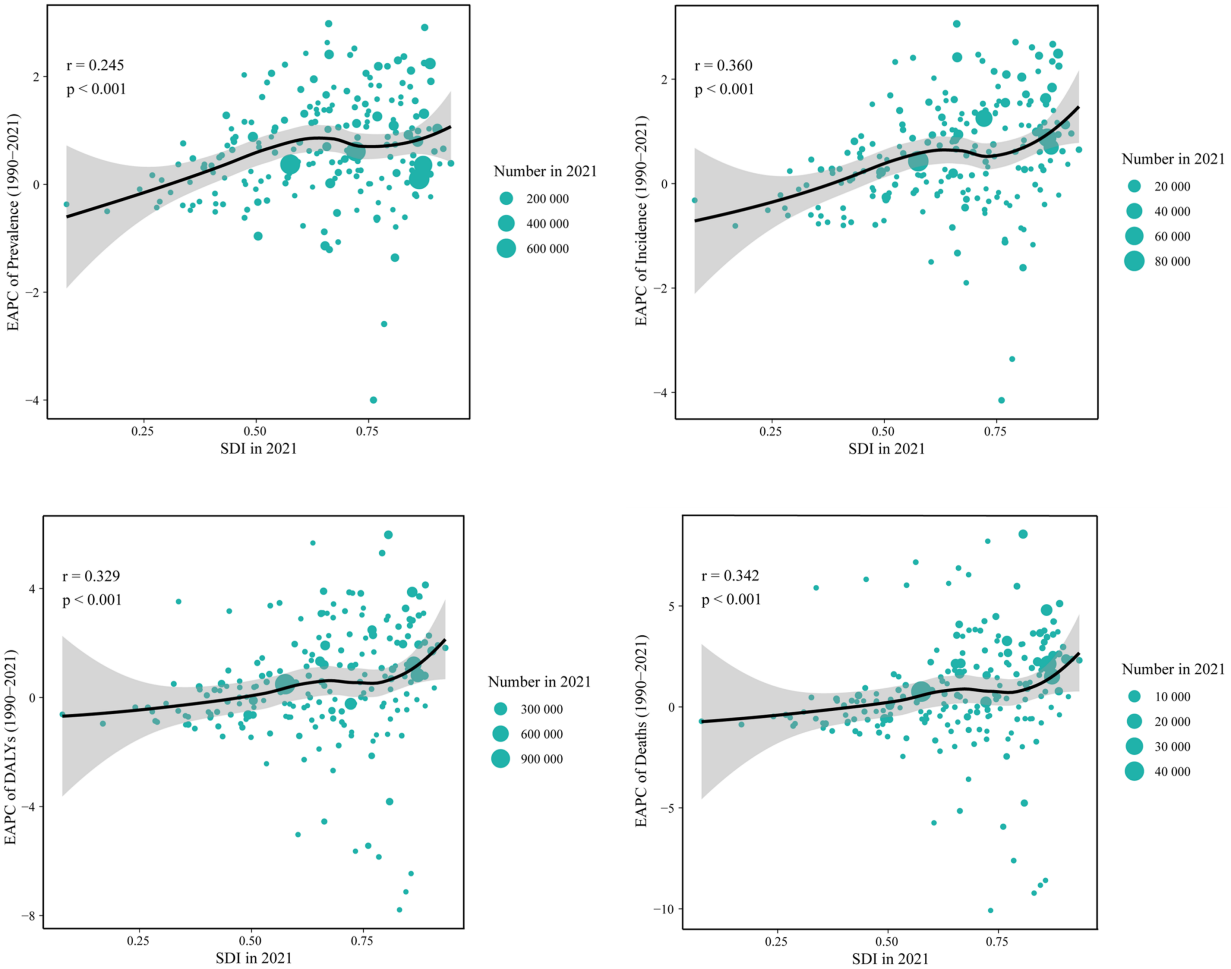
Correlation between the SDI and EAPC of ILD&PS burden, by number for 204 contries in 2021. SDI, socio-demographic index; EAPC, Estimated annual percentage change; ILD&PS, Interstitial lung disease and pulmonary sarcoidosis; DALYs, Disability adjusted life years.

At the national level, there was a positive correlation between burden metrics and SDI levels in 2021, with SDI levels falling between 0.2 and 0.6 and between 0.8 and 1.0. Similarly, it was shown that Ecuador, Chile, Bolivia, and Peru significantly exceeded the burden indices that SDI had predicted (Figure 4; Supplementary Figure S8). Furthermore, substantial differences (*p* < 0.001) were found between the age-standardized burden of EAPC and SDI, with a weak to moderate connection between the two. Both in terms of numbers and age-standardized rates, a larger burden of ILD&PS was associated with higher SDI. The correlation coefficients of ASPR, ASIR, ASDR, and ASMR with SDI and EAPC were 0.25, 0.36, 0.33, and 0.34, respectively (Figure 5; Supplementary Figure S9).

### 3.6 Age and gender patterns

**Figure 6:**
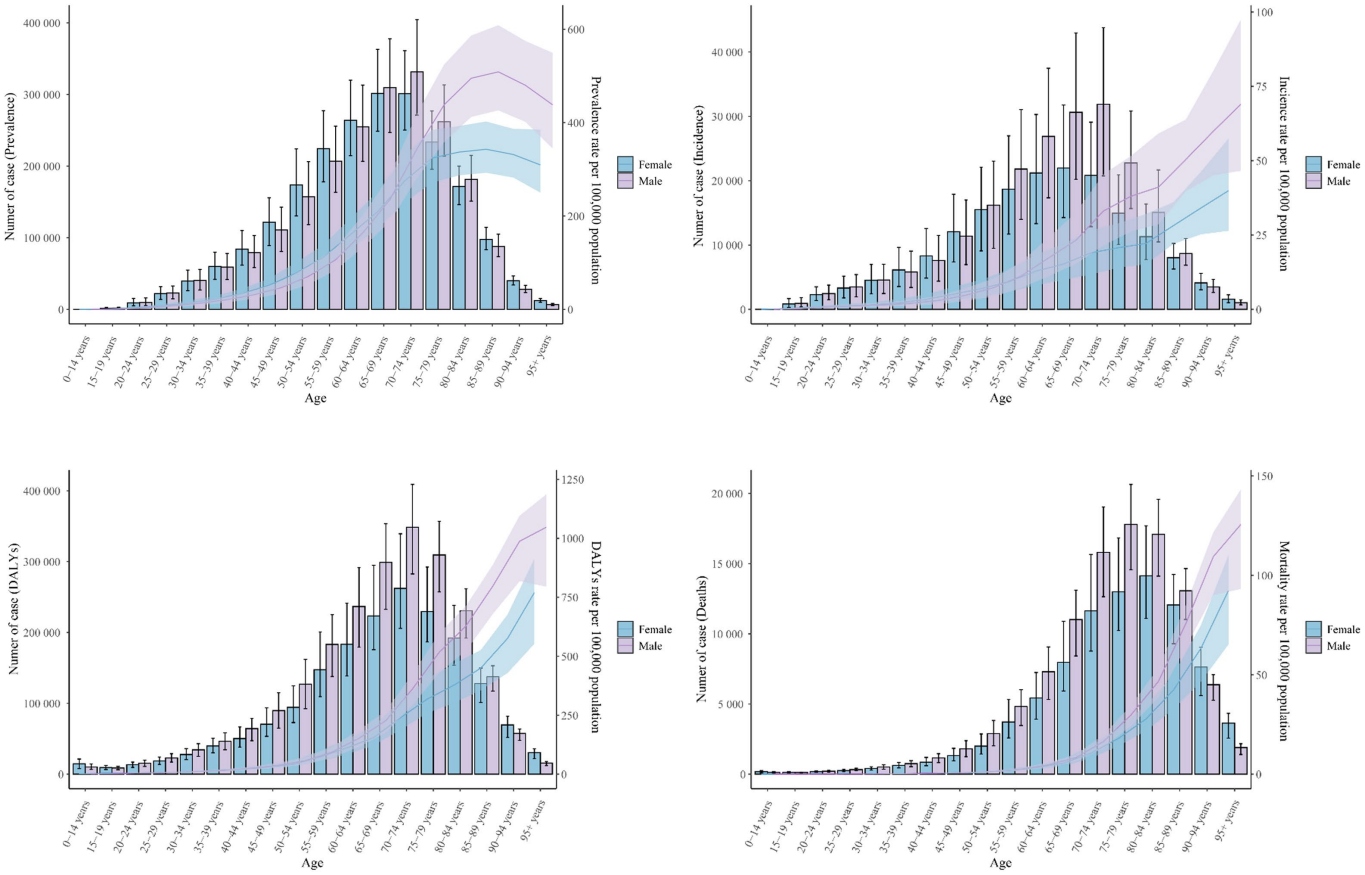
Trends in the cases and age-standardized rates for ILD&PS by male and female, 2021. ILD&PS, Interstitial lung disease and pulmonary sarcoidosis; DALYs, Disability adjusted life years.

**Figure 7:**
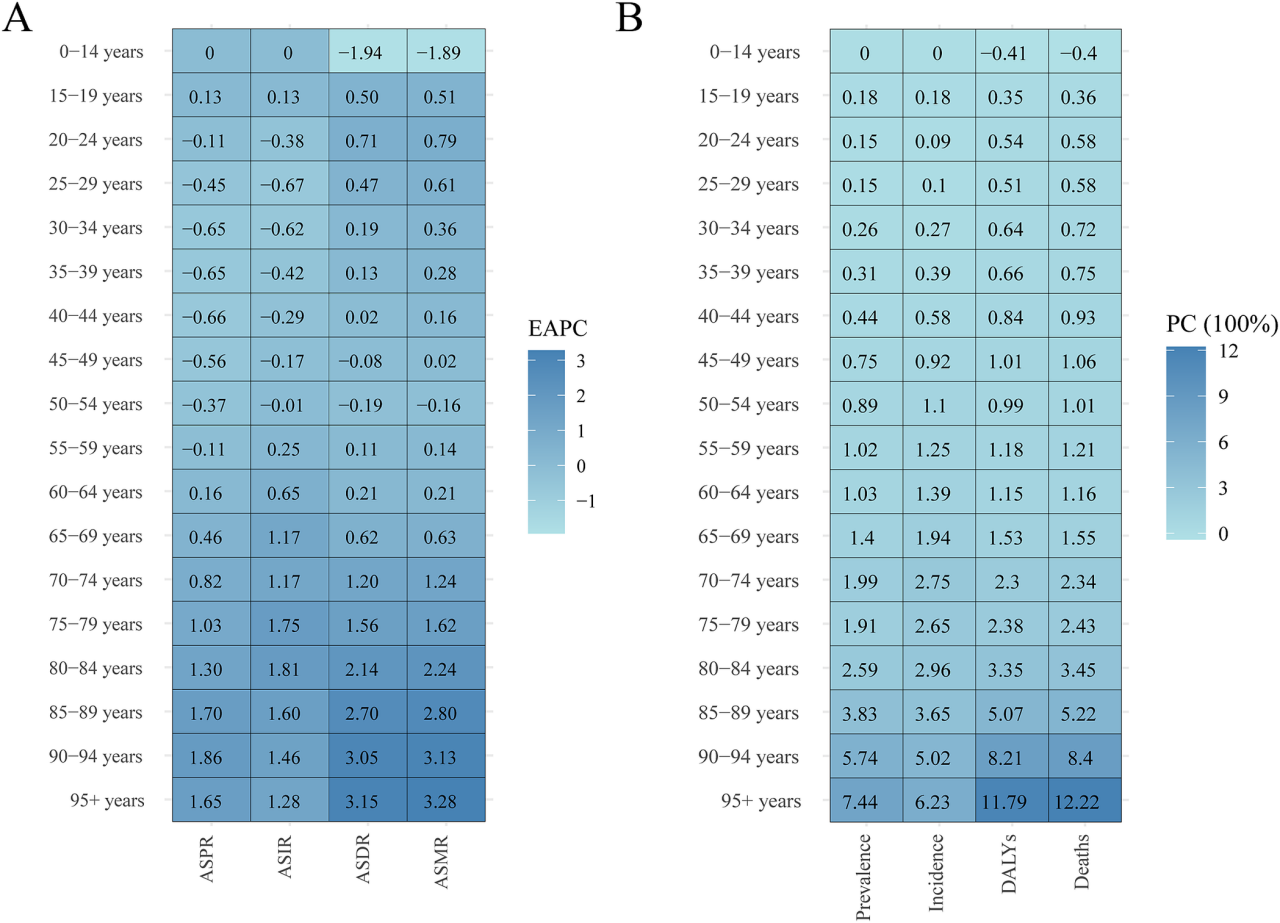
Heatmap of ILD&PS burden by age groups 1990 vs. 2021. **(A)** The EPCA heatmap across age groups; **(B)** The PC heatmap across age groups. ILD&PS, Interstitial lung disease and pulmonary sarcoidosis; EAPC, Estimated annual percentage change; PC, Percentage change; ASPR, Age-standardized prevalence rate; ASIR, Age-standardized incidence rate; ASDR, Age-standardized DALYs rate; ASMR, Age-standardized mortality rate; DALYs, Disability adjusted life years.

Globally, the 50–89 age group accounted for the majority of prevalence, incidence, DALYs, and fatalities in 2021. The 70–74 age group had the highest prevalence rates (632,825.47 [95% UI, 520,341.87-762,125.37]), incidence rates (52,707.37 [95% UI, 33,629.15 to 72,822.83]), and number of DALYs (610,775.30 [95% UI, 514,813.62 to 715,439.22]) in 2021, while the 80–84 age group had the highest number of deaths (31,227.21 [95% UI, 26,079.54 to 36,046.41]). The highest PC occurred in the 95 + age group between 1990 and 2021, with increases of 35%, 139%, and 147%, respectively. It is interesting to note that the gains in ASIR, ASDR, and ASMR were in line with age growth. However, ASPR peaked between the ages of 85 and 89, after which it started to progressively fall. The 90–94 age group experienced the fastest growth in prevalence and DALYs over the course of 32-year period, with an EAPC of 1.86 (95% CI, 1.64 to 2.08) and 3.05 (95% CI, 2.79 to 3.32), respectively. Furthermore, the age group of 80–84 years old (EAPC = 1.81) had the largest increase in incidence rates, whereas the age group of 25–29 years old (EAPC = −0.67) had the largest reduction. The trend analysis of global mortality rates from 1990 to 2021 indicated that the mortality rates for the 0–14 and 50–54 age groups were on a declining trend, with EAPCs of −1.89 (95% CI, −2.07 to −1.71) and −0.16 (95% UI, −0.25 to −0.07), respectively (Figures 6, 7; Supplementary Tables S10–S13).

The age-standardized disease burden for males was greater than that for females, indicating a sex difference in 2021. In 2021, prevalence, incidence, DALYs, and mortality were as follows for the male population: 2,149,200.96 (95% UI, 1,902,460.13 to 2,433,401.43), 214,681.18 (95% UI, 190,533.20 to 238,498.19), 2,237,269.37 (95% UI, 1,839,499.94 to 2,555,199.73), and 103,056.70 (95% UI, 84,156.40 to 115,833.40). However, compared to the male population, the female population experienced more noticeable variations in ASDR and ASMR between 1990 and 2021. In particular, women’s EAPC for ASDR was 1.07 (95% CI, 0.96 to 1.17), higher than men’s, which was 0.85 (95% CI, 0.76 to 0.94). With an EAPC for ASMR rates of 1.7 (95% CI, 1.54 to 1.86) for females and 1.41 (95% CI, 1.28 to 1.53) for males, there was a roughly 20% rise in female ASMR during the same period (Figure 6; Supplementary Tables S10–S13).

### 3.7 Future forecasts of global burden

**Figure 8:**
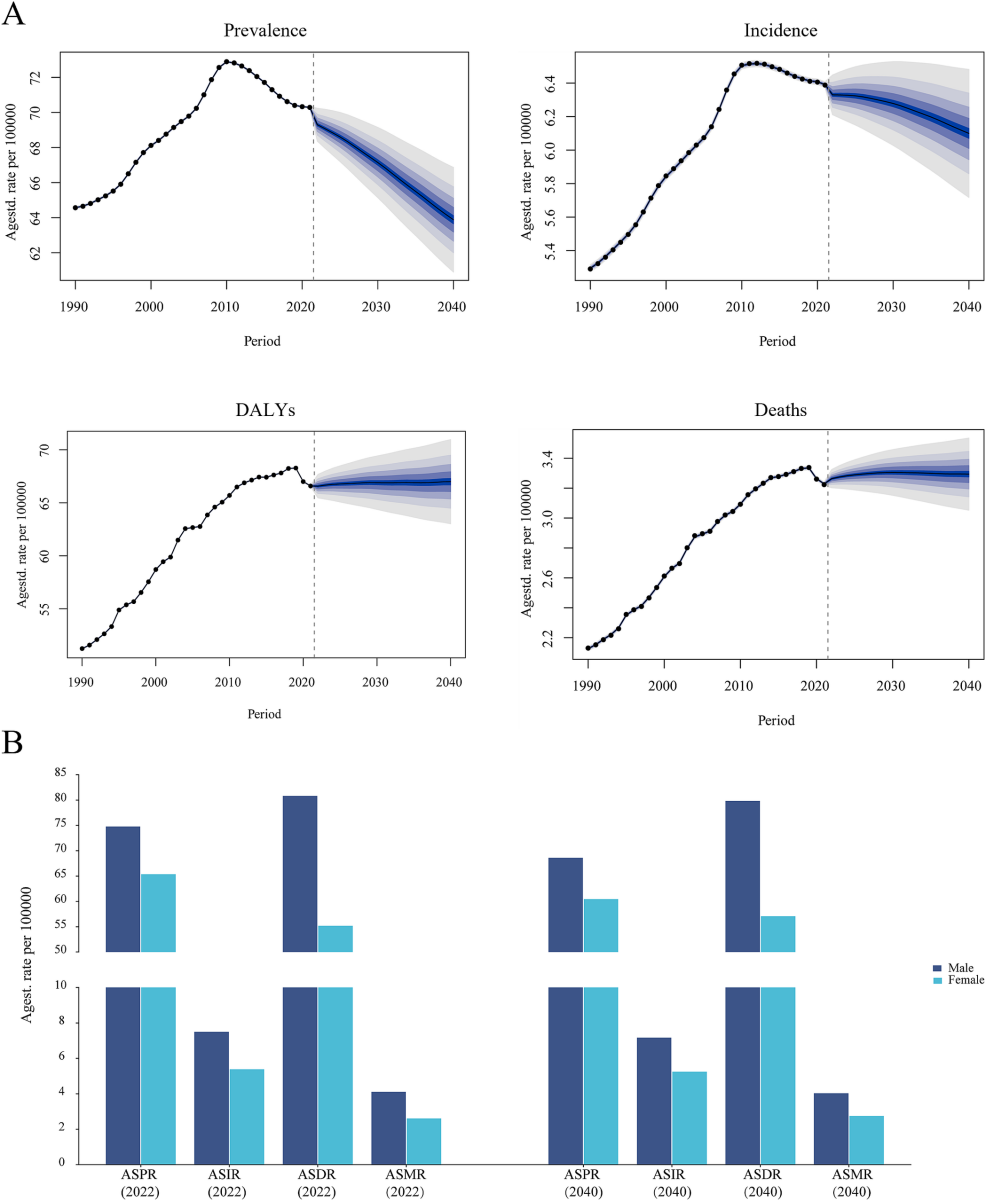
Trends in global burden projections for ILD&PS. **(A)** Prediction of ASR of ILD&PS burdens; **(B)** Predictions of ASR of ILD&PS burden by gender for 2022 and 2040. ILD&PS, Interstitial lung disease and pulmonary sarcoidosis; DALYs, Disability adjusted life years; ASPR, Age-standardized prevalence rate; ASIR, Age-standardized incidence rate; ASDR, Age-standardized DALYs rate; ASMR, Age-standardized mortality rate.

**Figure 9:**
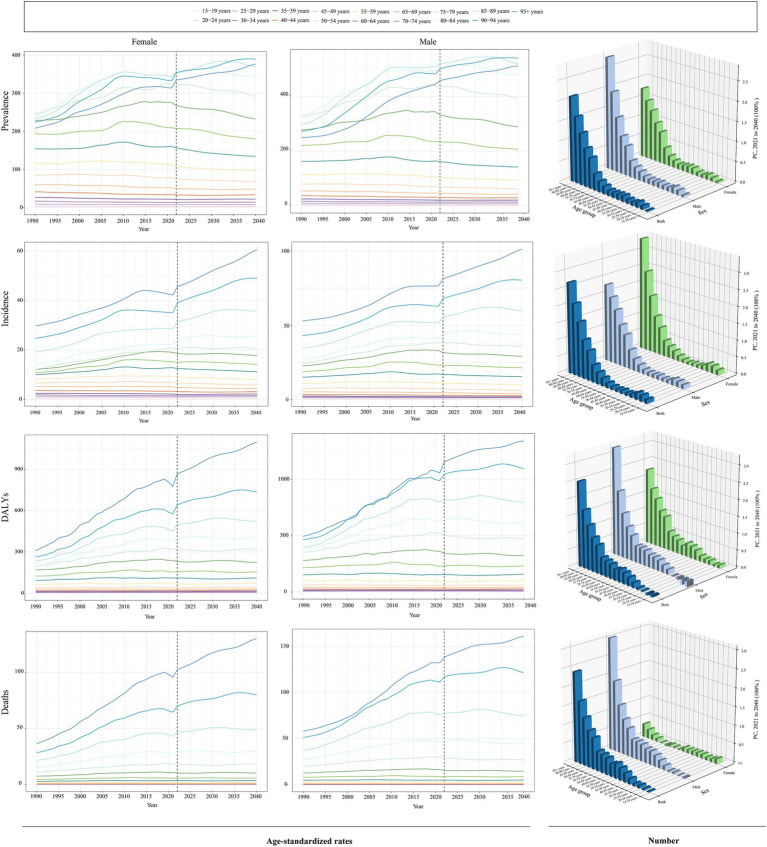
Prediction of ILD&PS burden trends for ILD&PS across age groups. ILD&PS, Interstitial lung disease and pulmonary sarcoidosis; ASPR, Age-standardized prevalence rate; ASIR, Age-standardized incidence rate; ASDR, Age-standardized DALYs rate; ASMR, Age-standardized mortality rate; DALYs, Disability adjusted life years; PC, Percentage change.

Considering that the burden of ILD&PS was almost nil for the 0–14 age group across all age groups, we focused on the global forecasting analysis on age groups beginning at 15 years old. As illustrated in Figure 8 and Supplementary Figure S10, the global case number of ILD&PS burden was predicted to increase. In 2040, it was estimated that there would be 4,650,604 (95% CI, 4,392,112.33 to 4,909,095.67) cases of global prevalence, 444,039.30 (95% CI, 41,081.09 to 477,264.51) cases of incidence, 4,876,548 (95% CI, 4,532,374.34 to 5,220,721.66) cases of DALYs, and approximately 239,787.79 (95% CI, 218,867.23 to 260,708.25) deaths. On the other hand, it was predicted that the ASPR and ASIR would decline yearly until 2040. Overall, it was anticipated that the disease burden for men will continue to be much greater than that for women by 2040 (Figure 8). It was important to note that the age-standardized load for the 90–94 and 95 + age groups was expected to exhibit an increasing trend in both males and females, compared to other age groups. While the ASPR may actually decline, the ASIR, ASDR, and ASMR of ILD&PS for those aged 60–64, 65–69, and 70–74 were predicted to level out in the approaching time. For males aged 15–19, 20–24, 25–29, and 30–34, the predicted data showed a declining trend in the PC of DALYs. Furthermore, from 2022 to 2040, the proportion of males over 50 who passed away from ILD&PS was much higher than that of women (Figure 9).

## 4 Discussion

Patients’ health and lives are seriously threatened by a set of diverse respiratory disorders known as ILD&PS (22). The prevalence, incidence, DALYs, and mortality rates of ILD&PS were presented in this study using data from GBD 2021 and stratified by age, sex, SDI, and geographic location. In addition to forecasting the disease burden status until 2040, it conducted a comprehensive examination of the illness burden over a 32-year period and updated the most recent data. Undoubtedly, these comprehensive assessment data helped policymakers comprehend the issue, which in turn helped them successfully guide the allocation of medical resources and the creation of public health policies.

Globally, ILD&PS-related prevalence, incidence, DALYs, and mortality rose by 128%, 148%, 169% and 242%, respectively, between 1990 and 2021. The burden of ILD&PS decreased while taking into account the measurement levels of ASRs, although it still indicated an upward tendency. The phenomenon can be ascribed to the continuous trend of societal aging and population growth (23). According to our estimates, there may be up to 4.65 million instances of ILD&PS by 2040, with roughly 0.44 million incident cases, a DALYs percentage of 4.88 million, and 0.24 million fatalities. All things considered, the overall disease burden increased over time, placing increasing pressure on international health decision-making and medical service systems.

Consistent with the study by Zeng et.al, the high SDI regions have the highest ASPR in 2021 (3). After age-standardization, high SDI regions remained the top-ranking areas, outperforming the middle SDI regions, despite the fact that the middle SDI regions saw the most changes in prevalence, incidence, and deaths burden. The superior quality and greater accessibility of medical services are correlated with areas with higher SDI scores, which generally show more advanced health system performance (24). Additionally, our findings revealed that the ASRs burden of ILD&PS tended to be positively associated with the SDI level. Over recent years, advancements in the medical sector have provided high SDI regions with more precise diagnostic tools, including computed tomography scans and spirometry, as well as improved diagnostic approaches, such as multidisciplinary discussions (25–27). Furthermore, exposure to occupational hazardous particles and gases generated in industries such as mining, along with drug abuse, and adverse reactions induced by radiotherapy and chemotherapy, serve as significant etiological factors in pulmonary diseases (28). Notably, tobacco smoking has been conclusively established as an independent risk factor for ILDs, particularly IPF (29). These variables have probably contributed to the rise in instances found in high SDI locations. Nevertheless, we found that high SDI regions experienced the greatest increase in ILD&PS-related fatalities despite having sophisticated medical services. Ranganathan, S., *et al*. believe that an aging society is more likely to develop in a nation with a higher GDP because of a tendency toward lower birth rates (30). At the same time, many unsolved risk variables are associated with the onset and progression of ILD&PS, highlighting the need to supplement existing resources to effectively address this challenge (31). Regional and national differences were evident in trends in the burden of disease. For instance, in 2021, the ASPR for ILDs in High-income Asia Pacific was 151.60 per 100,000, but in Western Sub-Saharan Africa, it was just 12.79. According to the regional level research, 95.2% of regions had rising trends in the prevalence of ILD&PS, with Australasia, North Africa and the Middle East, and Andean Latin America showing the biggest shifts. The risk of acquiring ILDs has been increased by metal dust, wood dust, insecticides, and agricultural dusts (32). In particular, ILD&PS prevalence has been found to be higher in areas with unique features, such as Australia with its thriving livestock industry, the oil-rich but conflict-prone Middle East, and mining-rich Andean Latin America (33).

China, India, and the United States of America had the highest incidence and prevalence of ILD&PS in 2021 due to their large populations. Numerous factors, such as population lifestyles, regional environmental differences, and the efficacy of national health programs for illness prevention and control, contribute significantly to this variation (34–36). Air pollution from the extensive use of stationary fuels is a major contributor to chronic respiratory disease, and disease incidence is higher in places with poorer lifestyles and a lack of public health interventions (26). Interestingly, we also found that the burden of ILD&PS in Ecuador, Chile, Bolivia, and Peru was far higher than the SDI had estimated. ILD incidence is closely linked to exposure to air pollutants, such as PM2.5, black carbon, and ozone (36). In a similar vein, Ecuador and Peru have been important South American oil suppliers (37). The GBD 2019 study finds that the growth of mining operations in Chile has a significant impact on the burden of silicosis (38). Bolivians live in adobe brick dwellings, many of which contain physiologically accessible amounts of lead and arsenic that could have a major effect on the population’s health risks (39). Notably, Latvia and Ukraine had generally had a declining tendency, as indicated by EAPC. These patterns might suggest that the people in the area are more health conscious, which lead to early and effective public health initiatives that focus on disease risk factors and enhancements, such as knowledge of the dangers of excessive alcohol and sodium consumption (40, 41). In line with the findings from SDI areas, the EAPC of illness burden generally showed a positive link with the SDI level, and more cases were discovered in nations with greater SDI. Hence, it is crucial to keep a close eye on the trends in these countries, learn from the preventative and treatment approaches of those where the disease burden has decreased, and develop interventions that are especially tailored to each country’s particular situation.

The burden of ILD&PS was most prevalent among people between the ages of 50 and 89, with the burden being highest among those between the ages of 70 and 74. In addition, we found that the degree of PC and EAPC in the burden steadily rose with age during the previous 32 years, with the 95 + age group showing the most notable increases. This underscored how age significantly affects the epidemiology of ILD&PS. Given that the old population has the highest mortality rates from COVID-19, influenza, and related causes, population aging may have an effect on the burden of respiratory disorders (42). The immune system steadily deteriorates with age, and the buildup of chronic inflammatory reactions over time raises the risk factors by a large margin (43). However, comorbidities are becoming more common in older persons, which can make it more difficult to control sarcoidosis and ILD and increase the rates of morbidity and deaths (44, 45). Between 1990 and 2021, medical improvements were the primary cause of the global decline in mortality rates in the 0–14 and 50–54 age groups. Better results and fewer respiratory issues are achieved with improved child care (46). Besides, improved knowledge of the pathophysiology of diseases results in more efficient treatments that reduce symptoms and improve the quality of life for patients in particular age groups (1, 9).

In 2021, the ASR burden of ILD&PS was larger in males than in women, which is in line with earlier research findings regarding gender discrepancies (31). Furthermore, it was predicted that by 2040, the ASR burden of ILD&PS will still be substantially larger for men than for women, with men aged 50 and older having a far higher PC of fatalities than women. This may be attributed to the fact that men have historically smoked more than women, and smoking exacerbating the process of pulmonary fibrosis in the alveolar walls (47, 48). From a sociological perspective, men continue to make up the majority of the labor force today, and they are more likely than women to work on building sites, where they are exposed to harmful particles (49, 50). However, the estrogen exhibits dual pro-inflammatory and anti-fibrotic effects with its postmenopausal decline compromising these protective effects (51). In addition, morphometric analyses reveal sexually dimorphic pulmonary architecture: females possess smaller, prismatic lungs with constrained airway diameters, volumes, and diffusion surfaces versus males’ expanded alveolar numbers and surface areas at matched anthropometrics, providing a structural basis for differential disease susceptibility (52, 53). Similarly, contemporary narrowing of occupational exposure disparities between genders, coupled with sociomedical factors such as frequent misdiagnosis of early symptoms as anxiety or menopausal syndrome, collectively contribute to the cause of the more noticeable ASDR and ASMR alterations in females compared to males (54–56). Currently, the most common malignancy in women is breast cancer, and Trastuzumab Deruxtecan treatment has been found to raise the likelihood of getting ILD (57, 58). In addition, pro-inflammatory and pro-fibrotic factors are greatly influenced by sex hormone control since estrogens encourage remodeling and inflammation while androgens may have the opposite effect (59).

While the number of ILD&PS cases was expected to increase in the near future, the ASPR and ASIR were expected to decline annually. Although this is encouraging, steps must also be taken to lower mortality and DALY rates. A third fewer premature deaths from NCDs are anticipated occur by 2030, according to the Global Alliance against Chronic Respiratory Disease (GARD).^3^ Therefore, public health programs must be designed by the government to target at-risk groups, especially men over 50 and postmenopausal women. In regions with high disease burdens, improving health education, implementing targeted interventions, and streamlining resource distribution will constitute an effective approach to addressing health disparities and mitigating the pervasive impacts of the disease. Furthermore, the predicted relative decline but absolute rise in burden highlighted the need for primary prevention and early screening, alongside targeted interventions and long-term monitoring.

However, our study has several limitations. Firstly, potential misclassification between ILD and pulmonary sarcoidosis in registries may obscure true disease burdens, particularly where histopathological confirmation is limited. Under-ascertainment in low-resource regions could skew SDI correlations, compounded by ecological fallacy risks when extrapolating country-level SDI to individual risk. Secondly, disparities in diagnostic criteria and case-reporting protocols may introduce systematic biases. Finally, our projections were constrained by current available data and likely underestimate the impact of emerging risk exposures.

## 5 Conclusion

In conclusion, our study carefully investigated the incidence, prevalence, DALYs, and mortality associated with ILD&PS at the national, regional, and worldwide levels. The burden of ILD&PS increased globally over the last 32 years, with a considerable increase in high SDI regions and among males. According to our predictions, the ASR stayed the same or even dropped, even while the overall illness burden increased. Actively developing specialized prevention and treatment plans for ILD&PS that addressed the particular requirements of different nations and populations was essential.

## Data Availability

The original contributions presented in the study are included in the article/Supplementary material, further inquiries can be directed to the corresponding authors.

## Footnotes

^1^ https://vizhub.healthdata.org/gbd-results/

^2^ https://ghdx.healthdata.org/record/ihme-data/gbd-2021-cause-icd-code-mappings

^3^ http://www.who.int/nmh/events/ncd_action_plan/en/
