# Supplementary material for: Global, regional and national burden of interstitial lung disease and pulmonary sarcoidosis, 1990–2021 and projection to 2040
Source: Front Med (Lausanne). 2025 Oct 27;12:1650997. doi: 10.3389/fmed.2025.1650997 (PMC12599143; doi:10.3389/fmed.2025.1650997)
Supplement: Supplementary file 1 [file Table_1.docx]

**Table S1. PC of ILD&PS burden, 1990 vs. 2021 (Global, SDI Quintiles and GBD regions).**

| **Location** | **PC of prevalence cases  (100%, 95% UI)** | **PC of incidence cases**  **(100%, 95% UI)** | **PC of DALYs cases  (100%, 95% UI)** | **PC of death cases  (100%, 95% UI)** | **PC of ASPR  (100%, 95% UI)** | **PC of ASIR  (100%, 95% UI)** | **PC of ASDR  (100%, 95% UI)** | **PC of ASMR  (100%, 95% UI)** |
| --- | --- | --- | --- | --- | --- | --- | --- | --- |
| Global | 1.28(1.16-1.42) | 1.48(1.36-1.62) | 1.69(1.35-2.18) | 2.42(1.97-3.02) | 0.09(0.04-0.14) | 0.21(0.15-0.26) | 0.28(0.13-0.50) | 0.50(0.32-0.74) |
| **SDI quintile** |  |  |  |  |  |  |  |  |
| Low SDI | 1.36(1.27-1.46) | 1.38(1.29-1.48) | 1.30(0.63-2.30) | 1.48(0.78-2.56) | 0.03(-0.00-0.08) | 0.04(0.00-0.09) | 0.05(-0.23-0.47) | 0.11(-0.17-0.56) |
| Low-middle SDI | 1.54(1.42-1.66) | 1.52(1.40-1.65) | 1.64(1.08-2.80) | 1.97(1.34-3.33) | 0.11(0.07-0.16) | 0.10(0.07-0.15) | 0.16(-0.07-0.64) | 0.24(-0.01-0.79) |
| Middle SDI | 1.78(1.60-1.97) | 1.93(1.74-2.12) | 2.01(1.46-2.84) | 2.71(1.98-3.78) | 0.16(0.11-0.22) | 0.24(0.19-0.30) | 0.24(0.01-0.56) | 0.29(0.06-0.66) |
| High-middle SDI | 1.00(0.88-1.14) | 1.19(1.06-1.33) | 1.04(0.81-1.28) | 1.77(1.43-2.10) | 0.07(0.01-0.13) | 0.19(0.14-0.25) | 0.07(-0.05-0.19) | 0.31(0.16-0.46) |
| High SDI | 1.14(1.02-1.29) | 1.38(1.23-1.53) | 1.97(1.78-2.14) | 3.07(2.76-3.33) | 0.17(0.12-0.24) | 0.32(0.25-0.39) | 0.53(0.45-0.62) | 0.92(0.81-1.03) |
| **GBD region** |  |  |  |  |  |  |  |  |
| Andean Latin America | 4.00(3.81-4.22) | 3.85(3.64-4.08) | 2.58(1.44-4.32) | 3.41(1.91-5.75) | 0.78(0.71-0.85) | 0.66(0.58-0.75) | 0.32(-0.09-0.95) | 0.45(-0.05-1.22) |
| Australasia | 2.82(2.57-3.11) | 3.25(3.02-3.52) | 4.79(4.26-5.33) | 6.23(5.46-7.01) | 0.69(0.60-0.80) | 0.88(0.79-0.99) | 1.49(1.27-1.70) | 1.80(1.52-2.09) |
| Caribbean | 1.64(1.49-1.84) | 1.55(1.41-1.72) | 2.12(1.36-3.04) | 2.66(1.87-3.63) | 0.37(0.30-0.46) | 0.34(0.29-0.39) | 0.66(0.30-1.11) | 0.70(0.35-1.13) |
| Central Asia | 0.81(0.76-0.87) | 0.70(0.64-0.78) | -0.02(-0.20-0.26) | -0.12(-0.31-0.16) | 0.03(0.00-0.06) | 0.01(-0.03-0.05) | -0.42(-0.53--0.26) | -0.47(-0.59--0.31) |
| Central Europe | 0.25(0.20-0.31) | 0.11(0.05-0.16) | 0.19(0.08-0.31) | 0.44(0.30-0.61) | 0.00(-0.02-0.04) | -0.06(-0.09--0.03) | -0.13(-0.21--0.04) | -0.07(-0.16-0.04) |
| Central Latin America | 2.30(2.12-2.53) | 2.60(2.39-2.84) | 3.70(3.24-4.20) | 4.78(4.20-5.42) | 0.18(0.11-0.24) | 0.33(0.27-0.41) | 0.75(0.58-0.94) | 0.86(0.68-1.06) |
| Central Sub-Saharan Africa | 1.86(1.72-2.01) | 1.79(1.67-1.94) | 1.54(0.61-2.88) | 1.57(0.64-2.83) | 0.11(0.06-0.16) | 0.05(0.01-0.09) | 0.03(-0.31-0.49) | 0.04(-0.31-0.50) |
| East Asia | 1.49(1.27-1.74) | 1.57(1.30-1.83) | 1.03(0.30-1.95) | 1.69(0.51-3.22) | 0.08(0.01-0.17) | 0.22(0.14-0.31) | -0.11(-0.43-0.28) | -0.00(-0.43-0.54) |
| Eastern Europe | -0.42(-0.45--0.39) | -0.50(-0.52--0.47) | -0.57(-0.61--0.53) | -0.60(-0.63--0.56) | -0.47(-0.49--0.43) | -0.50(-0.52--0.47) | -0.63(-0.67--0.60) | -0.70(-0.72--0.67) |
| Eastern Sub-Saharan Africa | 1.60(1.51-1.70) | 1.44(1.36-1.55) | 1.14(0.29-2.52) | 1.16(0.33-2.44) | 0.10(0.07-0.14) | 0.00(-0.03-0.04) | -0.05(-0.39-0.43) | -0.06(-0.39-0.39) |
| High-income Asia Pacific | 1.34(1.16-1.53) | 1.33(1.07-1.59) | 2.27(1.94-2.60) | 4.04(3.46-4.53) | 0.13(0.07-0.19) | 0.27(0.18-0.35) | 0.30(0.19-0.41) | 0.60(0.44-0.75) |
| High-income North America | 1.00(0.89-1.13) | 1.35(1.22-1.49) | 1.76(1.62-1.87) | 2.69(2.47-2.83) | 0.10(0.05-0.16) | 0.29(0.22-0.37) | 0.47(0.40-0.52) | 0.90(0.80-0.97) |
| North Africa and Middle East | 2.85(2.67-3.12) | 2.49(2.31-2.75) | 2.25(1.37-3.83) | 2.32(1.37-4.15) | 0.45(0.38-0.55) | 0.30(0.25-0.37) | 0.24(-0.07-0.80) | 0.19(-0.12-0.82) |
| Oceania | 1.84(1.73-1.97) | 1.70(1.58-1.83) | 1.49(0.57-2.70) | 1.60(0.63-2.90) | 0.16(0.11-0.20) | 0.13(0.08-0.18) | 0.08(-0.29-0.55) | 0.06(-0.32-0.54) |
| South Asia | 1.70(1.58-1.82) | 1.68(1.55-1.81) | 1.77(1.14-3.12) | 2.12(1.42-3.73) | 0.08(0.04-0.13) | 0.07(0.04-0.12) | 0.10(-0.14-0.63) | 0.16(-0.09-0.74) |
| Southeast Asia | 2.25(2.08-2.49) | 1.93(1.77-2.12) | 1.62(1.02-2.59) | 1.85(1.20-3.17) | 0.32(0.26-0.39) | 0.19(0.14-0.25) | 0.12(-0.12-0.50) | 0.13(-0.11-0.66) |
| Southern Latin America | 2.07(1.94-2.21) | 2.08(1.96-2.20) | 2.08(1.82-2.34) | 2.82(2.46-3.20) | 0.66(0.60-0.73) | 0.65(0.60-0.71) | 0.63(0.50-0.76) | 0.86(0.69-1.04) |
| Southern Sub-Saharan Africa | 0.93(0.87-1.00) | 0.87(0.80-0.94) | 0.92(0.49-1.60) | 1.00(0.41-1.78) | -0.09(-0.12--0.06) | -0.10(-0.13--0.07) | -0.04(-0.29-0.26) | -0.04(-0.35-0.40) |
| Tropical Latin America | 0.83(0.63-1.06) | 1.57(1.31-1.87) | 2.72(2.45-2.98) | 4.35(3.88-4.77) | -0.25(-0.32--0.17) | 0.07(-0.01-0.16) | 0.54(0.43-0.64) | 0.87(0.72-1.02) |
| Western Europe | 0.97(0.87-1.09) | 1.30(1.18-1.42) | 2.40(2.16-2.64) | 3.60(3.21-3.91) | 0.26(0.21-0.33) | 0.45(0.40-0.52) | 1.05(0.92-1.17) | 1.51(1.33-1.67) |
| Western Sub-Saharan Africa | 1.23(1.13-1.34) | 1.08(0.98-1.19) | 0.90(0.37-1.68) | 0.79(0.29-1.51) | -0.09(-0.13--0.05) | -0.17(-0.21--0.14) | -0.19(-0.42-0.12) | -0.20(-0.41-0.10) |

PC, Percentage change; ILD&PS, Iinterstitial lung disease and pulmonary sarcoidosis; SDI, Socio-demographic index; DALYs, Disability-adjusted life year; ASPR, Age-standardized prevalence rate; ASIR, Age-standardized incidence rate; ASDR, Age-standardized DALYs rate; ASMR, Age-standardized mortality rate; UI, Uncertainty interval.

**Table S2. Number and age-standardized incidence of ILD&PS, 1990 vs. 2021 (Global, SDI Quintiles and GBD regions).**

| **Location** | **Number in 1990  (95% UI)** | **ASIR in 1990  (per 100 000, 95% UI)** | **Number in 2021  (95% UI)** | **ASIR in 2021  (per 100 000, 95% UI)** | **EAPC of ASIR (95% CI)** |
| --- | --- | --- | --- | --- | --- |
| Global | 157441.17(136251.29-179471.82) | 3.77(3.27-4.28) | 390267.11(346393.42-433403.27) | 4.54(4.05-5.04) | 0.72(0.63-0.82) |
| **SDI quintile** |  |  |  |  |  |
| Low SDI | 7696.02(6515.90-8957.93) | 3.19(2.74-3.64) | 18292.09(16088.70-20678.40) | 3.33(2.95-3.70) | 0.16(0.13-0.18) |
| Low-middle SDI | 28178.89(23956.49-32564.90) | 4.39(3.76-5.00) | 70990.24(62702.07-79388.32) | 4.85(4.28-5.42) | 0.38(0.35-0.41) |
| Middle SDI | 30614.74(26132.25-35606.26) | 2.71(2.35-3.10) | 89561.47(79276.37-100011.02) | 3.37(3.00-3.73) | 0.91(0.81-1.02) |
| High-middle SDI | 25615.73(22401.26-29234.52) | 2.49(2.20-2.83) | 56001.46(50154.74-61921.28) | 2.97(2.68-3.27) | 0.85(0.71-0.99) |
| High SDI | 65228.93(56924.96-73998.17) | 6.20(5.42-7.03) | 155237.81(137458.20-174122.24) | 8.19(7.29-9.07) | 0.92(0.8-1.04) |
| **GBD region** |  |  |  |  |  |
| Andean Latin America | 2439.73(2227.64-2661.72) | 12.29(11.13-13.46) | 11837.69(11093.94-12531.27) | 20.47(19.15-21.69) | 2.13(1.98-2.28) |
| Australasia | 798.39(716.65-884.74) | 3.43(3.08-3.80) | 3392.34(3070.03-3718.02) | 6.45(5.88-7.02) | 2.15(1.94-2.36) |
| Caribbean | 393.61(343.57-448.41) | 1.42(1.25-1.60) | 1003.14(917.01-1094.45) | 1.89(1.73-2.07) | 1.06(0.98-1.14) |
| Central Asia | 1672.44(1504.39-1851.11) | 3.36(3.05-3.70) | 2848.59(2621.60-3093.58) | 3.38(3.12-3.65) | 0.02(-0.2-0.24) |
| Central Europe | 3649.65(3250.81-4100.12) | 2.59(2.30-2.90) | 4033.56(3661.38-4442.55) | 2.42(2.20-2.69) | 0.02(-0.07-0.1) |
| Central Latin America | 3384.22(2931.87-3876.35) | 3.61(3.14-4.10) | 12172.27(10942.78-13423.36) | 4.81(4.33-5.29) | 0.95(0.86-1.03) |
| Central Sub-Saharan Africa | 456.18(384.88-531.22) | 1.80(1.55-2.05) | 1271.70(1103.24-1454.64) | 1.88(1.66-2.10) | 0.17(0.13-0.2) |
| East Asia | 19462.90(16280.98-23141.04) | 1.90(1.60-2.23) | 50030.89(42927.49-57599.83) | 2.31(2.02-2.64) | 1.12(0.87-1.38) |
| Eastern Europe | 5321.51(4509.72-6209.13) | 2.07(1.78-2.40) | 2682.32(2299.15-3096.10) | 1.04(0.89-1.21) | -2.56(-2.68--2.45) |
| Eastern Sub-Saharan Africa | 1327.80(1107.28-1558.83) | 1.52(1.31-1.74) | 3243.79(2779.59-3752.94) | 1.53(1.34-1.72) | -0.02(-0.03--0.01) |
| High-income Asia Pacific | 18818.05(16024.17-22063.87) | 9.15(7.82-10.66) | 43787.31(38399.14-49649.78) | 11.59(10.25-13.02) | 0.79(0.6-0.97) |
| High-income North America | 28333.49(24529.71-32456.44) | 8.48(7.37-9.71) | 66609.12(58422.82-75152.76) | 10.95(9.73-12.20) | 0.75(0.62-0.88) |
| North Africa and Middle East | 4326.19(3719.78-5016.18) | 2.19(1.91-2.49) | 15113.49(13573.57-16830.54) | 2.85(2.58-3.15) | 0.98(0.93-1.03) |
| Oceania | 155.31(138.59-173.87) | 3.72(3.37-4.09) | 419.30(384.88-456.21) | 4.20(3.91-4.51) | 0.39(0.36-0.41) |
| South Asia | 35968.28(30515.17-41709.48) | 6.01(5.11-6.88) | 96281.45(84137.37-108635.45) | 6.44(5.65-7.26) | 0.25(0.22-0.28) |
| Southeast Asia | 3838.79(3217.54-4512.25) | 1.36(1.16-1.56) | 11245.44(9856.84-12737.72) | 1.62(1.42-1.81) | 0.54(0.53-0.56) |
| Southern Latin America | 2749.81(2529.38-2974.59) | 5.99(5.51-6.47) | 8476.90(7913.38-9062.32) | 9.90(9.26-10.57) | 1.68(1.53-1.82) |
| Southern Sub-Saharan Africa | 1380.60(1182.36-1597.13) | 4.68(4.00-5.36) | 2577.13(2241.39-2928.29) | 4.21(3.69-4.73) | -0.52(-0.68--0.36) |
| Tropical Latin America | 2580.47(2183.94-3040.08) | 2.44(2.08-2.82) | 6639.97(5821.18-7441.33) | 2.62(2.29-2.94) | 0.09(0.02-0.16) |
| Western Europe | 18947.57(17006.94-20973.69) | 3.64(3.26-4.05) | 43619.93(39614.12-47748.76) | 5.30(4.82-5.81) | 1.46(1.29-1.62) |
| Western Sub-Saharan Africa | 1436.21(1203.76-1682.08) | 1.37(1.17-1.58) | 2980.76(2544.17-3485.63) | 1.13(0.99-1.28) | -0.65(-0.71--0.58) |

ILD&PS, Interstitial lung disease and pulmonary sarcoidosis; SDI, Socio-demographic index; ASIR, Age-standardized incidence rate; EAPC, Estimated annual percentage change; UI, Uncertainty interval; CI, Confidence interval.

**Table S3. Number and age-standardized DALYs of ILD&PS, 1990 vs. 2021 (Global, SDI Quintiles and GBD regions).**

| **Location** | **Number in 1990  (95% UI)** | **ASDR in 1990  (per 100 000, 95% UI)** | **Number in 2021  (95% UI)** | **ASDR in 2021  (per 100 000, 95% UI)** | **EAPC of ASDR (95% CI)** |
| --- | --- | --- | --- | --- | --- |
| Global | 1501028.43(1221196.88-1850556.94) | 37.15(30.62-45.37) | 4042150.49(3489794.64-4516882.92) | 47.62(41.26-53.16) | 0.95(0.86-1.05) |
| **SDI quintile** |  |  |  |  |  |
| Low SDI | 126670.88(60835.33-188619.30) | 53.16(27.07-75.28) | 291854.54(178639.62-406439.75) | 56.02(34.43-78.83) | 0.31(0.21-0.42) |
| Low-middle SDI | 359489.06(213851.92-552928.69) | 57.35(34.67-86.88) | 949437.88(662365.39-1260418.62) | 66.55(46.34-88.36) | 0.64(0.57-0.71) |
| Middle SDI | 269855.74(212977.03-366218.84) | 25.08(19.94-33.57) | 812056.09(695675.28-984261.99) | 31.00(26.55-37.41) | 0.83(0.76-0.91) |
| High-middle SDI | 237514.38(213560.48-266885.04) | 23.85(21.53-26.81) | 485511.72(430895.70-541669.29) | 25.48(22.63-28.47) | 0.41(0.32-0.5) |
| High SDI | 506204.64(470841.19-546440.49) | 46.54(43.29-50.27) | 1500929.60(1354746.32-1614665.30) | 71.40(65.27-76.57) | 1.54(1.34-1.74) |
| **GBD region** |  |  |  |  |  |
| Andean Latin America | 33944.99(24797.83-48279.53) | 158.26(116.08-221.60) | 121673.13(96602.28-150014.43) | 209.34(165.81-257.66) | 1.47(1.29-1.64) |
| Australasia | 5708.10(5219.08-6190.00) | 24.12(22.11-26.10) | 33035.99(28955.57-35886.01) | 59.97(53.13-64.90) | 3.1(2.56-3.64) |
| Caribbean | 5039.73(3866.34-6913.75) | 18.07(14.09-23.62) | 15715.28(12779.33-19980.94) | 30.00(24.15-38.60) | 1.92(1.74-2.11) |
| Central Asia | 20131.18(18089.07-22762.62) | 40.30(35.89-45.69) | 19790.21(16339.43-24518.20) | 23.37(19.37-28.78) | -1.98(-2.38--1.59) |
| Central Europe | 44911.61(41926.37-48292.81) | 30.89(28.89-33.12) | 53275.24(48498.57-58218.19) | 26.90(24.41-29.45) | -0.13(-0.37-0.12) |
| Central Latin America | 33622.55(31641.73-35883.90) | 35.89(33.81-38.25) | 158077.20(143505.43-173854.76) | 62.84(57.11-69.10) | 1.92(1.73-2.11) |
| Central Sub-Saharan Africa | 8097.07(2924.87-15523.97) | 32.71(12.32-68.73) | 20544.53(8466.67-44098.94) | 33.84(13.17-79.52) | 0.09(-0.01-0.19) |
| East Asia | 115445.09(86137.56-171273.59) | 12.41(9.41-18.31) | 234265.80(171304.55-301444.13) | 11.01(8.06-14.14) | -0.12(-0.27-0.03) |
| Eastern Europe | 79278.83(72135.69-86326.29) | 29.38(26.78-31.98) | 34251.59(31150.63-37997.43) | 10.78(9.78-12.00) | -4.56(-5.23--3.87) |
| Eastern Sub-Saharan Africa | 22391.04(7514.98-36231.90) | 24.77(9.30-43.15) | 47826.03(19641.78-93157.05) | 23.45(9.36-46.54) | -0.29(-0.34--0.24) |
| High-income Asia Pacific | 132227.16(120398.34-145561.20) | 66.26(60.19-72.90) | 432702.51(380225.14-475662.83) | 86.32(76.64-94.93) | 0.71(0.52-0.89) |
| High-income North America | 210856.16(195639.69-227582.52) | 61.65(57.12-66.57) | 582574.85(532853.22-621774.91) | 90.44(83.28-96.54) | 1.28(1-1.56) |
| North Africa and Middle East | 30115.40(21398.45-44297.82) | 16.19(11.54-23.72) | 97814.45(75696.13-134362.62) | 20.11(15.54-28.24) | 0.95(0.81-1.09) |
| Oceania | 3218.84(2152.51-4772.89) | 69.39(47.61-104.63) | 8024.27(5581.37-12403.40) | 74.63(50.21-118.43) | 0.25(0.17-0.33) |
| South Asia | 473027.60(268377.06-740982.72) | 81.68(47.16-126.64) | 1312643.60(890805.92-1740638.78) | 89.80(61.01-118.92) | 0.4(0.33-0.47) |
| Southeast Asia | 22494.86(12086.73-47186.40) | 8.01(4.35-16.31) | 59047.92(34100.11-109401.61) | 8.93(5.18-16.51) | 0.37(0.32-0.42) |
| Southern Latin America | 27988.58(26106.44-29940.29) | 60.45(56.32-64.62) | 86238.55(79827.81-92166.39) | 98.58(91.54-105.26) | 1.82(1.53-2.12) |
| Southern Sub-Saharan Africa | 13659.45(8255.80-18871.11) | 47.13(27.18-67.43) | 26228.26(18454.68-34420.40) | 45.01(31.73-59.13) | -0.32(-0.55--0.08) |
| Tropical Latin America | 27651.28(26178.37-29362.87) | 26.41(24.86-28.11) | 102784.24(95498.42-108206.43) | 40.58(37.64-42.74) | 1.33(1.06-1.6) |
| Western Europe | 154696.91(143922.78-165738.61) | 27.51(25.55-29.62) | 526089.80(478285.85-559276.08) | 56.37(51.97-59.88) | 3(2.66-3.33) |
| Western Sub-Saharan Africa | 36521.98(13622.67-55767.25) | 38.64(15.12-59.97) | 69547.03(29750.72-115085.42) | 31.33(13.04-52.94) | -0.61(-0.73--0.5) |

ILD&PS, Interstitial lung disease and pulmonary sarcoidosis; SDI, Socio-demographic index; DALYs, Disability-adjusted life years; ASDR, Age-standardized DALYs rate; EAPC, Estimated annual percentage change; UI, Uncertainty interval; CI, Confidence interval.

**Table S4. Number and age-standardized mortality of ILD&PS, 1990 vs. 2021 (Global, SDI Quintiles and GBD regions).**

| **Location** | **Number in 1990  (95% UI)** | **ASMR in 1990  (per 100 000, 95% UI)** | **Number in 2021  (95% UI)** | **ASMR in 2021 (per 100 000, 95% UI)** | **EAPC of ASMR (95% CI)** |
| --- | --- | --- | --- | --- | --- |
| Global | 54967.23(44761.39-68391.19) | 1.52(1.25-1.87) | 188222.37(161405.66-212251.52) | 2.28(1.96-2.56) | 1.55(1.42-1.69) |
| **SDI quintile** |  |  |  |  |  |
| Low SDI | 4477.69(2158.18-6437.86) | 2.34(1.19-3.27) | 11083.58(6589.04-15793.99) | 2.61(1.56-3.75) | 0.63(0.45-0.81) |
| Low-middle SDI | 13152.92(7523.03-20491.26) | 2.48(1.46-3.81) | 39117.86(26539.67-53611.72) | 3.09(2.12-4.23) | 0.94(0.83-1.06) |
| Middle SDI | 8992.62(7004.00-12322.48) | 1.07(0.85-1.45) | 33341.76(27822.48-40427.28) | 1.39(1.16-1.68) | 1.15(1.03-1.27) |
| High-middle SDI | 8237.68(7611.50-9226.60) | 0.91(0.84-1.02) | 22850.83(20008.19-25163.44) | 1.19(1.04-1.31) | 1.24(1.09-1.39) |
| High SDI | 20063.97(18621.72-20871.04) | 1.79(1.66-1.86) | 81732.30(71243.83-88091.88) | 3.44(3.05-3.69) | 2.3(2.06-2.55) |
| **GBD region** |  |  |  |  |  |
| Andean Latin America | 1443.63(1044.94-2077.78) | 7.86(5.68-11.33) | 6366.23(4874.54-8022.42) | 11.37(8.69-14.33) | 1.87(1.69-2.05) |
| Australasia | 263.42(241.61-284.77) | 1.13(1.03-1.22) | 1903.78(1602.54-2087.52) | 3.17(2.69-3.46) | 3.54(3-4.09) |
| Caribbean | 174.74(140.89-218.99) | 0.70(0.57-0.86) | 639.27(534.39-773.28) | 1.19(0.99-1.45) | 1.99(1.78-2.2) |
| Central Asia | 714.98(616.27-821.27) | 1.64(1.38-1.91) | 629.81(514.54-778.72) | 0.87(0.71-1.06) | -2.05(-2.49--1.62) |
| Central Europe | 1513.16(1419.94-1622.21) | 1.04(0.98-1.12) | 2179.08(1973.34-2369.33) | 0.97(0.88-1.06) | 0.12(-0.21-0.45) |
| Central Latin America | 1119.93(1063.97-1191.02) | 1.44(1.36-1.54) | 6475.43(5836.75-7126.25) | 2.69(2.42-2.96) | 2.19(1.97-2.41) |
| Central Sub-Saharan Africa | 258.94(87.56-548.90) | 1.42(0.49-3.37) | 665.97(241.53-1621.74) | 1.47(0.51-3.92) | 0.09(-0.01-0.19) |
| East Asia | 3042.05(2259.02-4830.81) | 0.41(0.31-0.66) | 8189.68(5177.14-10926.66) | 0.41(0.25-0.54) | 0.38(0.2-0.57) |
| Eastern Europe | 2860.22(2659.61-3059.91) | 1.10(1.02-1.18) | 1153.55(1054.15-1263.43) | 0.34(0.31-0.37) | -5.47(-6.36--4.58) |
| Eastern Sub-Saharan Africa | 686.51(228.31-1205.91) | 1.03(0.35-1.93) | 1483.87(530.27-3097.87) | 0.96(0.33-2.08) | -0.32(-0.37--0.26) |
| High-income Asia Pacific | 5195.84(4709.08-5607.99) | 2.72(2.45-2.94) | 26166.44(21825.75-28825.68) | 4.36(3.73-4.76) | 1.35(1.12-1.58) |
| High-income North America | 8065.65(7441.86-8420.53) | 2.23(2.07-2.33) | 29737.31(26085.08-31529.35) | 4.25(3.75-4.49) | 2.16(1.81-2.52) |
| North Africa and Middle East | 919.07(633.26-1433.16) | 0.64(0.44-1.00) | 3051.40(2249.15-4527.05) | 0.76(0.56-1.15) | 0.87(0.68-1.06) |
| Oceania | 71.24(47.88-109.64) | 2.16(1.45-3.46) | 185.38(118.67-304.19) | 2.30(1.42-3.85) | 0.23(0.17-0.3) |
| South Asia | 17522.10(9598.19-27912.60) | 3.62(2.00-5.65) | 54724.11(35863.11-74242.87) | 4.19(2.81-5.76) | 0.67(0.54-0.79) |
| Southeast Asia | 658.72(317.29-1480.21) | 0.29(0.14-0.66) | 1876.58(958.52-3772.33) | 0.33(0.17-0.66) | 0.42(0.34-0.5) |
| Southern Latin America | 1140.17(1066.43-1222.72) | 2.58(2.41-2.77) | 4356.53(3905.66-4680.94) | 4.80(4.31-5.15) | 2.29(1.92-2.67) |
| Southern Sub-Saharan Africa | 493.34(261.57-751.92) | 2.08(1.07-3.25) | 986.05(677.24-1308.93) | 2.00(1.39-2.65) | -0.34(-0.58--0.1) |
| Tropical Latin America | 846.58(799.38-890.23) | 0.98(0.90-1.04) | 4527.09(4080.06-4835.63) | 1.83(1.64-1.96) | 2.13(1.8-2.46) |
| Western Europe | 6644.62(6203.76-6943.69) | 1.11(1.04-1.16) | 30538.32(26844.52-32788.12) | 2.79(2.49-2.97) | 3.74(3.39-4.09) |
| Western Sub-Saharan Africa | 1332.32(486.02-2109.00) | 1.73(0.66-2.77) | 2386.49(919.03-4115.00) | 1.39(0.54-2.41) | -0.64(-0.75--0.52) |

ILD&PS, Interstitial lung disease and pulmonary sarcoidosis; SDI, Socio-demographic index; ASMR, Age-standardized mortality rate; EAPC, Estimated annual percentage change; UI, Uncertainty interval; CI, Confidence interval.

**Table S5. PC of ILD&PS burden, 1990 vs. 2021 (Nations).**

| **Location** | **PC of prevalence cases  (100%, 95% UI)** | **PC of incidence cases (100%, 95% UI)** | **PC of DALYs cases  (100%, 95% UI)** | **PC of death cases  (100%, 95% UI)** | **PC of ASPR  (100%, 95% UI)** | **PC of ASIR  (100%, 95% UI)** | **PC of ASDR  (100%, 95% UI)** | **PC of ASMR  (100%, 95% UI)** |
| --- | --- | --- | --- | --- | --- | --- | --- | --- |
| Afghanistan | 1.25(1.05-1.45) | 1.34(1.10-1.59) | 3.01(1.42-4.85) | 4.75(3.15-46.11) | 0.17(0.10-0.24) | 0.07(0.01-0.12) | 1.30(0.25-2.45) | 3.04(1.92-33.61) |
| Albania | 0.65(0.55-0.76) | 0.30(0.21-0.42) | 0.33(-0.23-1.41) | 0.61(-0.18-2.09) | 0.01(-0.03-0.05) | -0.12(-0.15--0.09) | -0.27(-0.58-0.31) | -0.29(-0.64-0.37) |
| Algeria | 2.65(2.43-2.92) | 2.24(2.02-2.53) | 4.67(2.47-8.54) | 13.64(7.89-115.04) | 0.29(0.22-0.38) | 0.16(0.10-0.24) | 1.16(0.25-2.64) | 3.97(2.04-42.53) |
| American Samoa | 0.35(0.28-0.43) | 0.19(0.12-0.27) | -0.08(-0.43-0.55) | 0.14(-0.33-0.98) | -0.15(-0.18--0.12) | -0.15(-0.18--0.12) | -0.31(-0.58-0.14) | -0.32(-0.60-0.18) |
| Andorra | 1.86(1.72-2.02) | 1.93(1.78-2.10) | 1.33(0.20-3.00) | 1.71(0.13-4.03) | 0.13(0.09-0.18) | 0.16(0.12-0.22) | -0.11(-0.54-0.51) | -0.10(-0.62-0.66) |
| Angola | 2.83(2.61-3.07) | 2.40(2.21-2.63) | 1.54(0.46-3.73) | 1.59(0.49-3.77) | 0.25(0.18-0.33) | 0.04(-0.02-0.11) | -0.14(-0.49-0.50) | -0.13(-0.50-0.52) |
| Antigua and Barbuda | 2.01(1.83-2.25) | 1.91(1.69-2.13) | 3.20(2.78-3.69) | 2.95(2.47-3.50) | 0.44(0.35-0.55) | 0.49(0.40-0.60) | 1.29(1.06-1.55) | 1.46(1.17-1.82) |
| Argentina | 1.43(1.30-1.56) | 1.50(1.37-1.64) | 1.27(1.01-1.55) | 1.74(1.39-2.15) | 0.43(0.35-0.50) | 0.44(0.37-0.52) | 0.30(0.16-0.45) | 0.46(0.28-0.67) |
| Armenia | 0.53(0.46-0.61) | 0.56(0.46-0.66) | -0.26(-0.36--0.13) | 0.04(-0.11-0.21) | 0.05(0.01-0.09) | 0.07(0.03-0.12) | -0.47(-0.54--0.39) | -0.37(-0.45--0.27) |
| Australia | 3.27(2.99-3.59) | 3.62(3.38-3.89) | 5.15(4.54-5.80) | 6.60(5.71-7.53) | 0.87(0.77-0.99) | 1.03(0.94-1.13) | 1.61(1.36-1.87) | 1.88(1.55-2.21) |
| Austria | 0.43(0.36-0.50) | 0.51(0.43-0.60) | 1.71(1.43-2.02) | 2.72(2.32-3.12) | -0.03(-0.07-0.01) | 0.04(-0.00-0.09) | 0.76(0.58-0.96) | 1.28(1.06-1.52) |
| Azerbaijan | 1.15(1.08-1.22) | 0.93(0.84-1.02) | 0.25(-0.30-1.47) | 0.17(-0.43-1.71) | 0.06(0.03-0.09) | 0.02(-0.02-0.05) | -0.36(-0.64-0.26) | -0.40(-0.71-0.40) |
| Bahamas | 2.31(2.11-2.54) | 2.16(1.94-2.36) | 4.02(2.84-5.47) | 4.57(3.32-6.11) | 0.33(0.27-0.41) | 0.33(0.27-0.39) | 1.14(0.65-1.74) | 1.24(0.76-1.85) |
| Bahrain | 6.43(5.91-7.07) | 6.10(5.54-6.81) | 4.59(1.80-9.08) | 4.81(1.67-10.28) | 0.65(0.56-0.75) | 0.61(0.53-0.69) | 0.23(-0.38-1.21) | 0.30(-0.37-1.45) |
| Bangladesh | 2.80(2.61-2.98) | 1.98(1.85-2.11) | 1.38(0.33-3.42) | 1.55(0.38-3.76) | 0.34(0.28-0.40) | 0.05(0.01-0.09) | -0.16(-0.53-0.55) | -0.13(-0.52-0.56) |
| Barbados | 1.47(1.33-1.64) | 1.48(1.34-1.63) | 2.36(1.68-3.15) | 2.52(1.79-3.35) | 0.40(0.32-0.50) | 0.44(0.37-0.51) | 1.00(0.60-1.48) | 1.12(0.69-1.60) |
| Belarus | -0.49(-0.52--0.45) | -0.62(-0.64--0.60) | -0.75(-0.80--0.69) | -0.82(-0.86--0.77) | -0.52(-0.56--0.49) | -0.60(-0.63--0.58) | -0.78(-0.83--0.73) | -0.86(-0.89--0.83) |
| Belgium | 1.10(0.99-1.22) | 1.22(1.11-1.32) | 1.96(1.71-2.22) | 2.51(2.16-2.86) | 0.44(0.37-0.52) | 0.52(0.45-0.58) | 1.00(0.85-1.16) | 1.15(0.96-1.34) |
| Belize | 6.06(5.61-6.56) | 5.13(4.77-5.53) | 5.41(4.36-6.75) | 5.28(4.06-6.76) | 1.08(0.95-1.21) | 0.86(0.76-0.98) | 1.16(0.78-1.64) | 1.13(0.68-1.66) |
| Benin | 1.61(1.47-1.76) | 1.33(1.19-1.47) | 0.87(0.13-1.91) | 0.69(0.04-1.56) | -0.11(-0.15--0.06) | -0.23(-0.27--0.19) | -0.31(-0.58-0.05) | -0.32(-0.59-0.02) |
| Bermuda | 1.95(1.80-2.12) | 1.76(1.63-1.88) | 1.69(1.12-2.43) | 2.30(1.56-3.28) | 0.51(0.45-0.57) | 0.39(0.35-0.43) | 0.32(0.04-0.68) | 0.32(0.03-0.70) |
| Bhutan | 3.13(2.88-3.41) | 2.42(2.23-2.65) | 1.45(0.48-3.93) | 2.08(0.85-5.44) | 0.79(0.69-0.90) | 0.41(0.34-0.49) | 0.03(-0.36-1.01) | 0.10(-0.32-1.13) |
| Bolivia (Plurinational State of) | 3.43(3.22-3.67) | 3.25(3.06-3.47) | 1.92(0.78-4.42) | 2.37(1.09-5.32) | 0.60(0.53-0.68) | 0.47(0.40-0.55) | 0.11(-0.30-1.02) | 0.18(-0.26-1.11) |
| Bosnia and Herzegovina | 0.14(0.07-0.22) | -0.12(-0.18--0.04) | -0.09(-0.44-0.54) | 0.09(-0.42-1.16) | -0.01(-0.06-0.04) | -0.17(-0.21--0.12) | -0.32(-0.58-0.15) | -0.34(-0.65-0.31) |
| Botswana | 2.17(2.01-2.35) | 1.77(1.61-1.96) | 0.84(0.19-1.99) | 0.80(0.11-2.07) | 0.18(0.12-0.25) | -0.01(-0.06-0.05) | -0.29(-0.53-0.14) | -0.33(-0.57-0.15) |
| Brazil | 0.81(0.61-1.04) | 1.56(1.29-1.86) | 2.72(2.46-2.98) | 4.38(3.89-4.80) | -0.26(-0.33--0.18) | 0.07(-0.02-0.15) | 0.54(0.43-0.65) | 0.88(0.72-1.03) |
| Brunei Darussalam | 1.65(1.52-1.81) | 1.62(1.47-1.80) | 1.39(0.68-2.47) | 1.40(0.56-2.75) | -0.19(-0.23--0.14) | -0.20(-0.24--0.15) | -0.23(-0.47-0.09) | -0.22(-0.48-0.22) |
| Bulgaria | -0.23(-0.26--0.20) | -0.34(-0.37--0.31) | 0.13(-0.07-0.39) | 0.39(0.11-0.79) | -0.18(-0.21--0.14) | -0.25(-0.28--0.23) | 0.10(-0.09-0.35) | 0.14(-0.10-0.45) |
| Burkina Faso | 1.15(1.02-1.28) | 0.97(0.85-1.10) | 0.77(0.20-1.67) | 0.70(0.10-1.62) | -0.10(-0.14--0.05) | -0.22(-0.26--0.18) | -0.22(-0.49-0.15) | -0.24(-0.51-0.14) |
| Burundi | 1.33(1.21-1.45) | 1.29(1.19-1.42) | 0.63(-0.16-2.15) | 0.61(-0.20-1.93) | 0.01(-0.03-0.07) | 0.00(-0.04-0.06) | -0.19(-0.58-0.41) | -0.17(-0.57-0.43) |
| Cabo Verde | 1.91(1.72-2.14) | 1.55(1.34-1.77) | -0.16(-0.44-0.44) | -0.23(-0.49-0.41) | 0.24(0.16-0.33) | 0.10(0.05-0.18) | -0.57(-0.72--0.27) | -0.57(-0.72--0.21) |
| Cambodia | 2.58(2.35-2.91) | 2.08(1.88-2.34) | 1.92(1.02-3.70) | 2.10(0.67-5.83) | 0.37(0.29-0.46) | 0.16(0.10-0.23) | 0.16(-0.16-0.76) | 0.18(-0.31-1.50) |
| Cameroon | 1.69(1.53-1.87) | 1.46(1.31-1.61) | 1.13(0.38-2.19) | 0.96(0.30-1.94) | -0.15(-0.20--0.11) | -0.26(-0.30--0.22) | -0.31(-0.54-0.02) | -0.34(-0.55--0.03) |
| Canada | 2.03(1.85-2.24) | 2.44(2.24-2.66) | 2.59(2.27-2.90) | 3.71(3.22-4.19) | 0.43(0.36-0.51) | 0.64(0.57-0.71) | 0.61(0.48-0.75) | 0.93(0.74-1.11) |
| Central African Republic | 0.97(0.87-1.08) | 0.97(0.88-1.08) | 0.92(0.33-1.71) | 0.84(0.29-1.59) | -0.05(-0.09--0.01) | -0.05(-0.09--0.01) | -0.05(-0.32-0.32) | -0.06(-0.36-0.34) |
| Chad | 1.26(1.12-1.41) | 1.08(0.95-1.22) | 0.91(0.28-1.88) | 0.65(0.07-1.56) | -0.06(-0.11--0.01) | -0.17(-0.21--0.13) | -0.14(-0.43-0.30) | -0.17(-0.48-0.29) |
| Chile | 3.65(3.41-3.90) | 3.37(3.18-3.56) | 3.88(3.52-4.26) | 5.09(4.57-5.68) | 0.86(0.78-0.95) | 0.75(0.69-0.81) | 0.92(0.79-1.06) | 1.15(0.98-1.36) |
| China | 1.47(1.24-1.71) | 1.55(1.28-1.82) | 0.97(0.24-1.89) | 1.60(0.39-3.14) | 0.07(-0.00-0.16) | 0.21(0.14-0.30) | -0.14(-0.46-0.25) | -0.04(-0.47-0.50) |
| Colombia | 3.35(2.98-3.89) | 3.05(2.70-3.49) | 5.92(4.85-6.99) | 8.77(7.12-10.33) | 0.57(0.46-0.73) | 0.53(0.43-0.66) | 1.59(1.20-1.98) | 1.98(1.49-2.46) |
| Comoros | 1.41(1.30-1.53) | 1.26(1.15-1.39) | 0.79(0.05-2.16) | 0.97(0.18-2.36) | -0.00(-0.05-0.04) | -0.07(-0.12--0.02) | -0.21(-0.50-0.26) | -0.22(-0.51-0.27) |
| Congo | 2.24(2.03-2.44) | 1.95(1.75-2.17) | 1.20(0.40-2.92) | 1.11(0.34-2.74) | 0.19(0.12-0.26) | 0.05(0.00-0.11) | -0.16(-0.44-0.41) | -0.17(-0.44-0.37) |
| Cook Islands | 0.65(0.57-0.73) | 0.31(0.23-0.39) | -0.08(-0.47-0.42) | 0.26(-0.33-1.01) | 0.06(0.02-0.10) | -0.08(-0.11--0.05) | -0.30(-0.60-0.11) | -0.30(-0.62-0.12) |
| Costa Rica | 3.26(3.03-3.47) | 3.24(3.02-3.45) | 3.58(3.02-4.18) | 4.34(3.66-5.12) | 0.40(0.33-0.46) | 0.42(0.36-0.48) | 0.60(0.40-0.81) | 0.66(0.44-0.90) |
| Croatia | 0.23(0.14-0.33) | 0.03(-0.05-0.11) | 0.43(0.26-0.62) | 0.96(0.70-1.25) | 0.16(0.08-0.25) | 0.09(0.03-0.16) | 0.11(-0.01-0.24) | 0.20(0.04-0.38) |
| Cuba | 0.80(0.67-0.98) | 0.61(0.46-0.78) | 1.78(1.41-2.20) | 2.83(2.24-3.52) | 0.10(0.03-0.19) | 0.05(-0.01-0.12) | 0.63(0.42-0.88) | 0.93(0.63-1.26) |
| Cyprus | 1.56(1.41-1.71) | 1.55(1.39-1.73) | 0.79(0.14-2.08) | 0.85(0.11-2.48) | 0.04(-0.01-0.10) | 0.03(-0.02-0.09) | -0.38(-0.60-0.03) | -0.44(-0.65--0.01) |
| Czechia | 1.49(1.31-1.74) | 1.57(1.36-1.81) | 1.69(1.25-2.15) | 2.73(2.02-3.54) | 0.75(0.65-0.91) | 0.89(0.77-1.04) | 0.69(0.43-0.97) | 1.19(0.80-1.65) |
| Côte d'Ivoire | 1.54(1.39-1.71) | 1.25(1.12-1.40) | 1.00(0.30-2.04) | 1.01(0.30-1.99) | -0.11(-0.16--0.06) | -0.23(-0.27--0.18) | -0.30(-0.54-0.01) | -0.33(-0.55--0.04) |
| Democratic People's Republic of Korea | 0.97(0.86-1.08) | 0.86(0.76-0.96) | 1.13(0.56-1.87) | 1.46(0.70-2.59) | -0.00(-0.05-0.05) | 0.01(-0.03-0.06) | 0.08(-0.19-0.43) | 0.09(-0.22-0.55) |
| Democratic Republic of the Congo | 1.67(1.54-1.82) | 1.69(1.57-1.85) | 1.65(0.60-3.09) | 1.72(0.59-3.16) | 0.08(0.03-0.13) | 0.05(0.01-0.10) | 0.12(-0.31-0.65) | 0.13(-0.35-0.73) |
| Denmark | 0.73(0.66-0.82) | 0.76(0.67-0.85) | 1.81(1.52-2.16) | 2.58(2.17-3.06) | 0.21(0.17-0.27) | 0.24(0.19-0.29) | 0.84(0.67-1.05) | 1.29(1.04-1.58) |
| Djibouti | 3.82(3.55-4.12) | 3.43(3.16-3.73) | 2.41(1.05-4.82) | 2.66(1.24-5.19) | 0.08(0.03-0.13) | 0.01(-0.05-0.06) | -0.19(-0.48-0.27) | -0.21(-0.49-0.24) |
| Dominica | 0.94(0.83-1.09) | 0.98(0.85-1.12) | 1.05(0.39-2.04) | 0.99(0.30-2.02) | 0.38(0.30-0.48) | 0.46(0.38-0.54) | 0.54(0.04-1.26) | 0.50(-0.02-1.26) |
| Dominican Republic | 2.42(2.17-2.77) | 2.14(1.88-2.47) | 2.51(0.68-6.63) | 3.10(0.76-8.83) | 0.46(0.37-0.57) | 0.39(0.31-0.49) | 0.51(-0.25-2.28) | 0.41(-0.40-2.42) |
| Ecuador | 4.78(4.37-5.18) | 4.66(4.31-5.01) | 5.96(4.54-7.67) | 7.05(5.31-9.20) | 0.94(0.82-1.07) | 0.83(0.72-0.96) | 1.36(0.87-1.96) | 1.48(0.96-2.11) |
| Egypt | 2.31(2.13-2.57) | 1.96(1.80-2.18) | 0.60(0.09-1.26) | 0.43(-0.05-1.17) | 0.46(0.38-0.56) | 0.26(0.20-0.34) | -0.28(-0.48-0.00) | -0.34(-0.54--0.04) |
| El Salvador | 2.46(2.22-2.69) | 2.38(2.17-2.58) | 1.28(0.49-2.48) | 1.65(0.64-3.35) | 0.73(0.62-0.84) | 0.70(0.60-0.78) | 0.21(-0.22-0.86) | 0.21(-0.26-0.97) |
| Equatorial Guinea | 4.42(4.04-4.83) | 3.19(2.91-3.51) | 1.41(0.20-4.85) | 1.21(0.12-4.47) | 0.86(0.74-1.01) | 0.22(0.15-0.29) | -0.17(-0.56-0.95) | -0.18(-0.57-0.95) |
| Eritrea | 1.67(1.54-1.87) | 1.46(1.34-1.63) | 0.97(0.15-2.73) | 1.18(0.30-3.14) | 0.12(0.07-0.19) | -0.01(-0.06-0.04) | -0.13(-0.45-0.51) | -0.09(-0.45-0.59) |
| Estonia | 0.25(0.19-0.33) | 0.10(0.04-0.19) | -0.82(-0.85--0.78) | -0.83(-0.86--0.80) | 0.21(0.14-0.27) | 0.14(0.09-0.20) | -0.85(-0.88--0.81) | -0.89(-0.91--0.87) |
| Eswatini | 0.91(0.82-1.03) | 0.69(0.61-0.79) | 0.74(0.23-1.54) | 0.67(0.16-1.53) | -0.04(-0.08-0.02) | -0.15(-0.19--0.10) | -0.11(-0.37-0.29) | -0.18(-0.42-0.26) |
| Ethiopia | 1.45(1.34-1.57) | 1.33(1.23-1.46) | 0.74(-0.16-2.80) | 0.87(-0.13-3.09) | 0.11(0.06-0.16) | 0.01(-0.03-0.05) | -0.20(-0.60-0.59) | -0.16(-0.60-0.63) |
| Fiji | 0.72(0.62-0.83) | 0.44(0.34-0.53) | 0.51(0.05-1.27) | 0.80(0.22-1.71) | 0.03(-0.00-0.08) | -0.06(-0.09--0.03) | 0.03(-0.27-0.48) | 0.06(-0.27-0.55) |
| Finland | 1.27(1.11-1.45) | 1.52(1.35-1.70) | 2.59(2.07-3.21) | 3.72(2.97-4.56) | 0.35(0.27-0.42) | 0.48(0.41-0.55) | 0.98(0.71-1.31) | 1.33(0.98-1.73) |
| France | 1.06(0.95-1.18) | 1.22(1.09-1.35) | 1.89(1.59-2.22) | 2.56(2.09-3.00) | 0.28(0.22-0.34) | 0.36(0.30-0.42) | 0.70(0.52-0.89) | 0.87(0.65-1.09) |
| Gabon | 1.35(1.21-1.51) | 1.15(1.04-1.30) | 0.50(-0.10-1.54) | 0.36(-0.21-1.31) | 0.19(0.13-0.26) | 0.08(0.03-0.14) | -0.20(-0.53-0.31) | -0.21(-0.55-0.32) |
| Gambia | 1.55(1.42-1.69) | 1.34(1.22-1.46) | 1.28(0.43-2.66) | 1.31(0.44-2.58) | -0.10(-0.15--0.05) | -0.20(-0.24--0.16) | -0.19(-0.49-0.21) | -0.21(-0.51-0.19) |
| Georgia | -0.15(-0.20--0.08) | -0.04(-0.10-0.03) | -0.15(-0.33-0.07) | -0.01(-0.25-0.33) | -0.02(-0.08-0.04) | 0.06(0.01-0.12) | -0.08(-0.27-0.15) | -0.06(-0.29-0.25) |
| Germany | 0.75(0.66-0.85) | 0.81(0.72-0.90) | 1.25(0.97-1.60) | 1.94(1.50-2.48) | 0.21(0.16-0.26) | 0.27(0.21-0.32) | 0.45(0.28-0.65) | 0.72(0.48-1.01) |
| Ghana | 1.87(1.71-2.05) | 1.54(1.40-1.69) | 1.66(0.69-3.18) | 1.77(0.83-3.33) | 0.06(-0.00-0.12) | -0.08(-0.13--0.02) | 0.01(-0.32-0.51) | 0.02(-0.32-0.51) |
| Greece | 1.43(1.23-1.66) | 1.79(1.56-2.01) | 5.19(4.58-5.76) | 7.88(6.97-8.86) | 0.66(0.55-0.79) | 0.92(0.80-1.05) | 2.89(2.55-3.21) | 3.68(3.26-4.15) |
| Greenland | 1.04(0.91-1.17) | 0.94(0.81-1.07) | 0.56(0.03-1.15) | 0.65(0.00-1.40) | 0.10(0.05-0.14) | 0.07(0.03-0.11) | -0.21(-0.44-0.11) | -0.24(-0.52-0.11) |
| Grenada | 1.64(1.49-1.82) | 1.31(1.17-1.46) | 2.39(1.70-3.19) | 2.22(1.63-3.00) | 0.53(0.44-0.64) | 0.45(0.38-0.53) | 1.21(0.75-1.74) | 1.47(1.01-2.07) |
| Guam | 0.54(0.46-0.62) | 0.30(0.23-0.37) | -0.03(-0.27-0.33) | 0.14(-0.19-0.61) | -0.14(-0.17--0.11) | -0.20(-0.22--0.18) | -0.43(-0.57--0.22) | -0.55(-0.68--0.36) |
| Guatemala | 2.80(2.62-3.00) | 2.73(2.58-2.91) | 2.58(1.64-3.67) | 3.55(2.47-4.97) | 0.27(0.22-0.32) | 0.25(0.21-0.29) | 0.29(-0.01-0.65) | 0.33(0.06-0.68) |
| Guinea | 0.69(0.59-0.81) | 0.58(0.48-0.70) | 0.47(-0.04-1.35) | 0.36(-0.13-1.18) | -0.12(-0.16--0.07) | -0.22(-0.26--0.17) | -0.19(-0.48-0.29) | -0.20(-0.50-0.27) |
| Guinea-Bissau | 0.84(0.74-0.97) | 0.68(0.57-0.81) | 0.39(-0.16-1.30) | 0.26(-0.23-1.01) | -0.14(-0.18--0.10) | -0.26(-0.30--0.21) | -0.30(-0.57-0.10) | -0.31(-0.58-0.08) |
| Guyana | 1.31(1.15-1.52) | 0.95(0.82-1.10) | 1.20(0.57-1.93) | 1.02(0.46-1.72) | 0.49(0.39-0.60) | 0.29(0.23-0.35) | 0.37(-0.01-0.83) | 0.20(-0.12-0.60) |
| Haiti | 1.89(1.73-2.08) | 1.89(1.78-2.04) | 1.77(0.63-3.85) | 1.83(0.66-3.92) | 0.24(0.18-0.32) | 0.26(0.21-0.32) | 0.26(-0.23-1.15) | 0.26(-0.24-1.14) |
| Honduras | 3.78(3.53-4.08) | 3.71(3.48-3.98) | 2.61(1.23-4.70) | 3.39(1.69-6.12) | 0.58(0.50-0.67) | 0.57(0.50-0.65) | 0.40(-0.11-1.19) | 0.54(-0.10-1.61) |
| Hungary | 0.36(0.29-0.43) | 0.25(0.19-0.32) | 0.52(0.31-0.72) | 0.83(0.58-1.07) | 0.16(0.11-0.21) | 0.10(0.07-0.14) | 0.20(0.03-0.36) | 0.33(0.15-0.51) |
| Iceland | 1.69(1.54-1.86) | 1.80(1.66-1.96) | 4.11(3.52-4.79) | 5.28(4.45-6.16) | 0.36(0.29-0.44) | 0.43(0.36-0.50) | 1.57(1.29-1.91) | 1.99(1.60-2.41) |
| India | 1.68(1.56-1.81) | 1.71(1.57-1.85) | 1.86(1.21-3.28) | 2.27(1.51-3.92) | 0.07(0.03-0.12) | 0.08(0.04-0.13) | 0.12(-0.12-0.65) | 0.18(-0.08-0.76) |
| Indonesia | 1.93(1.77-2.13) | 1.83(1.68-2.01) | 1.61(0.94-2.66) | 1.74(0.83-3.85) | 0.22(0.16-0.29) | 0.22(0.17-0.28) | 0.17(-0.09-0.60) | 0.19(-0.15-1.12) |
| Iran (Islamic Republic of) | 2.17(2.01-2.36) | 2.04(1.82-2.32) | 2.62(1.91-3.72) | 7.04(2.20-13.81) | 0.12(0.07-0.17) | 0.16(0.11-0.22) | 0.30(0.04-0.70) | 1.33(0.06-3.21) |
| Iraq | 3.33(3.04-3.71) | 2.73(2.50-3.06) | 2.27(1.21-4.29) | 2.18(1.09-4.66) | 0.39(0.31-0.51) | 0.24(0.18-0.33) | 0.16(-0.19-0.88) | 0.23(-0.16-1.08) |
| Ireland | 2.73(2.53-2.94) | 2.83(2.66-3.02) | 2.87(2.44-3.32) | 3.67(3.06-4.35) | 0.94(0.84-1.06) | 0.96(0.87-1.06) | 1.01(0.80-1.23) | 1.31(1.03-1.64) |
| Israel | 1.89(1.74-2.05) | 1.85(1.68-2.01) | 2.55(2.15-2.99) | 2.97(2.44-3.54) | 0.19(0.13-0.25) | 0.16(0.10-0.21) | 0.42(0.27-0.59) | 0.45(0.27-0.64) |
| Italy | 0.97(0.79-1.20) | 1.45(1.24-1.70) | 6.68(5.63-8.02) | 16.18(14.67-17.23) | 0.29(0.18-0.41) | 0.59(0.48-0.73) | 3.50(2.90-4.25) | 8.04(7.43-8.52) |
| Jamaica | 1.37(1.23-1.56) | 1.35(1.21-1.54) | 3.03(2.15-4.10) | 3.61(2.55-4.98) | 0.35(0.27-0.47) | 0.38(0.30-0.48) | 1.44(0.90-2.09) | 1.70(1.08-2.51) |
| Japan | 1.11(0.92-1.31) | 1.11(0.86-1.39) | 2.25(1.95-2.46) | 4.11(3.50-4.48) | 0.10(0.04-0.16) | 0.27(0.19-0.35) | 0.38(0.31-0.44) | 0.72(0.59-0.81) |
| Jordan | 5.88(5.57-6.23) | 5.21(4.89-5.55) | 3.48(1.62-6.78) | 3.82(1.67-7.78) | 0.25(0.19-0.31) | 0.15(0.10-0.20) | -0.09(-0.47-0.54) | -0.07(-0.48-0.68) |
| Kazakhstan | 0.99(0.89-1.14) | 0.89(0.78-1.02) | 0.34(0.00-0.73) | 0.34(-0.04-0.83) | 0.44(0.36-0.53) | 0.38(0.31-0.46) | -0.03(-0.28-0.26) | -0.04(-0.31-0.31) |
| Kenya | 1.84(1.74-1.97) | 1.84(1.73-1.97) | 2.23(1.23-3.44) | 2.32(1.35-3.59) | -0.02(-0.05-0.03) | 0.01(-0.02-0.05) | 0.23(-0.10-0.62) | 0.25(-0.09-0.71) |
| Kiribati | 0.99(0.88-1.09) | 0.81(0.73-0.89) | 0.70(0.17-1.63) | 0.78(0.18-1.65) | 0.04(-0.00-0.09) | -0.01(-0.04-0.02) | -0.01(-0.32-0.43) | 0.01(-0.34-0.44) |
| Kuwait | 4.74(4.44-5.07) | 4.63(4.25-5.06) | 2.16(1.65-2.75) | 2.76(2.12-3.49) | 0.28(0.23-0.35) | 0.34(0.29-0.39) | -0.24(-0.37--0.10) | -0.19(-0.33--0.03) |
| Kyrgyzstan | 0.47(0.39-0.55) | 0.24(0.17-0.31) | -0.57(-0.65--0.48) | -0.67(-0.74--0.56) | -0.19(-0.22--0.15) | -0.31(-0.35--0.28) | -0.74(-0.79--0.68) | -0.79(-0.83--0.71) |
| Lao People's Democratic Republic | 2.11(1.92-2.32) | 1.69(1.54-1.86) | 1.24(0.35-3.02) | 1.17(0.00-3.70) | 0.39(0.30-0.48) | 0.12(0.07-0.18) | 0.01(-0.35-0.69) | -0.04(-0.52-0.97) |
| Latvia | -0.41(-0.44--0.38) | -0.53(-0.55--0.51) | -0.89(-0.91--0.87) | -0.90(-0.92--0.88) | -0.30(-0.34--0.26) | -0.39(-0.41--0.36) | -0.89(-0.91--0.86) | -0.92(-0.93--0.90) |
| Lebanon | 2.35(2.15-2.57) | 2.02(1.81-2.23) | 1.51(0.39-4.10) | 2.38(0.86-6.50) | 0.32(0.24-0.40) | 0.16(0.11-0.23) | -0.10(-0.49-0.88) | -0.01(-0.44-1.20) |
| Lesotho | 0.38(0.30-0.47) | 0.24(0.16-0.33) | 0.37(-0.12-1.07) | 0.22(-0.25-0.94) | 0.02(-0.03-0.09) | -0.08(-0.13--0.02) | 0.05(-0.33-0.60) | -0.03(-0.41-0.54) |
| Liberia | 1.11(0.96-1.25) | 0.93(0.78-1.08) | 0.58(-0.03-1.48) | 0.38(-0.13-1.15) | -0.06(-0.10--0.02) | -0.14(-0.17--0.10) | -0.22(-0.49-0.20) | -0.25(-0.51-0.15) |
| Libya | 2.15(1.97-2.37) | 1.93(1.75-2.18) | 5.83(2.29-12.30) | 16.25(8.57-119.75) | 0.08(0.02-0.15) | 0.05(-0.01-0.12) | 1.58(0.14-4.11) | 5.72(2.67-48.10) |
| Lithuania | -0.07(-0.12--0.01) | -0.25(-0.29--0.20) | -0.81(-0.85--0.77) | -0.85(-0.87--0.82) | -0.04(-0.08-0.02) | -0.11(-0.14--0.07) | -0.83(-0.87--0.79) | -0.89(-0.91--0.87) |
| Luxembourg | 1.59(1.47-1.72) | 1.66(1.55-1.79) | 3.11(2.63-3.58) | 4.02(3.40-4.63) | 0.36(0.30-0.43) | 0.38(0.32-0.44) | 1.12(0.87-1.36) | 1.46(1.16-1.76) |
| Madagascar | 1.74(1.62-1.87) | 1.59(1.47-1.73) | 1.41(0.41-2.84) | 1.19(0.35-2.30) | 0.13(0.08-0.19) | 0.07(0.03-0.12) | 0.06(-0.34-0.58) | 0.06(-0.32-0.58) |
| Malawi | 1.22(1.11-1.35) | 1.11(1.01-1.23) | 1.15(0.26-2.59) | 1.23(0.26-2.59) | 0.10(0.05-0.17) | -0.00(-0.05-0.05) | 0.14(-0.31-0.72) | 0.14(-0.30-0.72) |
| Malaysia | 3.52(3.23-3.86) | 2.75(2.52-2.99) | 2.32(1.40-3.64) | 2.45(1.42-3.89) | 0.51(0.44-0.60) | 0.30(0.24-0.36) | 0.16(-0.17-0.60) | 0.17(-0.18-0.68) |
| Maldives | 4.51(4.19-4.86) | 4.31(3.98-4.68) | 1.14(0.06-4.29) | 1.32(0.22-4.40) | 0.52(0.42-0.62) | 0.30(0.23-0.37) | -0.41(-0.67-0.25) | -0.43(-0.68-0.22) |
| Mali | 1.47(1.31-1.64) | 1.25(1.09-1.41) | 1.08(0.22-2.35) | 1.00(0.15-2.23) | 0.05(-0.02-0.13) | -0.11(-0.17--0.04) | -0.12(-0.49-0.38) | -0.13(-0.49-0.39) |
| Malta | 2.40(2.18-2.64) | 2.22(2.04-2.42) | 4.13(3.51-4.78) | 5.25(4.41-6.22) | 0.59(0.52-0.68) | 0.56(0.50-0.62) | 1.25(0.98-1.52) | 1.36(1.06-1.73) |
| Marshall Islands | 1.16(1.04-1.27) | 0.83(0.74-0.93) | 0.47(0.01-1.03) | 0.57(0.04-1.17) | 0.08(0.04-0.13) | 0.02(-0.02-0.06) | -0.07(-0.35-0.26) | -0.07(-0.36-0.27) |
| Mauritania | 1.16(1.04-1.30) | 0.86(0.76-0.97) | 0.39(-0.20-1.41) | 0.37(-0.18-1.26) | -0.03(-0.08-0.02) | -0.20(-0.24--0.15) | -0.36(-0.62-0.06) | -0.37(-0.62-0.01) |
| Mauritius | 4.41(4.09-4.72) | 3.92(3.66-4.17) | 5.95(5.36-6.54) | 7.62(6.85-8.41) | 1.14(1.01-1.24) | 0.96(0.87-1.05) | 1.95(1.71-2.20) | 2.22(1.93-2.52) |
| Mexico | 1.95(1.76-2.17) | 2.43(2.20-2.67) | 3.81(3.31-4.34) | 4.79(4.18-5.41) | 0.06(-0.00-0.12) | 0.29(0.21-0.37) | 0.77(0.59-0.95) | 0.86(0.67-1.06) |
| Micronesia (Federated States of) | 0.59(0.50-0.67) | 0.38(0.31-0.45) | -0.09(-0.42-0.42) | -0.01(-0.39-0.55) | 0.12(0.07-0.16) | 0.02(-0.01-0.06) | -0.20(-0.48-0.22) | -0.17(-0.49-0.26) |
| Monaco | 0.52(0.45-0.60) | 0.55(0.46-0.63) | 0.52(0.10-1.16) | 0.61(0.13-1.41) | 0.10(0.05-0.16) | 0.11(0.06-0.16) | 0.09(-0.20-0.53) | 0.13(-0.20-0.67) |
| Mongolia | 1.71(1.59-1.85) | 1.05(0.94-1.17) | 0.07(-0.42-0.95) | 0.13(-0.37-1.05) | 0.19(0.14-0.23) | -0.04(-0.07-0.00) | -0.43(-0.68--0.01) | -0.41(-0.66-0.06) |
| Montenegro | 0.21(0.15-0.28) | 0.05(-0.01-0.12) | 0.27(-0.03-0.68) | 0.52(-0.07-1.51) | -0.08(-0.12--0.03) | -0.09(-0.13--0.04) | -0.09(-0.30-0.19) | -0.01(-0.39-0.63) |
| Morocco | 2.20(2.00-2.50) | 1.72(1.54-1.95) | 4.19(2.08-7.21) | 11.56(8.15-99.37) | 0.40(0.32-0.50) | 0.22(0.16-0.30) | 1.39(0.36-2.76) | 4.55(3.09-43.97) |
| Mozambique | 1.23(1.11-1.37) | 1.05(0.93-1.16) | 1.09(0.18-2.53) | 1.11(0.34-2.35) | 0.12(0.06-0.19) | -0.05(-0.10-0.00) | 0.12(-0.27-0.69) | 0.11(-0.31-0.70) |
| Myanmar | 2.59(2.35-2.87) | 2.04(1.85-2.28) | 1.35(0.48-3.24) | 1.54(0.23-4.42) | 0.76(0.65-0.89) | 0.42(0.35-0.51) | 0.20(-0.20-1.07) | 0.18(-0.40-1.47) |
| Namibia | 1.41(1.29-1.55) | 1.12(1.00-1.25) | 0.99(0.31-2.11) | 1.00(0.32-2.15) | 0.11(0.06-0.18) | -0.05(-0.10-0.00) | -0.07(-0.37-0.39) | -0.10(-0.39-0.33) |
| Nauru | 0.20(0.14-0.25) | 0.17(0.12-0.21) | 0.03(-0.27-0.42) | 0.10(-0.23-0.57) | -0.01(-0.05-0.03) | -0.05(-0.08--0.02) | -0.08(-0.34-0.26) | -0.03(-0.33-0.55) |
| Nepal | 2.83(2.60-3.09) | 2.22(2.05-2.41) | 1.73(0.71-3.68) | 2.00(0.93-3.98) | 0.59(0.50-0.69) | 0.30(0.23-0.38) | 0.12(-0.28-0.81) | 0.14(-0.24-0.86) |
| Netherlands | 1.99(1.76-2.25) | 2.37(2.08-2.60) | 5.07(4.42-5.73) | 7.83(6.81-8.87) | 0.73(0.62-0.86) | 0.98(0.85-1.11) | 2.34(2.00-2.68) | 3.61(3.11-4.14) |
| New Zealand | 1.27(1.07-1.49) | 1.82(1.58-2.09) | 3.29(2.90-3.70) | 4.58(3.97-5.18) | 0.06(-0.03-0.16) | 0.30(0.20-0.42) | 0.99(0.82-1.15) | 1.43(1.20-1.68) |
| Nicaragua | 3.60(3.24-4.04) | 3.78(3.40-4.20) | 2.51(1.36-3.99) | 3.02(1.47-5.12) | 0.56(0.45-0.70) | 0.70(0.58-0.82) | 0.31(-0.13-0.86) | 0.31(-0.19-1.02) |
| Niger | 1.53(1.40-1.66) | 1.32(1.20-1.45) | 1.05(0.29-2.49) | 1.13(0.32-2.43) | -0.16(-0.20--0.12) | -0.26(-0.30--0.22) | -0.30(-0.56-0.11) | -0.28(-0.56-0.14) |
| Nigeria | 1.02(0.92-1.13) | 0.94(0.85-1.04) | 0.80(0.20-1.82) | 0.66(0.11-1.63) | -0.12(-0.16--0.08) | -0.17(-0.21--0.14) | -0.17(-0.45-0.28) | -0.17(-0.44-0.28) |
| Niue | 0.04(-0.00-0.08) | -0.11(-0.14--0.07) | -0.01(-0.31-0.45) | -0.16(-0.45-0.17) | 0.08(0.03-0.12) | -0.02(-0.06-0.01) | 0.33(-0.09-0.99) | 0.06(-0.29-0.49) |
| North Macedonia | 0.47(0.40-0.56) | 0.22(0.15-0.31) | 0.65(0.10-1.77) | 1.01(0.09-2.74) | -0.03(-0.07-0.01) | -0.09(-0.13--0.05) | 0.03(-0.32-0.70) | 0.18(-0.34-1.17) |
| Northern Mariana Islands | 0.76(0.63-0.92) | 0.52(0.35-0.72) | -0.10(-0.53-0.55) | 0.18(-0.41-1.09) | -0.03(-0.08-0.02) | -0.02(-0.07-0.03) | -0.37(-0.64-0.04) | -0.37(-0.66-0.08) |
| Norway | 0.59(0.51-0.66) | 0.94(0.83-1.06) | 1.39(1.27-1.52) | 1.84(1.68-1.99) | 0.07(0.02-0.12) | 0.23(0.16-0.30) | 0.62(0.54-0.70) | 0.91(0.81-1.01) |
| Oman | 3.88(3.64-4.18) | 3.65(3.35-4.00) | 2.79(0.90-6.20) | 2.73(0.61-7.81) | 0.61(0.52-0.71) | 0.51(0.44-0.60) | 0.36(-0.34-1.71) | 0.46(-0.35-2.30) |
| Pakistan | 0.71(0.64-0.79) | 0.95(0.88-1.04) | 1.17(0.56-2.19) | 1.12(0.45-2.20) | -0.25(-0.28--0.22) | -0.10(-0.14--0.06) | 0.01(-0.28-0.50) | 0.07(-0.26-0.67) |
| Palau | 0.91(0.80-1.02) | 0.53(0.42-0.65) | 0.38(-0.10-1.04) | 0.57(-0.02-1.35) | 0.02(-0.02-0.06) | -0.05(-0.08--0.02) | -0.03(-0.36-0.39) | -0.05(-0.38-0.38) |
| Palestine | 3.27(3.03-3.54) | 3.16(2.93-3.40) | 1.63(0.72-3.25) | 1.50(0.58-3.31) | 0.39(0.30-0.47) | 0.28(0.21-0.35) | -0.04(-0.36-0.55) | -0.03(-0.38-0.62) |
| Panama | 4.26(3.89-4.71) | 4.11(3.76-4.50) | 4.86(3.71-6.10) | 6.88(5.21-8.73) | 0.89(0.76-1.03) | 0.89(0.78-1.00) | 1.19(0.77-1.65) | 1.58(1.03-2.17) |
| Papua New Guinea | 2.37(2.20-2.55) | 2.20(2.04-2.37) | 1.91(0.69-3.71) | 1.99(0.75-3.87) | 0.19(0.14-0.25) | 0.15(0.10-0.21) | 0.09(-0.33-0.70) | 0.09(-0.37-0.72) |
| Paraguay | 2.51(2.27-2.78) | 2.55(2.34-2.78) | 2.57(1.38-4.14) | 2.89(1.49-4.89) | 0.40(0.32-0.50) | 0.43(0.36-0.52) | 0.46(-0.03-1.13) | 0.50(-0.04-1.30) |
| Peru | 3.96(3.75-4.18) | 3.82(3.59-4.05) | 2.28(1.06-4.09) | 3.11(1.50-5.75) | 0.80(0.72-0.88) | 0.69(0.60-0.78) | 0.24(-0.22-0.91) | 0.37(-0.17-1.24) |
| Philippines | 1.14(1.06-1.25) | 1.01(0.93-1.10) | 1.58(1.24-2.07) | 2.12(1.43-2.92) | -0.22(-0.25--0.18) | -0.24(-0.27--0.21) | -0.04(-0.17-0.14) | -0.00(-0.23-0.29) |
| Poland | 0.35(0.26-0.47) | 0.25(0.17-0.36) | 0.68(0.56-0.80) | 1.12(0.94-1.31) | -0.00(-0.06-0.07) | -0.01(-0.06-0.05) | 0.12(0.05-0.20) | 0.27(0.17-0.38) |
| Portugal | 2.00(1.75-2.28) | 2.00(1.76-2.28) | 3.82(3.33-4.38) | 5.77(4.85-6.65) | 0.83(0.71-0.99) | 0.83(0.71-0.97) | 1.66(1.41-1.92) | 2.08(1.72-2.45) |
| Puerto Rico | 1.96(1.76-2.18) | 1.82(1.65-2.00) | 2.19(1.64-2.77) | 3.11(2.34-3.88) | 0.72(0.63-0.83) | 0.61(0.54-0.68) | 0.82(0.51-1.15) | 0.80(0.47-1.12) |
| Qatar | 8.69(8.16-9.21) | 7.71(7.25-8.22) | 6.27(3.27-10.26) | 5.57(2.23-10.54) | 0.12(0.07-0.17) | -0.01(-0.05-0.04) | -0.19(-0.54-0.27) | -0.24(-0.58-0.23) |
| Republic of Korea | 3.49(3.12-3.87) | 3.15(2.73-3.56) | 2.49(0.82-4.86) | 3.34(0.83-7.86) | 0.74(0.65-0.84) | 0.79(0.69-0.88) | 0.10(-0.44-0.89) | 0.09(-0.55-1.22) |
| Republic of Moldova | 0.03(-0.02-0.07) | -0.15(-0.19--0.10) | -0.74(-0.80--0.68) | -0.89(-0.91--0.87) | -0.07(-0.11--0.02) | -0.12(-0.16--0.09) | -0.79(-0.84--0.74) | -0.93(-0.94--0.92) |
| Romania | -0.12(-0.15--0.08) | -0.25(-0.29--0.21) | -0.43(-0.52--0.32) | -0.37(-0.49--0.24) | -0.22(-0.24--0.18) | -0.32(-0.34--0.30) | -0.51(-0.59--0.42) | -0.55(-0.63--0.45) |
| Russian Federation | -0.21(-0.24--0.18) | -0.31(-0.34--0.27) | -0.40(-0.47--0.30) | -0.42(-0.49--0.33) | -0.32(-0.34--0.28) | -0.35(-0.37--0.32) | -0.52(-0.58--0.44) | -0.59(-0.64--0.53) |
| Rwanda | 1.73(1.61-1.87) | 1.38(1.28-1.50) | 0.54(-0.35-2.49) | 0.65(-0.30-2.54) | 0.20(0.15-0.26) | 0.00(-0.04-0.05) | -0.25(-0.65-0.49) | -0.23(-0.65-0.50) |
| Saint Kitts and Nevis | 2.35(2.15-2.57) | 2.07(1.84-2.31) | 1.93(1.32-2.69) | 1.73(1.18-2.39) | 0.68(0.59-0.78) | 0.54(0.48-0.61) | 0.66(0.33-1.06) | 0.81(0.45-1.24) |
| Saint Lucia | 3.35(3.06-3.72) | 2.87(2.61-3.14) | 3.64(2.71-4.72) | 3.90(2.96-5.02) | 0.68(0.57-0.79) | 0.51(0.44-0.59) | 0.86(0.50-1.29) | 0.71(0.39-1.09) |
| Saint Vincent and the Grenadines | 1.84(1.60-2.14) | 1.77(1.51-2.08) | 9.30(7.66-11.26) | 11.39(9.50-13.49) | 0.50(0.39-0.65) | 0.56(0.45-0.69) | 4.58(3.69-5.62) | 5.38(4.45-6.42) |
| Samoa | 0.77(0.68-0.86) | 0.50(0.43-0.57) | 0.20(-0.17-0.62) | 0.35(-0.08-0.85) | 0.13(0.07-0.18) | 0.01(-0.03-0.05) | -0.12(-0.38-0.19) | -0.10(-0.38-0.22) |
| San Marino | 0.71(0.62-0.80) | 0.76(0.66-0.87) | 0.44(-0.10-1.33) | 0.54(-0.11-1.77) | -0.10(-0.14--0.05) | -0.08(-0.11--0.05) | -0.30(-0.56-0.12) | -0.36(-0.64-0.15) |
| Sao Tome and Principe | 1.46(1.30-1.67) | 1.22(1.06-1.42) | 0.65(0.11-1.48) | 0.46(0.05-1.09) | 0.27(0.20-0.35) | 0.08(0.02-0.14) | -0.09(-0.35-0.30) | -0.11(-0.36-0.23) |
| Saudi Arabia | 5.63(5.26-6.04) | 5.16(4.76-5.57) | 3.31(1.61-7.34) | 2.54(1.13-5.85) | 0.84(0.75-0.94) | 0.60(0.53-0.68) | 0.24(-0.23-1.23) | 0.19(-0.28-1.13) |
| Senegal | 1.34(1.21-1.47) | 1.06(0.95-1.17) | 0.77(0.03-1.91) | 0.78(0.06-1.86) | -0.06(-0.11--0.01) | -0.19(-0.24--0.15) | -0.25(-0.55-0.18) | -0.26(-0.55-0.17) |
| Serbia | 0.31(0.24-0.39) | 0.19(0.12-0.27) | 0.20(-0.17-0.69) | 0.40(-0.07-1.16) | 0.10(0.05-0.16) | 0.06(0.01-0.11) | -0.14(-0.41-0.22) | -0.19(-0.47-0.24) |
| Seychelles | 1.69(1.55-1.88) | 1.47(1.32-1.65) | 0.83(0.44-1.48) | 0.55(-0.05-1.19) | 0.27(0.20-0.35) | 0.16(0.11-0.23) | -0.09(-0.28-0.20) | -0.15(-0.46-0.22) |
| Sierra Leone | 0.83(0.71-0.95) | 0.70(0.59-0.81) | 0.50(-0.08-1.37) | 0.33(-0.19-1.13) | -0.13(-0.18--0.08) | -0.22(-0.26--0.18) | -0.24(-0.53-0.20) | -0.27(-0.56-0.16) |
| Singapore | 3.38(3.02-3.77) | 2.87(2.52-3.27) | 2.86(2.49-3.25) | 3.73(3.19-4.28) | 0.36(0.28-0.45) | 0.34(0.25-0.43) | 0.05(-0.04-0.15) | 0.11(-0.01-0.25) |
| Slovakia | 0.76(0.64-0.90) | 0.58(0.46-0.73) | 0.49(-0.15-1.53) | 0.62(-0.18-2.06) | 0.23(0.16-0.32) | 0.19(0.12-0.29) | -0.01(-0.42-0.68) | 0.01(-0.49-0.92) |
| Slovenia | 1.21(1.04-1.40) | 1.41(1.18-1.63) | 1.09(0.77-1.42) | 1.99(1.47-2.53) | 0.46(0.38-0.54) | 0.62(0.53-0.72) | 0.19(0.01-0.37) | 0.52(0.26-0.78) |
| Solomon Islands | 1.72(1.58-1.87) | 1.45(1.33-1.60) | 1.51(0.58-3.03) | 1.66(0.61-3.31) | 0.07(0.02-0.12) | -0.00(-0.04-0.05) | 0.07(-0.30-0.67) | 0.06(-0.32-0.62) |
| Somalia | 1.38(1.27-1.50) | 1.40(1.29-1.52) | 1.15(0.41-2.48) | 1.17(0.46-2.45) | -0.11(-0.15--0.07) | -0.13(-0.16--0.09) | -0.17(-0.43-0.24) | -0.20(-0.44-0.22) |
| South Africa | 0.95(0.88-1.01) | 0.89(0.82-0.97) | 0.93(0.46-1.63) | 1.03(0.40-1.82) | -0.13(-0.15--0.09) | -0.13(-0.16--0.09) | -0.08(-0.32-0.22) | -0.07(-0.38-0.35) |
| South Sudan | 0.42(0.33-0.51) | 0.37(0.29-0.46) | 0.35(-0.13-1.19) | 0.25(-0.23-0.96) | -0.15(-0.19--0.11) | -0.14(-0.18--0.09) | -0.11(-0.45-0.37) | -0.12(-0.47-0.34) |
| Spain | 1.48(1.35-1.60) | 1.65(1.51-1.82) | 2.73(2.31-3.09) | 4.42(3.71-5.01) | 0.43(0.37-0.49) | 0.52(0.46-0.60) | 0.93(0.75-1.09) | 1.41(1.14-1.66) |
| Sri Lanka | 2.81(2.48-3.13) | 2.21(1.95-2.47) | 0.96(0.15-2.19) | 1.29(0.32-2.84) | 0.63(0.54-0.73) | 0.43(0.37-0.51) | -0.15(-0.49-0.37) | -0.13(-0.49-0.48) |
| Sudan | 1.97(1.81-2.20) | 1.71(1.55-1.91) | 3.91(2.01-7.11) | 7.81(4.40-77.85) | 0.32(0.24-0.42) | 0.14(0.08-0.21) | 1.29(0.31-2.74) | 3.29(1.76-39.74) |
| Suriname | 2.24(2.02-2.49) | 2.00(1.83-2.20) | 1.96(0.87-3.32) | 1.99(0.79-3.68) | 0.40(0.32-0.49) | 0.34(0.28-0.41) | 0.31(-0.17-0.94) | 0.20(-0.28-0.94) |
| Sweden | 0.58(0.48-0.69) | 1.04(0.87-1.22) | 1.43(1.15-1.77) | 1.98(1.61-2.42) | 0.09(0.03-0.15) | 0.33(0.23-0.43) | 0.63(0.45-0.84) | 0.92(0.68-1.19) |
| Switzerland | 0.88(0.79-0.97) | 1.01(0.92-1.11) | 1.43(1.13-1.76) | 1.92(1.49-2.35) | 0.11(0.06-0.16) | 0.18(0.14-0.23) | 0.39(0.22-0.57) | 0.55(0.34-0.78) |
| Syrian Arab Republic | 2.26(2.02-2.59) | 1.76(1.53-2.05) | 1.94(0.93-3.46) | 2.16(0.88-4.06) | 0.38(0.31-0.48) | 0.26(0.20-0.33) | 0.36(-0.11-1.02) | 0.43(-0.14-1.22) |
| Taiwan (Province of China) | 4.44(3.91-5.04) | 3.99(3.54-4.48) | 6.66(5.74-7.64) | 9.91(8.41-11.30) | 1.19(1.01-1.40) | 1.13(0.99-1.28) | 1.91(1.59-2.24) | 2.40(1.96-2.82) |
| Tajikistan | 1.40(1.29-1.50) | 1.45(1.34-1.57) | 0.50(-0.23-2.21) | 0.23(-0.45-2.05) | 0.10(0.05-0.15) | 0.20(0.15-0.26) | -0.27(-0.64-0.60) | -0.32(-0.70-0.80) |
| Thailand | 1.96(1.77-2.21) | 1.34(1.15-1.55) | 1.21(0.55-2.19) | 1.50(0.56-2.97) | 0.12(0.07-0.18) | -0.05(-0.09--0.01) | -0.14(-0.39-0.24) | -0.21(-0.50-0.24) |
| Timor-Leste | 2.41(2.10-2.77) | 1.89(1.65-2.14) | 1.79(0.81-3.22) | 2.32(0.66-4.76) | 0.28(0.21-0.36) | 0.12(0.06-0.18) | 0.09(-0.21-0.52) | 0.04(-0.40-0.82) |
| Togo | 1.79(1.66-1.94) | 1.45(1.32-1.58) | 1.41(0.51-2.73) | 1.37(0.47-2.56) | -0.14(-0.19--0.10) | -0.24(-0.28--0.20) | -0.20(-0.49-0.18) | -0.22(-0.52-0.14) |
| Tokelau | 0.20(0.14-0.25) | -0.01(-0.05-0.04) | 0.36(-0.14-1.24) | 0.21(-0.27-0.98) | 0.12(0.07-0.16) | -0.03(-0.07-0.00) | 0.42(-0.09-1.30) | 0.13(-0.31-0.83) |
| Tonga | 0.52(0.44-0.60) | 0.35(0.29-0.43) | 0.23(-0.17-0.75) | 0.39(-0.09-0.95) | 0.14(0.08-0.20) | 0.04(-0.00-0.09) | -0.00(-0.32-0.39) | -0.00(-0.36-0.38) |
| Trinidad and Tobago | 1.88(1.71-2.04) | 1.57(1.43-1.72) | 2.51(1.76-3.50) | 2.78(1.97-3.77) | 0.36(0.30-0.42) | 0.22(0.18-0.26) | 0.68(0.32-1.14) | 0.56(0.22-0.97) |
| Tunisia | 2.32(2.13-2.58) | 1.83(1.66-2.08) | 4.06(2.04-7.80) | 12.96(7.74-118.48) | 0.35(0.28-0.44) | 0.18(0.12-0.24) | 1.13(0.26-2.68) | 4.02(2.26-45.83) |
| Turkey | 2.72(2.47-3.06) | 2.11(1.91-2.37) | 1.77(0.62-4.03) | 2.25(0.81-5.38) | 0.52(0.43-0.64) | 0.31(0.24-0.40) | 0.13(-0.34-1.05) | 0.16(-0.33-1.29) |
| Turkmenistan | 0.49(0.42-0.57) | 0.14(0.07-0.20) | 0.11(-0.14-0.47) | -0.09(-0.30-0.23) | -0.30(-0.34--0.26) | -0.46(-0.49--0.43) | -0.47(-0.59--0.31) | -0.59(-0.69--0.46) |
| Tuvalu | 0.75(0.67-0.83) | 0.55(0.47-0.61) | 0.14(-0.25-0.89) | 0.24(-0.20-1.04) | 0.20(0.14-0.25) | 0.04(0.00-0.08) | -0.18(-0.45-0.31) | -0.17(-0.46-0.32) |
| Uganda | 1.82(1.69-1.99) | 1.53(1.41-1.67) | 1.18(0.20-2.93) | 1.07(0.16-2.62) | 0.16(0.11-0.23) | -0.02(-0.07-0.03) | -0.08(-0.46-0.55) | -0.11(-0.49-0.51) |
| Ukraine | -0.68(-0.70--0.65) | -0.70(-0.72--0.67) | -0.73(-0.80--0.66) | -0.75(-0.81--0.66) | -0.66(-0.69--0.63) | -0.67(-0.69--0.64) | -0.73(-0.79--0.65) | -0.76(-0.83--0.68) |
| United Arab Emirates | 12.01(10.87-13.30) | 10.27(8.89-11.76) | 4.43(2.48-7.53) | 4.35(2.36-7.63) | 0.19(0.13-0.27) | 0.12(0.05-0.19) | -0.15(-0.46-0.37) | -0.01(-0.37-0.63) |
| United Kingdom | 0.71(0.60-0.85) | 1.36(1.18-1.52) | 2.56(2.36-2.73) | 3.82(3.53-4.02) | 0.16(0.08-0.25) | 0.53(0.43-0.63) | 1.37(1.25-1.48) | 2.10(1.93-2.21) |
| United Republic of Tanzania | 1.88(1.73-2.04) | 1.67(1.54-1.83) | 1.27(0.20-2.87) | 1.31(0.24-2.87) | 0.18(0.12-0.24) | 0.06(0.01-0.11) | -0.02(-0.44-0.54) | -0.03(-0.45-0.54) |
| United States of America | 0.93(0.82-1.05) | 1.27(1.14-1.41) | 1.69(1.56-1.80) | 2.60(2.38-2.74) | 0.07(0.02-0.14) | 0.27(0.20-0.34) | 0.46(0.39-0.51) | 0.90(0.80-0.97) |
| United States Virgin Islands | 1.87(1.62-2.12) | 1.76(1.52-1.98) | 0.52(-0.00-1.47) | 0.81(0.13-2.13) | 0.55(0.46-0.65) | 0.49(0.42-0.56) | -0.13(-0.45-0.41) | -0.23(-0.52-0.35) |
| Uruguay | 1.08(0.94-1.25) | 1.16(1.00-1.35) | 1.72(1.48-2.01) | 2.48(2.09-2.89) | 0.51(0.41-0.62) | 0.51(0.41-0.63) | 0.87(0.72-1.05) | 1.13(0.91-1.38) |
| Uzbekistan | 0.79(0.69-0.89) | 0.58(0.50-0.67) | -0.32(-0.47--0.14) | -0.52(-0.67--0.33) | -0.24(-0.28--0.19) | -0.27(-0.31--0.23) | -0.70(-0.77--0.62) | -0.76(-0.84--0.67) |
| Vanuatu | 1.72(1.57-1.89) | 1.47(1.34-1.59) | 1.27(0.43-2.39) | 1.44(0.48-2.72) | 0.04(-0.01-0.10) | -0.04(-0.09--0.00) | -0.02(-0.37-0.45) | -0.02(-0.38-0.41) |
| Venezuela (Bolivarian Republic of) | 2.37(2.13-2.61) | 2.41(2.16-2.66) | 3.29(2.30-4.35) | 4.26(3.05-5.65) | 0.19(0.12-0.25) | 0.25(0.20-0.31) | 0.62(0.26-1.03) | 0.73(0.34-1.18) |
| Viet Nam | 3.20(2.87-3.61) | 2.60(2.32-2.92) | 1.85(1.09-3.44) | 1.69(0.55-4.29) | 0.72(0.59-0.86) | 0.43(0.35-0.54) | 0.22(-0.08-0.90) | 0.16(-0.33-1.34) |
| Yemen | 2.51(2.34-2.75) | 2.40(2.22-2.62) | 4.91(2.66-8.34) | 11.41(6.84-111.69) | 0.17(0.11-0.25) | 0.11(0.05-0.18) | 1.17(0.19-2.37) | 3.55(2.06-43.07) |
| Zambia | 2.12(1.97-2.30) | 1.83(1.69-2.00) | 1.78(0.50-4.08) | 1.75(0.54-3.70) | 0.20(0.15-0.27) | 0.03(-0.02-0.09) | 0.13(-0.34-0.86) | 0.12(-0.31-0.78) |
| Zimbabwe | 0.57(0.51-0.64) | 0.59(0.54-0.66) | 0.96(0.33-1.85) | 0.85(0.12-1.93) | -0.13(-0.16--0.09) | -0.08(-0.11--0.05) | 0.11(-0.27-0.62) | 0.06(-0.36-0.68) |

PC, Percentage change; ILD&PS, Iinterstitial lung disease and pulmonary sarcoidosis; DALYs, Disability-adjusted life years; ASPR, Age-standardized prevalence rate; ASIR, Age-standardized incidence rate; ASDR, Age-standardized DALYs rate; ASMR, Age-standardized mortality rate; UI, Uncertainty interval.

**Table S6. Number and age-standardized prevalence of ILD&PS, 1990 vs. 2021 (Nations).**

| **Location** | **Number in 1990  (95% UI)** | **ASPR in 1990  (per 100 000, 95% UI)** | **Number in 2021  (95% UI)** | **ASPR in 2021  (per 100 000, 95% UI)** | **EAPC of ASPR (95% CI)** |
| --- | --- | --- | --- | --- | --- |
| Afghanistan | 1361.29(1111.11-1646.35) | 19.21(15.72-22.94) | 3057.86(2502.28-3689.99) | 22.43(19.22-26.37) | 0.76(-13.71-17.65) |
| Albania | 839.16(706.81-1000.54) | 34.90(29.83-41.05) | 1382.47(1206.92-1588.52) | 35.29(30.45-41.21) | 0.24(-14.13-17) |
| Algeria | 3523.58(2875.00-4223.71) | 24.96(20.68-29.66) | 12854.05(11082.96-15092.80) | 32.27(28.09-37.52) | 1.02(-13.45-17.91) |
| American Samoa | 10.41(8.86-12.24) | 32.72(28.47-37.98) | 14.08(12.21-16.05) | 27.89(24.38-31.83) | -0.48(-14.74-16.16) |
| Andorra | 31.97(28.02-36.66) | 53.91(47.40-61.82) | 91.38(81.20-103.03) | 61.12(54.46-68.94) | 0.42(-13.92-17.15) |
| Angola | 707.15(571.05-856.43) | 14.55(12.19-17.15) | 2706.40(2251.73-3204.56) | 18.21(15.79-21.15) | 0.88(-13.62-17.8) |
| Antigua and Barbuda | 7.05(5.82-8.41) | 13.54(11.22-16.33) | 21.26(18.51-24.69) | 19.48(17.00-22.47) | 1.36(-13.17-18.34) |
| Argentina | 17957.69(16008.83-20074.26) | 55.24(49.31-61.68) | 43609.09(40018.77-47261.18) | 78.80(72.20-85.46) | 1.13(-13.35-18.04) |
| Armenia | 1124.67(988.43-1288.71) | 39.33(34.92-44.63) | 1718.45(1540.29-1926.66) | 41.24(36.96-46.66) | 0.63(-13.78-17.45) |
| Australia | 6685.05(5879.27-7563.44) | 34.01(29.87-38.61) | 28564.21(25715.61-31635.27) | 63.59(57.21-70.80) | 2.11(-12.46-19.12) |
| Austria | 4585.80(4012.40-5231.46) | 44.20(38.16-50.73) | 6545.16(5837.19-7295.58) | 42.81(37.81-48.17) | -0.19(-14.44-16.44) |
| Azerbaijan | 1652.18(1442.30-1937.25) | 30.59(26.73-35.39) | 3551.78(3083.46-4116.45) | 32.50(28.66-37.24) | 0.46(-13.94-17.26) |
| Bahamas | 42.99(37.24-49.49) | 25.01(21.96-28.42) | 142.46(127.71-158.22) | 33.32(29.94-36.75) | 1.2(-13.31-18.13) |
| Bahrain | 86.49(71.64-103.79) | 34.88(30.28-40.13) | 642.39(560.25-741.16) | 57.71(51.77-64.39) | 1.98(-12.64-19.05) |
| Bangladesh | 19796.53(17274.25-22701.89) | 38.72(33.99-43.89) | 75226.84(66949.88-84357.12) | 52.03(46.30-58.07) | 0.88(-13.6-17.79) |
| Barbados | 50.92(44.43-58.28) | 18.67(15.95-21.65) | 125.64(112.52-138.59) | 26.14(23.15-29.10) | 1.29(-13.23-18.24) |
| Belarus | 6897.27(6065.30-7839.68) | 55.08(48.29-63.14) | 3548.56(3036.29-4091.63) | 26.34(22.20-30.78) | -2.59(-16.52-13.67) |
| Belgium | 4119.05(3650.40-4675.53) | 27.92(24.49-31.98) | 8666.07(7825.24-9593.33) | 40.21(36.12-44.81) | 1.55(-12.95-18.45) |
| Belize | 34.65(30.58-38.71) | 34.77(30.94-39.03) | 244.46(224.39-264.17) | 72.20(66.59-77.73) | 2.43(-12.26-19.58) |
| Benin | 301.58(244.63-362.17) | 13.07(10.87-15.49) | 788.41(638.63-957.06) | 11.65(9.75-13.81) | -0.32(-14.65-16.4) |
| Bermuda | 28.43(25.62-31.49) | 44.42(40.08-49.05) | 84.01(76.96-91.14) | 67.11(61.48-73.22) | 1.5(-13.04-18.46) |
| Bhutan | 98.36(84.95-114.17) | 36.20(31.71-41.25) | 405.83(363.70-448.14) | 64.85(58.20-71.56) | 2.03(-12.61-19.11) |
| Bolivia (Plurinational State of) | 2332.05(2097.07-2576.87) | 71.89(64.69-79.29) | 10337.29(9421.75-11296.05) | 115.37(105.44-125.77) | 1.76(-12.84-18.8) |
| Bosnia and Herzegovina | 1302.22(1074.59-1570.22) | 28.67(24.10-34.33) | 1480.41(1276.46-1722.33) | 28.33(24.09-33.36) | 0.15(-14.2-16.9) |
| Botswana | 213.63(181.56-249.29) | 33.37(28.78-38.44) | 677.89(590.37-773.64) | 39.36(34.77-44.10) | 0.61(-13.82-17.46) |
| Brazil | 28492.33(23571.02-34167.88) | 27.80(23.25-32.96) | 51549.94(44623.64-59313.07) | 20.50(17.78-23.52) | -1.14(-15.32-15.42) |
| Brunei Darussalam | 177.50(157.85-200.49) | 156.46(139.70-175.22) | 471.24(426.64-526.44) | 127.25(116.01-139.79) | -0.79(-14.97-15.74) |
| Bulgaria | 2910.85(2470.37-3431.82) | 25.32(21.25-29.92) | 2230.62(1898.41-2615.47) | 20.74(17.28-24.64) | -0.66(-14.89-15.94) |
| Burkina Faso | 543.63(430.66-667.33) | 10.77(8.67-13.07) | 1167.92(921.54-1441.11) | 9.70(7.88-11.73) | -0.32(-14.64-16.41) |
| Burundi | 399.44(322.32-479.55) | 14.72(12.24-17.41) | 929.92(759.20-1116.54) | 14.90(12.61-17.37) | 0.08(-14.3-16.87) |
| Cabo Verde | 19.94(15.76-24.51) | 9.19(7.25-11.27) | 58.11(46.92-71.29) | 11.37(9.33-13.75) | 0.88(-13.59-17.76) |
| Cambodia | 480.21(370.39-597.03) | 9.35(7.48-11.37) | 1720.46(1429.92-2064.30) | 12.76(10.76-15.09) | 1.16(-13.37-18.12) |
| Cameroon | 753.40(620.88-902.44) | 14.19(11.98-16.62) | 2027.81(1641.02-2466.13) | 12.00(10.04-14.24) | -0.47(-14.77-16.22) |
| Canada | 26236.85(23708.46-29195.28) | 81.80(73.68-91.33) | 79596.10(72771.39-87024.74) | 117.21(106.28-128.81) | 1.31(-13.14-18.16) |
| Central African Republic | 209.24(170.49-253.33) | 15.27(12.68-17.99) | 412.98(336.19-497.10) | 14.47(12.17-16.83) | -0.15(-14.5-16.61) |
| Chad | 379.26(305.80-453.65) | 11.95(9.84-14.24) | 856.31(690.51-1038.18) | 11.21(9.34-13.26) | -0.09(-14.45-16.68) |
| Chile | 8334.98(7619.19-9097.01) | 80.93(74.16-88.00) | 38771.21(35556.26-42092.35) | 150.81(138.33-163.68) | 2.19(-12.43-19.25) |
| China | 254708.08(208621.75-309660.69) | 27.47(22.74-33.24) | 628382.72(534993.12-737822.25) | 29.42(25.28-34.39) | 0.61(-13.79-17.42) |
| Colombia | 3919.57(3217.69-4691.12) | 19.67(16.51-23.32) | 17061.79(15150.68-19064.27) | 30.97(27.52-34.68) | 1.66(-12.91-18.66) |
| Comoros | 35.28(28.81-42.04) | 15.36(12.87-18.09) | 85.04(71.08-101.04) | 15.28(13.09-17.83) | 0(-14.35-16.76) |
| Congo | 200.81(165.95-238.44) | 16.35(13.68-19.17) | 649.98(548.11-771.83) | 19.44(16.95-22.53) | 0.65(-13.81-17.54) |
| Cook Islands | 6.18(5.40-7.05) | 42.16(37.11-47.97) | 10.20(9.02-11.45) | 44.59(39.21-50.14) | 0.11(-14.24-16.85) |
| Costa Rica | 944.43(845.63-1051.04) | 52.39(47.00-58.22) | 4021.98(3698.89-4353.82) | 73.25(67.37-79.23) | 1.09(-13.39-17.99) |
| Croatia | 1530.69(1256.59-1855.22) | 25.43(20.90-30.83) | 1889.09(1648.60-2160.08) | 29.53(25.34-33.98) | 0.51(-13.89-17.31) |
| Cuba | 1244.29(984.95-1523.97) | 11.86(9.44-14.48) | 2239.49(1887.17-2654.24) | 13.01(10.82-15.52) | 0.41(-13.99-17.23) |
| Cyprus | 566.40(495.14-641.50) | 65.99(58.11-74.80) | 1450.46(1298.51-1610.81) | 68.81(61.71-76.60) | 0.03(-14.26-16.72) |
| Czechia | 2997.08(2519.06-3527.81) | 23.40(19.55-27.70) | 7455.57(6717.28-8374.55) | 41.00(36.43-46.44) | 2.37(-12.28-19.46) |
| Côte d'Ivoire | 740.08(595.57-898.06) | 13.80(11.55-16.29) | 1881.72(1521.46-2263.25) | 12.22(10.33-14.41) | -0.37(-14.69-16.34) |
| Democratic People's Republic of Korea | 3075.63(2573.83-3670.71) | 18.05(15.28-21.22) | 6048.68(5158.13-7031.47) | 18.01(15.45-20.86) | 0.13(-14.21-16.87) |
| Democratic Republic of the Congo | 3107.79(2530.21-3724.67) | 17.02(14.19-20.16) | 8300.82(6945.06-9853.29) | 18.31(15.67-21.19) | 0.31(-14.11-17.14) |
| Denmark | 3385.93(3029.23-3785.89) | 45.66(40.44-51.82) | 5864.99(5320.92-6474.77) | 55.43(49.68-61.61) | 0.61(-13.75-17.36) |
| Djibouti | 28.42(22.70-34.63) | 15.48(12.89-18.22) | 137.00(114.38-163.96) | 16.68(14.38-19.36) | 0.24(-14.15-17.04) |
| Dominica | 9.40(7.96-11.02) | 16.04(13.47-18.84) | 18.21(16.03-20.45) | 22.13(19.45-24.95) | 1.16(-13.34-18.09) |
| Dominican Republic | 497.89(395.21-608.10) | 11.08(8.96-13.38) | 1702.72(1456.09-1992.07) | 16.19(13.91-18.87) | 1.44(-13.11-18.43) |
| Ecuador | 2312.27(2053.05-2609.11) | 42.27(37.88-47.13) | 13354.98(12228.44-14454.96) | 81.93(74.99-88.64) | 2.98(-11.79-20.22) |
| Egypt | 7258.61(5802.99-8913.44) | 21.88(17.98-26.23) | 24036.49(20382.08-28451.78) | 31.91(27.55-37.03) | 1.31(-13.21-18.27) |
| El Salvador | 843.38(733.17-965.78) | 27.27(23.88-30.97) | 2915.61(2641.08-3212.33) | 47.07(42.54-51.90) | 2.22(-12.43-19.33) |
| Equatorial Guinea | 27.83(22.57-33.66) | 12.32(10.16-14.59) | 150.94(126.05-178.68) | 22.97(19.87-26.58) | 2.63(-12.1-19.85) |
| Eritrea | 194.96(154.92-240.55) | 12.69(10.49-15.06) | 520.42(429.38-627.13) | 14.20(12.20-16.47) | 0.22(-14.17-17.03) |
| Estonia | 542.00(462.27-634.13) | 28.74(24.30-33.44) | 678.61(599.65-767.49) | 34.72(30.04-39.61) | 0.85(-13.57-17.68) |
| Eswatini | 109.61(91.76-129.44) | 32.04(27.48-37.12) | 209.84(180.99-243.90) | 30.90(27.33-34.97) | -0.21(-14.52-16.51) |
| Ethiopia | 2674.70(2122.86-3258.37) | 10.87(8.83-13.03) | 6548.19(5325.02-7875.28) | 12.04(10.08-14.25) | 0.48(-13.96-17.34) |
| Fiji | 123.60(102.30-148.30) | 22.99(19.39-27.21) | 212.55(180.96-246.26) | 23.77(20.33-27.33) | 0.11(-14.26-16.87) |
| Finland | 3027.65(2665.38-3447.75) | 44.09(38.63-50.57) | 6884.05(6207.14-7643.03) | 59.37(53.44-66.30) | 0.99(-13.43-17.81) |
| France | 27440.07(24246.17-31249.51) | 35.36(30.97-40.64) | 56617.44(51405.54-62350.41) | 45.28(40.41-50.68) | 0.92(-13.49-17.72) |
| Gabon | 108.95(91.49-129.22) | 17.87(15.17-21.03) | 256.38(220.37-298.02) | 21.30(18.56-24.30) | 0.57(-13.87-17.44) |
| Gambia | 58.31(46.66-71.50) | 13.14(10.84-15.45) | 148.61(120.49-178.98) | 11.79(9.90-14.01) | -0.36(-14.67-16.35) |
| Georgia | 1677.62(1447.14-1943.53) | 27.06(23.54-31.41) | 1428.45(1291.12-1587.58) | 26.41(23.71-29.49) | 0.26(-14.1-17.02) |
| Germany | 45006.77(39725.60-50985.51) | 37.80(33.24-42.88) | 78725.34(71096.12-86635.16) | 45.58(40.54-50.95) | 1.03(-13.4-17.87) |
| Ghana | 1237.21(1032.30-1459.21) | 16.59(14.11-19.39) | 3550.10(2996.05-4139.32) | 17.51(15.08-19.95) | 0.02(-14.35-16.8) |
| Greece | 2870.72(2460.61-3385.74) | 19.62(16.77-23.24) | 6983.18(6233.84-7812.96) | 32.66(28.89-37.03) | 2.19(-12.41-19.23) |
| Greenland | 38.67(34.12-43.65) | 105.95(94.68-117.97) | 78.83(71.30-87.02) | 116.22(105.38-127.82) | 0.43(-13.92-17.16) |
| Grenada | 10.75(9.26-12.45) | 15.92(13.60-18.54) | 28.34(24.99-32.33) | 24.34(21.58-27.62) | 1.47(-13.08-18.46) |
| Guam | 115.51(105.24-127.01) | 108.71(99.60-118.05) | 177.69(163.49-192.49) | 93.53(85.94-101.75) | -0.47(-14.73-16.17) |
| Guatemala | 1264.10(1121.18-1425.97) | 33.27(29.79-36.96) | 4808.20(4384.86-5233.19) | 42.28(38.73-45.86) | 0.99(-13.5-17.9) |
| Guinea | 479.88(395.41-574.79) | 13.14(10.94-15.53) | 811.67(664.91-975.49) | 11.58(9.75-13.67) | -0.39(-14.71-16.32) |
| Guinea-Bissau | 61.92(50.42-74.48) | 12.79(10.73-15.05) | 114.24(91.42-140.06) | 10.97(9.20-13.02) | -0.48(-14.78-16.22) |
| Guyana | 72.24(60.49-85.55) | 16.18(13.85-18.86) | 166.71(147.47-188.50) | 24.07(21.49-27.03) | 1.38(-13.17-18.37) |
| Haiti | 582.71(486.35-686.61) | 15.57(13.26-18.07) | 1682.07(1448.56-1936.42) | 19.31(17.00-21.93) | 0.84(-13.64-17.75) |
| Honduras | 740.81(645.30-851.98) | 33.33(29.25-38.05) | 3542.63(3168.55-3951.45) | 52.63(47.15-58.61) | 1.62(-12.96-18.64) |
| Hungary | 4560.84(3977.62-5203.16) | 33.47(28.98-38.50) | 6184.73(5521.33-6909.37) | 38.74(33.99-44.02) | 0.73(-13.69-17.56) |
| Iceland | 85.85(75.41-97.82) | 31.07(27.00-35.67) | 231.14(208.18-255.42) | 42.32(38.01-47.21) | 1.33(-13.15-18.22) |
| India | 244066.53(203845.90-288777.71) | 48.90(41.02-57.96) | 654924.60(565718.36-757500.39) | 52.55(45.53-60.83) | 0.37(-14.04-17.2) |
| Indonesia | 15485.11(12426.06-19024.07) | 14.06(11.51-16.96) | 45334.70(38244.65-53946.45) | 17.09(14.56-20.02) | 0.7(-13.75-17.56) |
| Iran (Islamic Republic of) | 5515.03(4344.76-6788.00) | 17.62(14.24-21.48) | 17455.28(14267.33-20992.91) | 19.77(16.39-23.50) | 0.47(-13.93-17.28) |
| Iraq | 1953.45(1580.73-2358.87) | 20.81(17.10-24.92) | 8451.02(7129.40-9861.12) | 28.98(25.03-33.38) | 1.38(-13.15-18.35) |
| Ireland | 1974.31(1774.29-2204.78) | 48.65(43.66-54.41) | 7355.59(6625.63-8117.09) | 94.14(84.70-103.65) | 2.25(-12.36-19.29) |
| Israel | 1433.96(1264.49-1627.51) | 29.83(26.21-34.08) | 4148.09(3741.59-4636.55) | 35.50(31.62-39.86) | 0.74(-13.66-17.55) |
| Italy | 36609.35(30287.96-43955.99) | 45.82(37.58-55.50) | 72287.63(63413.43-83209.24) | 59.00(51.46-68.72) | 1.09(-13.36-17.95) |
| Jamaica | 232.48(194.26-275.20) | 13.19(10.99-15.63) | 550.30(477.52-626.56) | 17.83(15.45-20.29) | 1.05(-13.44-17.97) |
| Japan | 247461.38(210489.58-291323.90) | 145.90(123.98-170.58) | 520917.69(451216.29-600474.46) | 160.24(140.33-183.48) | 0.36(-13.96-17.06) |
| Jordan | 866.75(764.92-982.38) | 56.45(50.59-63.15) | 5962.76(5322.04-6607.90) | 70.50(63.77-77.02) | 0.87(-13.58-17.73) |
| Kazakhstan | 3098.68(2597.26-3639.66) | 22.46(19.22-26.22) | 6181.10(5432.30-6995.37) | 32.29(28.60-36.44) | 1.59(-12.97-18.59) |
| Kenya | 1625.04(1305.65-1966.87) | 16.63(13.69-19.95) | 4619.57(3841.53-5566.44) | 16.37(13.85-19.30) | -0.3(-14.62-16.42) |
| Kiribati | 15.46(13.52-17.85) | 29.97(26.29-34.07) | 30.71(27.19-34.70) | 31.10(27.83-34.89) | 0.14(-14.24-16.93) |
| Kuwait | 337.12(282.30-403.31) | 42.00(37.21-47.61) | 1933.48(1652.08-2248.60) | 53.86(48.44-59.79) | 1.04(-13.4-17.9) |
| Kyrgyzstan | 603.36(494.61-721.65) | 18.81(15.66-22.42) | 886.13(710.23-1081.85) | 15.29(12.48-18.56) | -0.77(-14.99-15.82) |
| Lao People's Democratic Republic | 256.93(211.40-310.79) | 11.28(9.42-13.40) | 799.33(669.93-940.82) | 15.68(13.38-18.19) | 1.15(-13.37-18.1) |
| Latvia | 1384.97(1197.41-1594.33) | 42.00(36.23-48.61) | 817.45(702.95-944.15) | 29.23(24.62-34.21) | -0.61(-14.82-15.97) |
| Lebanon | 613.93(507.04-739.47) | 26.24(21.94-31.60) | 2054.17(1768.64-2387.37) | 34.53(29.84-40.08) | 1.07(-13.4-17.96) |
| Lesotho | 269.42(226.01-317.21) | 29.38(24.87-34.33) | 370.74(322.08-427.08) | 30.04(26.51-34.13) | 0.06(-14.3-16.83) |
| Liberia | 162.96(130.25-198.26) | 12.36(10.04-14.95) | 343.57(267.25-421.88) | 11.64(9.56-13.88) | 0.04(-14.34-16.83) |
| Libya | 585.45(481.15-700.68) | 26.97(22.29-32.03) | 1844.23(1560.39-2191.73) | 29.02(25.18-33.91) | 0.43(-13.96-17.23) |
| Lithuania | 951.92(789.93-1125.70) | 22.41(18.49-26.62) | 883.88(759.04-1028.22) | 21.54(18.19-25.34) | -0.22(-14.51-16.45) |
| Luxembourg | 160.67(140.49-184.33) | 30.48(26.47-35.35) | 416.49(373.23-466.45) | 41.45(37.00-46.72) | 1.03(-13.4-17.86) |
| Madagascar | 950.24(787.83-1133.10) | 16.11(13.56-19.06) | 2603.17(2191.64-3089.43) | 18.21(15.73-21.02) | 0.35(-14.07-17.18) |
| Malawi | 591.72(466.46-726.28) | 12.60(10.27-15.07) | 1312.98(1074.02-1565.02) | 13.91(11.77-16.34) | 0.37(-14.05-17.21) |
| Malaysia | 1754.19(1480.60-2050.99) | 17.61(15.22-20.35) | 7924.16(7007.84-9004.38) | 26.62(23.71-30.13) | 1.49(-13.06-18.47) |
| Maldives | 82.80(73.64-93.74) | 81.57(73.18-91.64) | 456.22(415.06-504.45) | 124.13(112.34-136.75) | 1.38(-13.16-18.35) |
| Mali | 648.97(536.00-778.30) | 13.68(11.45-16.19) | 1601.00(1340.99-1896.62) | 14.35(12.33-16.65) | 0.17(-14.22-16.99) |
| Malta | 219.11(195.74-246.14) | 50.80(45.23-56.99) | 744.53(678.25-811.03) | 80.73(73.57-88.26) | 1.56(-12.94-18.49) |
| Marshall Islands | 10.16(8.92-11.58) | 42.68(37.88-47.87) | 21.89(19.68-24.41) | 46.14(41.52-50.96) | 0.2(-14.18-16.99) |
| Mauritania | 151.38(124.69-182.62) | 13.27(11.09-15.66) | 327.61(270.52-394.64) | 12.80(10.81-15.11) | -0.09(-14.43-16.66) |
| Mauritius | 376.86(335.12-420.22) | 50.48(45.08-56.05) | 2038.15(1845.50-2226.99) | 107.87(98.29-117.39) | 2.52(-12.18-19.69) |
| Mexico | 23858.40(20056.43-28136.20) | 52.00(43.92-61.43) | 70394.01(61530.92-80826.22) | 55.05(48.13-63.02) | 0.02(-14.31-16.75) |
| Micronesia (Federated States of) | 25.95(22.77-29.32) | 40.93(36.40-46.13) | 41.21(36.95-45.87) | 45.76(41.16-50.51) | 0.39(-14.02-17.22) |
| Monaco | 28.38(25.05-32.47) | 45.47(39.74-52.31) | 43.12(38.74-48.06) | 50.12(44.58-56.64) | 0.38(-13.95-17.1) |
| Mongolia | 336.60(291.19-389.48) | 28.25(24.74-32.11) | 911.14(795.38-1047.16) | 33.56(29.81-37.86) | 0.62(-13.8-17.46) |
| Montenegro | 142.24(115.24-172.31) | 21.91(17.84-26.50) | 172.65(143.87-206.46) | 20.26(16.77-24.42) | -0.06(-14.36-16.64) |
| Morocco | 3603.22(2912.89-4304.19) | 22.43(18.54-26.63) | 11544.69(9993.70-13497.94) | 31.33(27.23-36.39) | 1.19(-13.32-18.12) |
| Mozambique | 805.10(638.55-982.74) | 11.09(8.99-13.30) | 1798.66(1430.69-2194.35) | 12.45(10.30-14.84) | 0.37(-14.05-17.22) |
| Myanmar | 3713.79(3113.00-4400.81) | 14.94(12.64-17.45) | 13316.83(11490.71-15226.18) | 26.32(22.87-29.91) | 2.06(-12.59-19.17) |
| Namibia | 238.64(202.03-277.98) | 32.54(28.04-37.51) | 574.73(502.88-653.30) | 36.24(32.03-40.49) | 0.37(-14.03-17.19) |
| Nauru | 3.59(3.16-4.05) | 54.37(48.36-60.77) | 4.30(3.85-4.80) | 53.81(48.39-59.27) | -0.05(-14.39-16.7) |
| Nepal | 4758.40(4147.17-5451.75) | 47.39(41.33-54.05) | 18223.18(16388.60-20247.34) | 75.39(68.01-83.44) | 1.28(-13.26-18.26) |
| Netherlands | 5499.49(4730.10-6425.02) | 28.90(24.78-33.92) | 16419.82(14723.44-18292.74) | 49.99(44.44-56.42) | 1.91(-12.63-18.89) |
| New Zealand | 1936.61(1622.56-2309.42) | 49.45(41.36-59.08) | 4391.07(3830.87-4976.63) | 52.30(45.95-59.41) | 0.17(-14.13-16.86) |
| Nicaragua | 386.53(323.93-458.78) | 22.49(19.19-26.36) | 1777.56(1578.76-1997.62) | 35.18(31.38-39.14) | 1.89(-12.73-18.96) |
| Niger | 457.59(367.29-557.12) | 12.86(10.68-15.16) | 1156.61(934.12-1416.09) | 10.76(8.94-12.79) | -0.5(-14.8-16.2) |
| Nigeria | 7394.68(5884.84-8975.07) | 14.63(11.80-17.66) | 14938.48(11950.19-18256.15) | 12.88(10.60-15.39) | -0.33(-14.65-16.39) |
| Niue | 1.07(0.95-1.19) | 51.46(45.78-57.76) | 1.10(0.99-1.23) | 55.37(49.58-61.64) | 0.26(-14.11-17.04) |
| North Macedonia | 424.31(341.39-512.38) | 20.84(16.87-25.11) | 624.04(521.08-740.95) | 20.13(16.76-24.02) | 0.01(-14.32-16.73) |
| Northern Mariana Islands | 19.88(17.01-23.26) | 64.52(55.90-74.03) | 35.03(30.91-39.13) | 62.50(55.44-69.69) | -0.34(-14.62-16.32) |
| Norway | 3619.89(3030.93-4311.32) | 59.00(49.53-70.25) | 5748.35(4947.92-6657.85) | 62.93(54.24-73.18) | 0.66(-13.72-17.42) |
| Oman | 232.03(184.97-285.09) | 23.62(19.80-27.87) | 1131.48(934.98-1363.26) | 37.93(33.24-43.31) | 1.83(-12.77-18.87) |
| Pakistan | 24918.09(20836.54-29434.38) | 41.83(35.21-49.43) | 42627.59(36267.34-49584.38) | 31.24(27.07-36.04) | -0.96(-15.17-15.64) |
| Palau | 7.13(6.30-8.00) | 57.05(50.69-63.96) | 13.58(12.17-15.12) | 58.13(52.29-64.36) | 0.01(-14.33-16.74) |
| Palestine | 703.74(624.89-799.37) | 75.37(67.22-85.40) | 3007.40(2731.90-3278.15) | 104.41(95.17-113.28) | 1.16(-13.33-18.08) |
| Panama | 446.25(392.10-508.50) | 28.20(24.88-31.97) | 2347.76(2134.01-2570.98) | 53.18(48.36-58.22) | 2.4(-12.27-19.53) |
| Papua New Guinea | 1106.69(966.12-1258.13) | 45.09(39.92-50.90) | 3730.14(3346.39-4137.20) | 53.55(48.87-59.11) | 0.5(-13.93-17.36) |
| Paraguay | 320.77(266.64-381.00) | 13.28(11.30-15.52) | 1125.23(988.90-1283.79) | 18.62(16.53-21.10) | 1.31(-13.22-18.28) |
| Peru | 11290.23(10337.35-12376.48) | 93.07(85.14-102.06) | 55994.37(52200.25-59884.41) | 167.38(155.61-179.28) | 2.41(-12.28-19.57) |
| Philippines | 3624.69(2769.58-4534.56) | 10.69(8.52-13.22) | 7770.16(6089.10-9668.10) | 8.34(6.71-10.28) | -0.88(-15.1-15.72) |
| Poland | 18451.17(15226.95-22036.19) | 42.81(35.21-51.26) | 24978.22(21722.71-28714.83) | 42.62(36.66-49.33) | 0.21(-14.14-16.95) |
| Portugal | 3968.33(3421.01-4584.54) | 29.15(25.10-33.75) | 11894.88(10833.06-12987.77) | 53.49(48.34-59.04) | 2.06(-12.52-19.06) |
| Puerto Rico | 951.37(841.91-1081.63) | 26.15(23.03-29.70) | 2816.84(2555.32-3104.72) | 45.10(40.75-49.78) | 1.88(-12.71-18.92) |
| Qatar | 71.01(56.25-88.36) | 30.64(26.47-35.59) | 688.34(545.72-845.74) | 34.32(30.02-39.42) | 0.39(-13.98-17.16) |
| Republic of Korea | 25691.96(22053.98-29679.46) | 73.22(63.74-83.72) | 115293.26(103559.41-128325.24) | 127.46(114.18-141.86) | 2.24(-12.37-19.29) |
| Republic of Moldova | 982.11(794.35-1191.17) | 21.22(17.22-25.73) | 1010.89(840.44-1203.47) | 19.71(16.24-23.57) | -0.13(-14.42-16.55) |
| Romania | 17323.04(15543.34-19514.69) | 62.42(55.81-70.36) | 15301.68(13730.37-17166.95) | 48.94(43.49-55.29) | -0.63(-14.87-15.99) |
| Russian Federation | 43245.58(34847.50-52665.33) | 24.39(19.70-29.80) | 34023.77(27774.63-41038.87) | 16.69(13.49-20.22) | -1.36(-15.47-15.11) |
| Rwanda | 484.86(394.16-583.29) | 14.27(11.90-16.95) | 1322.82(1104.61-1558.36) | 17.11(14.64-19.82) | 0.72(-13.75-17.61) |
| Saint Kitts and Nevis | 7.59(6.66-8.68) | 21.04(18.40-24.15) | 25.46(22.66-28.55) | 35.33(31.64-39.36) | 1.84(-12.76-18.87) |
| Saint Lucia | 18.84(16.26-21.55) | 20.63(17.93-23.52) | 82.04(74.16-90.39) | 34.60(31.37-38.19) | 1.8(-12.79-18.84) |
| Saint Vincent and the Grenadines | 9.26(7.60-11.10) | 12.48(10.24-15.02) | 26.25(22.84-29.93) | 18.70(16.21-21.25) | 1.51(-13.05-18.51) |
| Samoa | 46.87(41.54-53.22) | 44.40(39.72-50.18) | 83.01(74.43-91.83) | 49.98(44.99-55.20) | 0.42(-13.99-17.23) |
| San Marino | 7.66(6.46-9.07) | 23.88(19.90-28.55) | 13.11(11.30-15.24) | 21.60(18.24-25.59) | -0.23(-14.48-16.38) |
| Sao Tome and Principe | 15.10(13.00-17.42) | 22.18(19.22-25.53) | 37.13(32.33-42.51) | 28.07(24.93-31.84) | 0.77(-13.7-17.67) |
| Saudi Arabia | 3822.25(3354.56-4335.60) | 57.39(50.69-64.33) | 25346.22(22608.14-28192.12) | 105.48(96.07-115.58) | 2.3(-12.37-19.42) |
| Senegal | 506.85(413.57-606.07) | 13.48(11.25-15.86) | 1183.70(971.31-1415.73) | 12.62(10.61-14.80) | -0.17(-14.51-16.57) |
| Serbia | 2464.50(2048.57-2923.26) | 21.40(17.74-25.39) | 3219.63(2791.23-3709.09) | 23.60(20.29-27.58) | 0.88(-13.58-17.75) |
| Seychelles | 7.76(6.37-9.31) | 13.60(11.24-16.34) | 20.91(17.78-24.54) | 17.25(14.76-20.11) | 0.69(-13.74-17.53) |
| Sierra Leone | 295.30(237.93-354.34) | 12.58(10.35-14.98) | 541.55(438.70-658.76) | 10.98(9.14-13.14) | -0.39(-14.71-16.32) |
| Singapore | 1241.38(1060.66-1469.70) | 46.78(40.30-54.83) | 5436.22(4796.52-6136.60) | 63.53(56.09-71.65) | 1.26(-13.18-18.11) |
| Slovakia | 1640.73(1369.07-1945.77) | 28.16(23.45-33.52) | 2886.44(2539.12-3281.62) | 34.65(30.33-40.01) | 0.96(-13.49-17.82) |
| Slovenia | 649.03(541.76-768.33) | 27.37(22.75-32.51) | 1434.23(1260.25-1642.45) | 39.86(34.33-46.25) | 1.81(-12.76-18.81) |
| Solomon Islands | 56.29(47.75-66.00) | 30.39(26.06-35.26) | 152.96(132.41-175.43) | 32.40(28.66-36.80) | 0.12(-14.26-16.91) |
| Somalia | 464.74(367.91-569.27) | 14.35(11.86-17.08) | 1105.94(885.24-1345.27) | 12.76(10.77-15.01) | -0.37(-14.69-16.36) |
| South Africa | 11429.13(9571.26-13501.17) | 51.21(43.35-60.37) | 22248.89(19140.57-25910.61) | 44.80(38.67-51.87) | -0.53(-14.81-16.14) |
| South Sudan | 436.58(360.91-516.72) | 14.86(12.45-17.53) | 618.86(506.15-745.89) | 12.66(10.71-14.81) | -0.43(-14.74-16.28) |
| Spain | 27999.18(25256.69-30834.46) | 52.08(47.00-57.68) | 69347.86(63065.02-76019.61) | 74.46(67.47-81.61) | 1.26(-13.19-18.11) |
| Sri Lanka | 1697.04(1408.04-2040.58) | 14.33(12.13-16.84) | 6459.03(5649.69-7381.85) | 23.40(20.51-26.64) | 1.73(-12.86-18.76) |
| Sudan | 2262.24(1801.10-2738.42) | 20.80(16.91-24.87) | 6724.60(5631.78-7926.87) | 27.41(23.63-31.75) | 1.05(-13.45-17.98) |
| Suriname | 49.19(41.78-57.82) | 17.74(15.24-20.75) | 159.31(141.84-178.54) | 24.90(22.15-27.78) | 1.39(-13.16-18.37) |
| Sweden | 7615.51(6405.42-9056.74) | 55.59(46.89-65.91) | 12046.22(10410.40-13898.05) | 60.40(52.06-69.76) | 0.19(-14.12-16.88) |
| Switzerland | 3918.02(3476.81-4413.56) | 40.16(35.04-45.81) | 7361.43(6627.12-8135.51) | 44.61(39.77-50.11) | 0.39(-13.95-17.11) |
| Syrian Arab Republic | 1622.95(1341.12-1941.51) | 26.88(22.67-31.61) | 5292.05(4615.19-6095.78) | 37.19(32.66-42.52) | 1.14(-13.35-18.06) |
| Taiwan (Province of China) | 2484.74(2068.98-2968.10) | 14.83(12.55-17.44) | 13523.83(11988.34-15264.86) | 32.44(28.75-36.57) | 2.91(-11.81-20.08) |
| Tajikistan | 1824.61(1624.55-2041.86) | 64.03(56.84-71.85) | 4375.12(3958.42-4840.80) | 70.37(64.23-77.31) | 0.59(-13.84-17.43) |
| Thailand | 4459.39(3542.35-5470.30) | 11.11(9.10-13.36) | 13210.26(11147.49-15755.77) | 12.40(10.39-14.93) | 0.22(-14.14-16.99) |
| Timor-Leste | 40.67(32.39-49.92) | 12.01(10.01-14.19) | 138.84(119.14-161.52) | 15.33(13.23-17.84) | 1.03(-13.48-17.97) |
| Togo | 210.15(167.99-255.84) | 13.26(10.98-15.70) | 587.04(474.79-716.31) | 11.40(9.47-13.39) | -0.51(-14.8-16.19) |
| Tokelau | 0.56(0.50-0.63) | 42.31(37.45-47.74) | 0.67(0.61-0.75) | 47.43(42.61-52.84) | 0.41(-13.99-17.23) |
| Tonga | 21.06(18.21-24.25) | 32.61(28.30-37.46) | 31.97(28.45-35.79) | 37.12(33.13-41.51) | 0.39(-14.01-17.19) |
| Trinidad and Tobago | 218.48(192.32-247.34) | 24.37(21.57-27.52) | 628.42(565.30-697.03) | 33.05(29.61-36.80) | 1.58(-12.98-18.57) |
| Tunisia | 1390.12(1135.67-1672.08) | 24.89(20.62-29.56) | 4614.13(3968.27-5363.80) | 33.61(29.03-39.01) | 1.06(-13.41-17.95) |
| Turkey | 10850.13(9210.75-12763.27) | 27.39(23.47-31.74) | 40309.15(35469.60-45852.15) | 41.66(36.71-47.36) | 1.79(-12.79-18.82) |
| Turkmenistan | 538.86(454.66-629.40) | 24.87(21.35-28.65) | 804.36(653.67-969.22) | 17.30(14.33-20.60) | -1.07(-15.24-15.48) |
| Tuvalu | 3.25(2.88-3.68) | 41.62(37.05-46.94) | 5.68(5.10-6.31) | 49.86(44.82-55.09) | 0.51(-13.91-17.35) |
| Uganda | 1119.75(914.14-1358.31) | 14.77(12.32-17.58) | 3157.92(2638.54-3752.55) | 17.08(14.67-19.80) | 0.54(-13.9-17.41) |
| Ukraine | 36875.03(31108.77-43848.57) | 54.13(45.80-64.12) | 11830.92(9828.99-14081.87) | 18.48(15.30-22.13) | -4(-17.73-12.03) |
| United Arab Emirates | 316.70(254.31-386.65) | 34.80(29.67-40.99) | 4119.40(3426.33-4855.87) | 41.46(36.26-47.02) | 0.54(-13.86-17.35) |
| United Kingdom | 65088.03(55385.78-76392.36) | 78.82(67.13-92.24) | 111573.16(97864.82-126602.62) | 91.50(80.64-103.94) | 0.8(-13.59-17.6) |
| United Republic of Tanzania | 1606.86(1292.32-1941.70) | 12.51(10.27-14.96) | 4635.55(3835.04-5518.55) | 14.81(12.56-17.30) | 0.64(-13.82-17.52) |
| United States of America | 367106.25(314031.67-430934.92) | 119.75(102.77-140.78) | 708091.63(621857.11-808616.38) | 128.72(113.85-146.06) | 0.1(-14.19-16.76) |
| United States Virgin Islands | 26.09(22.82-29.80) | 28.60(25.30-32.56) | 74.86(67.99-82.38) | 44.46(40.21-49.17) | 1.62(-12.94-18.61) |
| Uruguay | 1548.25(1347.15-1778.57) | 41.15(35.63-47.19) | 3224.46(2940.28-3516.86) | 62.12(56.30-68.22) | 1.43(-13.08-18.37) |
| Uzbekistan | 6427.43(5740.53-7237.97) | 52.22(46.60-58.71) | 11493.83(10154.98-12963.02) | 39.88(35.95-44.47) | -1.21(-15.37-15.32) |
| Vanuatu | 34.51(29.87-39.86) | 38.41(33.81-44.05) | 93.89(83.36-104.56) | 39.95(35.67-44.21) | 0.06(-14.31-16.85) |
| Venezuela (Bolivarian Republic of) | 2742.37(2340.78-3190.33) | 25.73(22.40-29.42) | 9231.26(8220.86-10246.70) | 30.52(27.34-33.87) | 0.78(-13.67-17.66) |
| Viet Nam | 5132.44(4158.59-6249.54) | 12.18(9.99-14.73) | 21575.45(18483.83-25210.98) | 20.89(18.00-24.26) | 1.95(-12.67-19.03) |
| Yemen | 1258.01(998.59-1530.97) | 20.68(16.84-24.72) | 4420.85(3670.37-5277.40) | 24.25(20.78-28.25) | 0.79(-13.68-17.68) |
| Zambia | 474.72(382.25-571.85) | 13.71(11.40-16.25) | 1481.10(1224.98-1759.89) | 16.40(14.21-19.07) | 0.69(-13.78-17.58) |
| Zimbabwe | 1131.16(920.23-1370.64) | 22.64(18.83-26.91) | 1780.67(1437.54-2148.07) | 19.73(16.47-23.27) | -0.62(-14.88-16.03) |

ILD&PS, Interstitial lung disease and pulmonary sarcoidosis; ASPR, Age-standardized prevalence rate; EAPC, Estimated annual percentage change; UI, Uncertainty interval; CI, Confidence interval.

**Table S7. Number and age-standardized incidence of ILD&PS, 1990 vs. 2021 (Nations).**

| **Location** | **Number in 1990  (95% UI)** | **ASIR in 1990  (per 100 000, 95% UI)** | **Number in 2021  (95% UI)** | **ASIR in 2021  (per 100 000, 95% UI)** | **EAPC of ASIR (95% CI)** |
| --- | --- | --- | --- | --- | --- |
| Afghanistan | 125.51(106.59-146.97) | 1.89(1.61-2.18) | 293.62(250.23-346.45) | 2.02(1.78-2.26) | 0.46(-15.54-19.5) |
| Albania | 68.15(59.92-77.59) | 2.72(2.41-3.03) | 88.92(79.72-98.14) | 2.39(2.14-2.66) | -0.28(-16.08-18.49) |
| Algeria | 306.43(258.91-359.69) | 2.18(1.87-2.49) | 991.61(877.52-1134.08) | 2.52(2.25-2.83) | 0.66(-15.37-19.72) |
| American Samoa | 0.80(0.70-0.92) | 2.31(2.07-2.60) | 0.96(0.86-1.06) | 1.96(1.77-2.17) | -0.57(-16.64-18.6) |
| Andorra | 2.91(2.58-3.29) | 4.94(4.38-5.52) | 8.54(7.68-9.45) | 5.75(5.17-6.34) | 0.44(-15.69-19.65) |
| Angola | 78.93(66.98-92.19) | 1.73(1.49-1.97) | 267.98(233.69-308.23) | 1.80(1.61-2.00) | 0.24(-16.04-19.67) |
| Antigua and Barbuda | 0.63(0.55-0.73) | 1.17(1.02-1.34) | 1.84(1.67-2.04) | 1.74(1.58-1.92) | 1.5(-14.82-20.94) |
| Argentina | 1723.02(1566.79-1880.81) | 5.42(4.94-5.91) | 4304.85(4025.87-4571.08) | 7.83(7.35-8.32) | 1.24(-15.15-20.81) |
| Armenia | 109.70(98.31-121.14) | 3.84(3.48-4.21) | 170.90(156.82-186.54) | 4.11(3.76-4.48) | 0.72(-15.29-19.74) |
| Australia | 632.64(574.30-697.26) | 3.26(2.95-3.60) | 2925.43(2653.62-3198.62) | 6.61(6.05-7.19) | 2.45(-14.06-22.12) |
| Austria | 287.46(261.71-319.39) | 2.90(2.63-3.25) | 433.77(401.87-467.79) | 3.02(2.78-3.29) | 0.26(-15.9-19.51) |
| Azerbaijan | 156.84(139.40-175.22) | 2.91(2.61-3.24) | 302.48(271.38-334.82) | 2.96(2.68-3.27) | 0.16(-15.58-18.84) |
| Bahamas | 4.14(3.71-4.64) | 2.36(2.13-2.59) | 13.08(12.10-14.07) | 3.12(2.92-3.35) | 1.12(-15.11-20.45) |
| Bahrain | 8.46(7.25-9.85) | 3.56(3.19-3.95) | 60.04(54.21-66.86) | 5.73(5.24-6.26) | 1.86(-14.33-21.12) |
| Bangladesh | 2494.76(2224.29-2775.32) | 4.91(4.37-5.45) | 7446.17(6711.64-8190.08) | 5.17(4.67-5.66) | 0.22(-16.03-19.62) |
| Barbados | 4.84(4.39-5.37) | 1.77(1.59-1.96) | 12.02(11.16-12.88) | 2.54(2.35-2.73) | 1.34(-14.96-20.77) |
| Belarus | 411.13(369.52-457.28) | 3.44(3.10-3.80) | 155.62(138.46-174.01) | 1.37(1.21-1.54) | -3.36(-18.79-15.01) |
| Belgium | 352.94(323.42-385.88) | 2.48(2.26-2.72) | 782.96(718.62-849.84) | 3.76(3.46-4.10) | 1.62(-14.68-21.04) |
| Belize | 4.03(3.63-4.41) | 3.94(3.56-4.32) | 24.68(23.21-26.24) | 7.34(6.92-7.79) | 2.02(-14.39-21.56) |
| Benin | 31.40(26.51-36.46) | 1.32(1.15-1.51) | 73.15(62.08-86.21) | 1.01(0.89-1.14) | -0.74(-16.93-18.61) |
| Bermuda | 2.97(2.73-3.21) | 4.74(4.36-5.10) | 8.21(7.61-8.82) | 6.58(6.17-7.03) | 1.11(-15.15-20.48) |
| Bhutan | 12.76(11.23-14.39) | 4.92(4.36-5.49) | 43.68(39.85-47.60) | 6.94(6.33-7.59) | 1.52(-14.81-20.98) |
| Bolivia (Plurinational State of) | 369.22(331.93-407.43) | 12.58(11.26-13.89) | 1570.78(1442.07-1705.54) | 18.48(16.97-20.02) | 1.56(-14.75-21) |
| Bosnia and Herzegovina | 96.03(82.67-110.66) | 2.13(1.85-2.44) | 84.51(76.17-93.40) | 1.78(1.59-2.00) | -0.5(-16.26-18.22) |
| Botswana | 22.67(19.66-25.76) | 3.67(3.22-4.10) | 62.73(56.49-69.68) | 3.63(3.31-3.96) | 0.13(-16.26-19.71) |
| Brazil | 2544.65(2152.42-2999.03) | 2.46(2.10-2.85) | 6512.98(5704.96-7308.73) | 2.63(2.30-2.96) | 0.2(-16.15-19.73) |
| Brunei Darussalam | 16.08(14.42-17.90) | 12.98(11.70-14.39) | 42.11(38.58-45.85) | 10.42(9.65-11.34) | -0.88(-16.77-18.03) |
| Bulgaria | 165.75(148.52-185.97) | 1.60(1.42-1.82) | 109.18(97.65-122.36) | 1.19(1.04-1.37) | -1.07(-16.72-17.52) |
| Burkina Faso | 52.76(43.69-62.21) | 1.05(0.89-1.21) | 104.03(85.99-123.82) | 0.81(0.70-0.93) | -0.61(-16.89-18.85) |
| Burundi | 44.35(37.34-51.54) | 1.67(1.43-1.90) | 101.76(87.45-117.55) | 1.67(1.48-1.88) | 0.24(-16.19-19.89) |
| Cabo Verde | 1.71(1.39-2.02) | 0.73(0.61-0.87) | 4.35(3.67-5.08) | 0.81(0.70-0.93) | 0.71(-15.58-20.14) |
| Cambodia | 52.35(43.00-62.43) | 1.04(0.89-1.20) | 161.14(138.17-185.79) | 1.21(1.06-1.37) | 0.76(-15.4-20) |
| Cameroon | 75.64(64.56-87.47) | 1.40(1.23-1.60) | 186.10(157.72-218.22) | 1.04(0.92-1.17) | -0.79(-17.06-18.66) |
| Canada | 1988.17(1844.84-2132.56) | 6.22(5.79-6.66) | 6846.06(6233.70-7479.74) | 10.19(9.35-11.03) | 1.84(-14.67-21.55) |
| Central African Republic | 23.03(19.36-27.00) | 1.78(1.52-2.04) | 45.48(38.94-52.60) | 1.69(1.49-1.90) | -0.11(-16.56-19.58) |
| Chad | 39.96(33.70-46.15) | 1.24(1.07-1.41) | 83.08(70.12-97.91) | 1.03(0.91-1.17) | -0.51(-16.82-19) |
| Chile | 884.40(826.49-947.15) | 8.64(8.08-9.22) | 3864.51(3583.26-4144.39) | 15.13(14.03-16.23) | 2.09(-14.39-21.73) |
| China | 18992.59(15850.78-22604.85) | 1.92(1.62-2.26) | 48513.74(41541.45-55949.02) | 2.32(2.03-2.65) | 1.25(-14.7-20.18) |
| Colombia | 364.03(310.70-420.10) | 1.74(1.51-1.97) | 1475.34(1353.06-1605.07) | 2.66(2.44-2.90) | 1.61(-14.79-21.18) |
| Comoros | 3.83(3.26-4.47) | 1.72(1.49-1.96) | 8.67(7.60-9.85) | 1.60(1.42-1.79) | -0.13(-16.45-19.39) |
| Congo | 21.68(18.32-25.06) | 1.84(1.59-2.09) | 63.93(56.50-73.18) | 1.94(1.75-2.15) | 0.15(-16.29-19.81) |
| Cook Islands | 0.47(0.42-0.52) | 3.03(2.76-3.35) | 0.61(0.55-0.67) | 2.80(2.54-3.08) | -0.42(-16.53-18.8) |
| Costa Rica | 92.56(84.71-101.00) | 4.98(4.60-5.43) | 392.42(366.09-419.73) | 7.08(6.62-7.56) | 1(-15.23-20.34) |
| Croatia | 76.64(65.46-89.16) | 1.37(1.17-1.60) | 78.58(71.31-86.67) | 1.50(1.33-1.69) | 0.3(-15.54-19.12) |
| Cuba | 94.94(79.20-112.08) | 0.89(0.75-1.05) | 152.47(134.36-172.95) | 0.93(0.82-1.07) | 0.14(-15.96-19.33) |
| Cyprus | 55.88(48.96-63.25) | 6.72(5.96-7.52) | 142.67(128.29-157.71) | 6.92(6.28-7.59) | -0.02(-16.08-19.12) |
| Czechia | 173.84(152.10-197.12) | 1.45(1.26-1.67) | 446.79(407.89-491.96) | 2.74(2.50-3.01) | 2.61(-13.6-21.86) |
| Côte d'Ivoire | 77.26(64.37-91.93) | 1.39(1.21-1.59) | 173.48(146.91-202.33) | 1.07(0.95-1.20) | -0.74(-16.92-18.58) |
| Democratic People's Republic of Korea | 264.90(226.95-308.30) | 1.44(1.26-1.65) | 492.38(434.51-558.71) | 1.46(1.30-1.64) | -0.13(-15.92-18.61) |
| Democratic Republic of the Congo | 317.55(266.54-369.16) | 1.81(1.55-2.07) | 854.79(736.28-983.76) | 1.90(1.67-2.13) | 0.28(-16.13-19.89) |
| Denmark | 262.97(240.06-287.05) | 3.70(3.37-4.03) | 461.70(424.58-499.76) | 4.58(4.23-4.94) | 0.62(-15.53-19.86) |
| Djibouti | 3.03(2.54-3.56) | 1.66(1.44-1.89) | 13.44(11.68-15.54) | 1.67(1.50-1.86) | 0.14(-16.18-19.65) |
| Dominica | 0.94(0.83-1.06) | 1.58(1.40-1.79) | 1.86(1.69-2.03) | 2.31(2.11-2.51) | 1.36(-14.9-20.72) |
| Dominican Republic | 47.05(38.82-55.87) | 1.01(0.86-1.16) | 147.76(131.89-165.78) | 1.40(1.25-1.56) | 1.44(-14.83-20.81) |
| Ecuador | 347.82(314.82-383.31) | 6.78(6.11-7.48) | 1969.50(1824.15-2122.34) | 12.44(11.52-13.41) | 3.06(-13.62-22.96) |
| Egypt | 652.85(543.23-774.35) | 2.01(1.71-2.30) | 1930.88(1689.59-2222.14) | 2.54(2.25-2.88) | 0.93(-15.15-20.05) |
| El Salvador | 91.48(82.04-101.46) | 2.88(2.60-3.16) | 308.82(283.33-335.08) | 4.90(4.50-5.31) | 2.41(-14.04-22.01) |
| Equatorial Guinea | 3.56(3.00-4.15) | 1.75(1.50-2.01) | 14.94(12.99-17.18) | 2.13(1.89-2.37) | 1.02(-15.47-20.73) |
| Eritrea | 21.85(18.13-25.80) | 1.49(1.29-1.70) | 53.74(46.50-62.13) | 1.47(1.32-1.65) | 0.01(-16.4-19.63) |
| Estonia | 28.31(24.91-31.85) | 1.63(1.43-1.85) | 31.21(28.66-33.91) | 1.86(1.69-2.06) | 0.6(-15.45-19.7) |
| Eswatini | 12.19(10.61-13.96) | 3.65(3.22-4.07) | 20.65(18.47-23.18) | 3.11(2.83-3.42) | -0.54(-16.77-18.87) |
| Ethiopia | 329.69(269.44-391.57) | 1.44(1.21-1.66) | 769.01(653.58-891.12) | 1.45(1.25-1.64) | 0.27(-16.13-19.87) |
| Fiji | 9.88(8.43-11.69) | 1.72(1.52-1.97) | 14.19(12.56-16.04) | 1.62(1.45-1.82) | -0.17(-16.33-19.1) |
| Finland | 245.12(222.22-270.09) | 3.69(3.34-4.07) | 618.90(559.36-679.17) | 5.48(5.01-5.99) | 1.52(-14.76-20.9) |
| France | 2254.69(2047.69-2466.80) | 2.99(2.71-3.30) | 5000.15(4622.62-5367.88) | 4.06(3.76-4.38) | 0.99(-15.26-20.35) |
| Gabon | 11.41(9.83-13.14) | 1.91(1.67-2.17) | 24.58(21.76-27.57) | 2.06(1.85-2.27) | 0.27(-16.12-19.86) |
| Gambia | 6.05(5.06-7.13) | 1.32(1.14-1.51) | 14.13(11.99-16.59) | 1.05(0.93-1.19) | -0.65(-16.75-18.56) |
| Georgia | 136.74(122.79-151.24) | 2.26(2.05-2.52) | 131.35(122.12-140.80) | 2.41(2.23-2.59) | 0.61(-15.23-19.42) |
| Germany | 3559.06(3253.50-3890.67) | 3.12(2.84-3.42) | 6431.50(5956.89-6917.39) | 3.95(3.64-4.26) | 1.13(-15.12-20.5) |
| Ghana | 132.60(114.76-152.26) | 1.78(1.56-2.02) | 336.76(296.55-380.40) | 1.64(1.49-1.81) | -0.18(-16.4-19.19) |
| Greece | 218.04(192.38-245.75) | 1.56(1.38-1.76) | 608.73(548.44-668.53) | 2.99(2.71-3.29) | 2.71(-13.79-22.38) |
| Greenland | 3.88(3.49-4.29) | 10.43(9.36-11.53) | 7.53(6.77-8.29) | 11.12(10.16-12.08) | 0.28(-15.99-19.7) |
| Grenada | 1.14(1.02-1.26) | 1.60(1.43-1.78) | 2.63(2.41-2.87) | 2.32(2.14-2.52) | 1.23(-15.09-20.69) |
| Guam | 8.54(7.91-9.20) | 7.55(7.03-8.08) | 11.10(10.32-11.96) | 6.04(5.67-6.47) | -0.85(-16.88-18.26) |
| Guatemala | 142.60(130.51-155.95) | 3.75(3.49-4.04) | 532.57(498.46-566.26) | 4.68(4.39-4.96) | 1.13(-15.16-20.54) |
| Guinea | 48.62(41.79-56.15) | 1.34(1.16-1.52) | 76.82(65.41-89.98) | 1.05(0.93-1.18) | -0.6(-16.83-18.79) |
| Guinea-Bissau | 6.51(5.50-7.61) | 1.33(1.16-1.52) | 10.95(9.16-13.03) | 0.99(0.87-1.12) | -0.77(-16.94-18.54) |
| Guyana | 8.03(7.02-9.10) | 1.81(1.63-1.99) | 15.63(14.30-16.98) | 2.33(2.15-2.50) | 1.03(-15.23-20.41) |
| Haiti | 67.38(58.72-77.17) | 1.91(1.67-2.14) | 194.70(174.31-217.44) | 2.40(2.17-2.64) | 0.91(-15.42-20.38) |
| Honduras | 77.13(68.17-87.22) | 3.39(3.00-3.79) | 363.23(329.22-399.52) | 5.31(4.81-5.81) | 1.69(-14.68-21.2) |
| Hungary | 286.85(261.70-315.69) | 2.25(2.05-2.49) | 359.88(333.60-385.88) | 2.49(2.29-2.71) | 0.61(-15.28-19.49) |
| Iceland | 7.11(6.41-7.84) | 2.61(2.34-2.88) | 19.94(18.38-21.56) | 3.72(3.44-4.03) | 1.4(-14.85-20.77) |
| India | 29942.62(25233.55-34843.66) | 6.27(5.30-7.21) | 81114.27(70536.27-91985.16) | 6.77(5.91-7.66) | 0.43(-15.88-19.89) |
| Indonesia | 1606.56(1323.97-1918.47) | 1.45(1.22-1.69) | 4551.45(3926.53-5252.11) | 1.77(1.53-2.01) | 0.84(-15.57-20.42) |
| Iran (Islamic Republic of) | 421.45(340.73-505.82) | 1.27(1.05-1.50) | 1282.16(1093.39-1499.90) | 1.48(1.27-1.69) | 0.6(-15.48-19.73) |
| Iraq | 177.33(149.13-209.29) | 1.76(1.52-2.03) | 662.03(586.24-753.25) | 2.19(1.97-2.44) | 0.88(-15.24-20.06) |
| Ireland | 176.94(162.78-192.32) | 4.56(4.20-4.94) | 677.11(616.92-745.07) | 8.91(8.14-9.74) | 2.34(-14.12-21.95) |
| Israel | 122.18(110.48-135.82) | 2.62(2.37-2.90) | 348.23(321.72-374.35) | 3.03(2.79-3.27) | 0.71(-15.5-20.03) |
| Italy | 2172.08(1836.29-2537.96) | 2.89(2.43-3.42) | 5326.34(4613.49-6035.75) | 4.60(4.05-5.19) | 2.04(-14.32-21.52) |
| Jamaica | 20.05(17.43-22.99) | 1.08(0.95-1.24) | 47.14(42.83-51.90) | 1.49(1.36-1.64) | 1.15(-15.09-20.49) |
| Japan | 16812.83(14206.85-19890.91) | 10.05(8.52-11.77) | 35521.07(30753.21-40873.10) | 12.73(11.09-14.49) | 0.7(-15.56-20.1) |
| Jordan | 87.09(78.81-96.41) | 5.37(4.86-5.90) | 540.66(496.31-584.21) | 6.17(5.72-6.62) | 0.42(-15.46-19.28) |
| Kazakhstan | 263.13(231.37-297.65) | 1.91(1.70-2.14) | 497.93(454.42-546.37) | 2.63(2.41-2.88) | 1.4(-14.66-20.48) |
| Kenya | 167.70(139.85-197.96) | 1.67(1.41-1.93) | 476.38(405.36-555.54) | 1.69(1.46-1.92) | -0.02(-16.58-19.83) |
| Kiribati | 1.36(1.22-1.55) | 2.53(2.30-2.79) | 2.47(2.25-2.73) | 2.50(2.31-2.73) | 0.13(-16.18-19.62) |
| Kuwait | 32.50(28.44-37.63) | 3.70(3.37-4.04) | 182.89(163.87-203.74) | 4.94(4.62-5.29) | 1.09(-14.99-20.2) |
| Kyrgyzstan | 51.29(43.89-59.08) | 1.57(1.37-1.78) | 63.52(52.97-74.57) | 1.08(0.93-1.24) | -1.5(-16.96-16.84) |
| Lao People's Democratic Republic | 29.90(25.22-34.82) | 1.39(1.19-1.57) | 80.32(69.94-91.49) | 1.56(1.37-1.73) | 0.52(-15.69-19.83) |
| Latvia | 77.45(69.96-85.98) | 2.53(2.26-2.82) | 36.63(33.42-40.16) | 1.56(1.38-1.75) | -1.17(-16.95-17.61) |
| Lebanon | 50.45(42.82-59.16) | 2.17(1.86-2.50) | 152.43(134.51-171.10) | 2.52(2.24-2.85) | 0.52(-15.5-19.58) |
| Lesotho | 30.10(26.03-34.70) | 3.39(2.95-3.86) | 37.39(33.40-41.74) | 3.11(2.82-3.43) | -0.18(-16.52-19.36) |
| Liberia | 17.06(14.15-20.18) | 1.30(1.11-1.49) | 32.94(27.27-39.46) | 1.12(0.97-1.28) | -0.46(-16.8-19.1) |
| Libya | 52.48(44.56-61.49) | 2.28(1.95-2.63) | 153.65(135.13-175.62) | 2.39(2.12-2.69) | 0.19(-15.78-19.19) |
| Lithuania | 51.77(44.91-59.42) | 1.27(1.10-1.47) | 39.08(35.15-43.20) | 1.14(1.00-1.29) | -0.4(-16.32-18.55) |
| Luxembourg | 13.05(11.86-14.38) | 2.55(2.32-2.82) | 34.75(32.12-37.53) | 3.52(3.25-3.82) | 1.17(-15.07-20.5) |
| Madagascar | 105.40(89.54-122.34) | 1.85(1.62-2.10) | 273.26(237.46-311.23) | 1.99(1.77-2.22) | 0.28(-16.17-19.97) |
| Malawi | 62.89(52.06-74.06) | 1.36(1.17-1.55) | 132.39(113.38-153.55) | 1.36(1.21-1.53) | 0.2(-16.27-19.91) |
| Malaysia | 186.60(164.50-211.88) | 1.80(1.62-2.03) | 699.40(633.19-773.41) | 2.34(2.12-2.57) | 0.75(-15.43-20.02) |
| Maldives | 8.75(7.76-9.80) | 8.54(7.69-9.40) | 46.41(42.50-50.40) | 11.07(10.20-11.98) | 0.8(-15.44-20.17) |
| Mali | 66.17(56.35-77.03) | 1.36(1.17-1.56) | 148.66(129.26-169.88) | 1.21(1.08-1.35) | -0.22(-16.55-19.31) |
| Malta | 20.36(18.69-22.05) | 4.80(4.42-5.20) | 65.57(60.18-71.31) | 7.46(6.94-7.99) | 1.54(-14.75-20.94) |
| Marshall Islands | 0.91(0.81-1.02) | 3.46(3.15-3.82) | 1.66(1.52-1.82) | 3.54(3.25-3.84) | 0.17(-16.06-19.54) |
| Mauritania | 15.29(13.05-17.71) | 1.32(1.15-1.51) | 28.40(24.78-32.64) | 1.06(0.95-1.19) | -0.71(-16.82-18.52) |
| Mauritius | 40.69(36.77-44.94) | 5.43(4.92-5.95) | 200.15(184.60-216.48) | 10.66(9.91-11.41) | 2.35(-14.11-21.95) |
| Mexico | 2269.99(1927.21-2643.23) | 4.74(4.04-5.50) | 7794.88(6842.62-8766.00) | 6.13(5.37-6.86) | 0.94(-15.3-20.29) |
| Micronesia (Federated States of) | 2.26(2.04-2.51) | 3.29(3.00-3.63) | 3.11(2.82-3.41) | 3.35(3.09-3.64) | 0.17(-16.08-19.56) |
| Monaco | 2.18(1.93-2.43) | 3.73(3.34-4.16) | 3.37(3.06-3.70) | 4.15(3.78-4.52) | 0.37(-15.74-19.56) |
| Mongolia | 38.64(34.21-42.93) | 3.45(3.10-3.82) | 79.34(71.67-87.68) | 3.32(3.03-3.65) | -0.23(-15.92-18.39) |
| Montenegro | 7.98(6.71-9.35) | 1.22(1.04-1.43) | 8.40(7.38-9.59) | 1.12(0.97-1.30) | -0.25(-16.01-18.46) |
| Morocco | 334.93(282.92-392.10) | 2.06(1.77-2.37) | 911.34(810.06-1027.40) | 2.51(2.23-2.81) | 0.87(-15.33-20.17) |
| Mozambique | 86.68(72.02-102.62) | 1.26(1.07-1.45) | 177.49(149.10-208.24) | 1.20(1.05-1.37) | -0.03(-16.31-19.43) |
| Myanmar | 431.98(370.11-498.19) | 1.81(1.56-2.05) | 1315.20(1166.56-1481.91) | 2.57(2.29-2.88) | 1.4(-14.81-20.7) |
| Namibia | 26.17(22.82-29.77) | 3.71(3.27-4.16) | 55.47(49.91-61.36) | 3.51(3.21-3.83) | -0.2(-16.49-19.28) |
| Nauru | 0.27(0.24-0.30) | 3.78(3.44-4.17) | 0.31(0.28-0.34) | 3.61(3.33-3.90) | -0.15(-16.27-19.08) |
| Nepal | 631.47(558.99-713.33) | 6.56(5.84-7.37) | 2034.64(1852.21-2214.10) | 8.55(7.79-9.28) | 0.81(-15.45-20.21) |
| Netherlands | 413.82(369.68-462.39) | 2.22(1.99-2.50) | 1393.95(1256.22-1537.54) | 4.41(4.00-4.82) | 2.25(-14.22-21.88) |
| New Zealand | 165.75(139.65-194.52) | 4.29(3.64-5.00) | 466.91(404.24-531.50) | 5.59(4.88-6.31) | 0.99(-15.38-20.53) |
| Nicaragua | 38.62(33.23-44.48) | 2.14(1.88-2.42) | 184.42(167.90-200.61) | 3.64(3.31-3.97) | 2.33(-13.99-21.75) |
| Niger | 47.64(39.94-56.33) | 1.32(1.14-1.51) | 110.41(92.97-130.61) | 0.97(0.85-1.10) | -0.81(-17.08-18.66) |
| Nigeria | 711.63(583.60-844.39) | 1.37(1.14-1.60) | 1381.63(1159.89-1644.76) | 1.13(0.97-1.30) | -0.31(-16.63-19.21) |
| Niue | 0.07(0.07-0.08) | 3.54(3.23-3.88) | 0.07(0.06-0.07) | 3.45(3.16-3.76) | -0.1(-16.23-19.15) |
| North Macedonia | 25.33(21.40-29.66) | 1.24(1.06-1.45) | 30.93(27.02-35.36) | 1.13(0.98-1.30) | -0.26(-16.06-18.51) |
| Northern Mariana Islands | 1.54(1.32-1.78) | 4.38(3.87-4.93) | 2.35(2.08-2.63) | 4.28(3.88-4.70) | -0.37(-16.49-18.87) |
| Norway | 244.04(205.11-285.71) | 4.31(3.66-5.01) | 473.81(406.35-539.10) | 5.31(4.62-6.02) | 0.96(-15.28-20.3) |
| Oman | 20.47(17.11-24.48) | 1.94(1.68-2.22) | 95.27(81.68-111.86) | 2.92(2.62-3.26) | 1.56(-14.57-20.75) |
| Pakistan | 2886.68(2439.57-3340.84) | 4.93(4.19-5.71) | 5642.69(4933.99-6381.06) | 4.43(3.87-5.02) | -0.26(-16.48-19.1) |
| Palau | 0.52(0.47-0.58) | 3.92(3.57-4.30) | 0.80(0.72-0.88) | 3.73(3.44-4.05) | -0.17(-16.29-19.07) |
| Palestine | 66.37(59.54-73.27) | 6.91(6.25-7.61) | 275.85(255.02-297.13) | 8.84(8.13-9.49) | 0.96(-15.14-20.12) |
| Panama | 42.17(37.97-46.96) | 2.57(2.33-2.84) | 215.62(199.75-231.79) | 4.85(4.50-5.22) | 2.47(-14-22.09) |
| Papua New Guinea | 104.49(92.82-117.63) | 4.19(3.81-4.61) | 333.89(306.64-363.14) | 4.82(4.48-5.19) | 0.59(-15.79-20.16) |
| Paraguay | 35.82(31.47-40.92) | 1.48(1.32-1.66) | 126.99(115.97-139.11) | 2.13(1.95-2.32) | 1.74(-14.78-21.45) |
| Peru | 1722.68(1573.72-1878.85) | 14.63(13.29-15.98) | 8297.41(7814.94-8782.77) | 24.73(23.23-26.22) | 2.42(-14.19-22.23) |
| Philippines | 335.90(272.26-407.63) | 0.96(0.80-1.13) | 675.64(555.88-810.42) | 0.73(0.61-0.85) | -0.84(-16.93-18.36) |
| Poland | 1066.65(897.13-1248.99) | 2.57(2.18-3.01) | 1337.37(1181.95-1519.73) | 2.55(2.26-2.89) | 0.27(-15.6-19.13) |
| Portugal | 349.43(307.93-394.92) | 2.66(2.36-2.98) | 1047.88(961.79-1129.22) | 4.86(4.50-5.24) | 2.15(-14.2-21.6) |
| Puerto Rico | 90.49(82.21-99.47) | 2.53(2.30-2.77) | 254.76(232.27-277.07) | 4.06(3.73-4.40) | 1.58(-14.76-21.07) |
| Qatar | 6.69(5.52-8.10) | 2.44(2.15-2.74) | 58.33(48.37-70.38) | 2.42(2.19-2.68) | -0.12(-15.99-18.75) |
| Republic of Korea | 1903.56(1696.06-2159.51) | 5.03(4.55-5.59) | 7893.22(7171.40-8673.49) | 8.99(8.21-9.83) | 2.49(-13.96-22.07) |
| Republic of Moldova | 52.65(43.86-62.52) | 1.17(0.99-1.38) | 44.94(38.83-51.89) | 1.02(0.88-1.19) | -0.34(-16.25-18.58) |
| Romania | 1337.17(1209.00-1483.24) | 5.06(4.59-5.53) | 1004.84(916.19-1095.49) | 3.44(3.15-3.74) | -1.12(-16.77-17.47) |
| Russian Federation | 2484.59(2057.36-2916.77) | 1.48(1.23-1.74) | 1706.51(1445.70-1984.73) | 0.96(0.81-1.13) | -1.61(-17.41-17.2) |
| Rwanda | 54.45(45.82-63.83) | 1.67(1.44-1.91) | 129.77(111.71-148.86) | 1.67(1.48-1.88) | 0.18(-16.17-19.71) |
| Saint Kitts and Nevis | 0.76(0.69-0.85) | 2.16(1.96-2.37) | 2.35(2.14-2.57) | 3.32(3.07-3.57) | 1.58(-14.76-21.04) |
| Saint Lucia | 2.01(1.80-2.24) | 2.22(2.03-2.45) | 7.77(7.24-8.30) | 3.36(3.14-3.59) | 1.34(-14.97-20.76) |
| Saint Vincent and the Grenadines | 0.88(0.76-1.03) | 1.16(1.00-1.33) | 2.45(2.25-2.67) | 1.80(1.65-1.96) | 1.59(-14.72-21.03) |
| Samoa | 4.01(3.60-4.49) | 3.43(3.14-3.77) | 6.03(5.53-6.56) | 3.48(3.22-3.76) | 0.03(-16.14-19.32) |
| San Marino | 0.53(0.47-0.60) | 1.72(1.49-1.96) | 0.93(0.83-1.04) | 1.58(1.39-1.78) | -0.22(-16.24-18.85) |
| Sao Tome and Principe | 1.59(1.40-1.81) | 2.32(2.05-2.61) | 3.54(3.20-3.94) | 2.50(2.29-2.76) | 0.46(-15.8-19.85) |
| Saudi Arabia | 404.69(363.10-449.21) | 5.95(5.33-6.58) | 2493.89(2265.72-2737.07) | 9.52(8.73-10.31) | 1.71(-14.51-21.02) |
| Senegal | 52.87(45.05-61.33) | 1.39(1.21-1.58) | 109.06(93.76-125.95) | 1.12(0.99-1.25) | -0.47(-16.64-18.85) |
| Serbia | 150.89(131.55-172.68) | 1.37(1.20-1.58) | 179.77(163.67-198.16) | 1.45(1.30-1.63) | 0.61(-15.3-19.51) |
| Seychelles | 0.77(0.65-0.88) | 1.35(1.15-1.55) | 1.90(1.66-2.16) | 1.57(1.39-1.76) | 0.32(-15.78-19.5) |
| Sierra Leone | 30.13(25.26-35.01) | 1.26(1.09-1.44) | 51.21(43.15-60.41) | 0.98(0.86-1.12) | -0.62(-16.85-18.78) |
| Singapore | 85.59(74.75-98.52) | 2.97(2.64-3.36) | 330.91(300.84-362.51) | 3.96(3.62-4.32) | 1.17(-15.05-20.49) |
| Slovakia | 98.54(84.95-113.21) | 1.74(1.51-2.00) | 155.52(141.53-169.89) | 2.08(1.88-2.31) | 0.83(-15.1-19.75) |
| Slovenia | 37.47(33.09-42.50) | 1.64(1.44-1.88) | 90.15(81.50-99.48) | 2.67(2.40-2.97) | 2.15(-14.01-21.34) |
| Solomon Islands | 5.14(4.47-5.90) | 2.58(2.31-2.89) | 12.56(11.31-14.04) | 2.58(2.37-2.82) | 0.09(-16.21-19.57) |
| Somalia | 51.33(42.39-60.71) | 1.66(1.42-1.90) | 123.16(102.86-144.87) | 1.45(1.27-1.64) | -0.32(-16.72-19.32) |
| South Africa | 1184.41(1012.05-1371.62) | 5.29(4.50-6.09) | 2233.52(1936.54-2540.81) | 4.61(4.01-5.21) | -0.46(-16.9-19.23) |
| South Sudan | 50.03(42.66-58.57) | 1.74(1.51-1.98) | 68.73(59.40-79.17) | 1.50(1.33-1.67) | -0.47(-16.89-19.19) |
| Spain | 2474.18(2284.63-2677.61) | 4.78(4.41-5.15) | 6552.96(5964.09-7135.55) | 7.26(6.66-7.88) | 1.55(-14.74-20.96) |
| Sri Lanka | 166.50(144.56-191.33) | 1.41(1.25-1.59) | 533.68(477.96-590.89) | 2.02(1.82-2.23) | 1.36(-14.94-20.79) |
| Sudan | 218.61(183.10-259.00) | 2.02(1.72-2.35) | 591.74(515.40-680.42) | 2.30(2.05-2.57) | 0.58(-15.57-19.81) |
| Suriname | 5.28(4.68-5.93) | 1.89(1.69-2.11) | 15.87(14.53-17.19) | 2.54(2.33-2.75) | 1.15(-15.11-20.54) |
| Sweden | 536.49(450.52-625.78) | 4.25(3.59-4.98) | 1096.57(939.64-1257.92) | 5.64(4.89-6.38) | 0.94(-15.26-20.23) |
| Switzerland | 309.72(284.53-337.47) | 3.30(3.01-3.62) | 621.66(570.30-669.31) | 3.90(3.59-4.21) | 0.65(-15.52-19.92) |
| Syrian Arab Republic | 153.04(131.20-177.29) | 2.46(2.14-2.79) | 422.08(375.82-475.26) | 3.09(2.79-3.42) | 0.73(-15.33-19.82) |
| Taiwan (Province of China) | 205.41(177.70-236.72) | 1.17(1.02-1.34) | 1024.77(924.14-1131.59) | 2.50(2.27-2.76) | 2.67(-13.51-21.88) |
| Tajikistan | 207.66(186.80-228.89) | 7.29(6.52-8.04) | 508.67(467.35-552.26) | 8.78(8.02-9.55) | 1.03(-14.89-19.93) |
| Thailand | 424.10(356.40-501.55) | 1.02(0.88-1.17) | 992.31(862.98-1127.47) | 0.96(0.84-1.10) | -0.3(-16.54-19.09) |
| Timor-Leste | 4.87(4.06-5.72) | 1.43(1.24-1.64) | 14.05(12.42-15.92) | 1.60(1.42-1.79) | 0.58(-15.65-19.93) |
| Togo | 21.26(17.78-24.94) | 1.28(1.12-1.46) | 52.04(44.15-60.69) | 0.97(0.85-1.11) | -0.8(-17.04-18.63) |
| Tokelau | 0.05(0.04-0.05) | 3.39(3.08-3.72) | 0.05(0.04-0.05) | 3.27(3.01-3.57) | -0.12(-16.26-19.12) |
| Tonga | 1.77(1.56-1.99) | 2.55(2.30-2.84) | 2.39(2.19-2.61) | 2.66(2.45-2.90) | 0.04(-16.15-19.35) |
| Trinidad and Tobago | 22.02(19.89-24.42) | 2.51(2.30-2.75) | 56.61(52.29-61.43) | 3.06(2.85-3.31) | 1.06(-15.15-20.37) |
| Tunisia | 118.31(99.95-138.18) | 2.12(1.82-2.45) | 334.52(294.79-377.28) | 2.50(2.21-2.80) | 0.54(-15.49-19.6) |
| Turkey | 935.54(818.73-1066.02) | 2.33(2.05-2.62) | 2909.15(2641.98-3190.33) | 3.05(2.79-3.36) | 1.29(-15.05-20.77) |
| Turkmenistan | 51.15(45.07-58.15) | 2.35(2.10-2.61) | 58.10(49.74-67.26) | 1.27(1.11-1.45) | -1.9(-17.35-16.43) |
| Tuvalu | 0.26(0.23-0.29) | 3.33(3.02-3.66) | 0.40(0.37-0.44) | 3.47(3.20-3.75) | 0.16(-16.03-19.48) |
| Uganda | 127.26(108.13-149.27) | 1.71(1.47-1.95) | 321.93(277.62-370.11) | 1.67(1.48-1.86) | 0.06(-16.41-19.77) |
| Ukraine | 2215.60(1879.91-2580.20) | 3.50(3.01-4.07) | 668.34(574.29-770.12) | 1.17(1.01-1.36) | -4.15(-19.51-14.15) |
| United Arab Emirates | 31.12(25.95-37.69) | 2.97(2.56-3.39) | 350.70(290.74-416.18) | 3.33(2.95-3.68) | 0.33(-15.63-19.29) |
| United Kingdom | 4850.81(4160.48-5568.42) | 6.16(5.33-7.03) | 11429.54(9978.87-12861.24) | 9.40(8.28-10.53) | 1.63(-14.7-21.08) |
| United Republic of Tanzania | 165.93(138.50-194.32) | 1.30(1.12-1.48) | 443.05(381.88-512.81) | 1.38(1.21-1.54) | 0.5(-15.93-20.13) |
| United States of America | 26340.79(22647.09-30364.39) | 8.73(7.53-10.05) | 59754.49(52045.56-67737.27) | 11.04(9.76-12.35) | 0.88(-15.48-20.4) |
| United States Virgin Islands | 2.68(2.41-2.98) | 3.05(2.76-3.36) | 7.38(6.76-8.07) | 4.55(4.21-4.90) | 1.51(-14.8-20.94) |
| Uruguay | 142.25(125.77-159.39) | 3.87(3.43-4.32) | 307.07(286.28-328.83) | 5.83(5.44-6.26) | 1.53(-14.87-21.08) |
| Uzbekistan | 657.28(595.07-726.44) | 5.23(4.73-5.72) | 1036.30(955.06-1129.56) | 3.80(3.53-4.08) | -1.33(-16.8-17.02) |
| Vanuatu | 3.06(2.70-3.47) | 3.28(2.97-3.64) | 7.54(6.88-8.21) | 3.13(2.90-3.38) | -0.08(-16.32-19.31) |
| Venezuela (Bolivarian Republic of) | 265.64(235.66-299.17) | 2.40(2.16-2.64) | 904.97(839.33-975.10) | 3.00(2.78-3.23) | 0.79(-15.41-20.09) |
| Viet Nam | 544.28(457.00-638.21) | 1.30(1.11-1.49) | 1958.11(1707.45-2212.28) | 1.86(1.64-2.09) | 1.27(-14.92-20.54) |
| Yemen | 119.50(98.53-141.13) | 1.97(1.67-2.27) | 406.58(349.16-471.61) | 2.20(1.94-2.45) | 0.66(-15.56-19.99) |
| Zambia | 52.43(44.12-61.33) | 1.54(1.34-1.75) | 148.19(127.67-170.77) | 1.59(1.41-1.77) | 0.28(-16.2-20) |
| Zimbabwe | 105.06(88.12-123.85) | 2.02(1.76-2.30) | 167.37(142.79-196.01) | 1.87(1.65-2.09) | -0.24(-16.5-19.19) |

ILD&PS, Interstitial lung disease and pulmonary sarcoidosis; SDI, Socio-demographic index; ASIR, Age-standardized incidence rate; EAPC, Estimated annual percentage change; UI, Uncertainty interval; CI, Confidence interval.

**Table S8. Number and age-standardized DALYs of ILD&PS, 1990 vs. 2021 (Nations).**

| **Location** | **Number in 1990  (95% UI)** | **ASDR in 1990  (per 100 000, 95% UI)** | **Number in 2021  (95% UI)** | **ASDR in 2021  (per 100 000, 95% UI)** | **EAPC of ASDR (95% CI)** |
| --- | --- | --- | --- | --- | --- |
| Afghanistan | 245.64(96.06-567.45) | 3.46(1.35-7.81) | 985.16(297.22-2843.89) | 7.95(2.17-22.00) | 3.52(3.17-3.86) |
| Albania | 662.48(450.91-963.04) | 29.60(19.93-43.33) | 881.93(503.93-1438.15) | 21.67(12.41-35.79) | -1.01(-1.16--0.86) |
| Algeria | 489.33(241.64-823.64) | 3.44(1.70-5.87) | 2775.79(1103.45-6567.92) | 7.42(2.82-18.15) | 3.1(2.85-3.35) |
| American Samoa | 15.88(9.80-21.35) | 43.72(26.94-58.06) | 14.66(10.45-23.51) | 30.06(21.39-48.08) | -1.27(-1.46--1.09) |
| Andorra | 27.98(17.51-42.85) | 48.95(30.93-75.28) | 65.32(27.62-115.22) | 43.43(18.51-75.96) | -0.08(-0.25-0.1) |
| Angola | 1328.23(442.45-2393.60) | 29.07(10.78-56.23) | 3369.45(1415.04-5840.85) | 24.94(10.37-45.62) | -0.63(-0.68--0.58) |
| Antigua and Barbuda | 5.59(5.04-6.22) | 10.07(9.08-11.26) | 23.50(21.90-25.41) | 23.04(21.53-24.89) | 3.28(2.97-3.59) |
| Argentina | 18269.29(16720.53-19842.62) | 56.82(52.01-61.73) | 41401.62(38080.12-45001.05) | 73.84(67.96-80.08) | 1.13(0.8-1.47) |
| Armenia | 1713.21(1553.09-1896.79) | 58.61(53.59-64.52) | 1269.90(1124.06-1441.76) | 31.00(27.47-35.14) | -0.9(-1.83-0.04) |
| Australia | 4583.15(4179.70-4988.41) | 23.27(21.20-25.33) | 28209.26(24680.04-30789.53) | 60.65(53.78-65.68) | 3.27(2.65-3.9) |
| Austria | 1763.79(1552.78-1977.01) | 15.55(13.57-17.67) | 4781.06(4287.92-5266.64) | 27.32(24.71-30.17) | 2.64(2.37-2.92) |
| Azerbaijan | 1893.56(1074.12-3050.61) | 34.84(19.04-58.10) | 2359.99(1268.58-4097.31) | 22.43(12.21-37.20) | -1.76(-2.1--1.42) |
| Bahamas | 54.74(49.36-60.34) | 31.36(28.28-34.69) | 274.54(220.63-341.18) | 67.21(54.40-82.97) | 2.98(2.72-3.24) |
| Bahrain | 96.05(67.50-159.47) | 50.47(35.70-86.94) | 537.23(331.49-740.96) | 62.07(38.72-86.10) | 0.84(0.56-1.12) |
| Bangladesh | 37317.79(21043.38-58255.85) | 74.87(42.55-117.45) | 88724.51(61324.46-129963.51) | 63.13(43.44-92.23) | -0.64(-0.75--0.52) |
| Barbados | 58.28(52.52-63.45) | 20.10(18.18-22.00) | 195.60(159.04-234.74) | 40.22(32.68-48.47) | 3.03(2.72-3.34) |
| Belarus | 5148.82(4380.17-5844.67) | 41.50(35.29-47.52) | 1286.53(1061.68-1526.65) | 9.01(7.42-10.69) | -5.85(-6.25--5.45) |
| Belgium | 3474.77(3190.28-3741.67) | 22.93(21.05-24.66) | 10294.98(9351.66-11176.49) | 45.77(42.03-49.52) | 2.87(2.53-3.21) |
| Belize | 63.05(54.96-72.88) | 55.24(47.42-65.35) | 404.36(354.20-463.64) | 119.55(104.47-136.90) | 2.89(2.49-3.3) |
| Benin | 1007.62(373.69-1653.67) | 45.23(17.41-76.93) | 1888.20(742.61-3703.88) | 31.16(12.10-65.22) | -1.14(-1.4--0.89) |
| Bermuda | 38.29(32.75-44.71) | 61.79(52.96-71.94) | 103.01(87.92-122.32) | 81.37(68.97-96.41) | 1.16(0.94-1.37) |
| Bhutan | 171.90(100.38-257.47) | 67.63(40.71-99.89) | 421.26(280.27-638.10) | 69.83(46.40-105.74) | 0.01(-0.05-0.08) |
| Bolivia (Plurinational State of) | 5438.80(2678.51-10908.72) | 165.25(83.42-319.27) | 15874.02(10498.49-22568.23) | 184.19(121.94-261.47) | 0.54(0.46-0.61) |
| Bosnia and Herzegovina | 975.64(655.15-1420.79) | 23.14(15.30-33.83) | 883.64(568.65-1362.58) | 15.78(10.36-24.03) | -1.4(-1.51--1.3) |
| Botswana | 266.52(80.70-542.14) | 46.42(13.81-94.28) | 489.41(168.71-954.93) | 32.77(11.23-64.80) | -1.08(-1.21--0.95) |
| Brazil | 27149.87(25670.49-28866.95) | 26.54(24.96-28.31) | 100994.13(93922.12-106357.07) | 40.84(37.92-43.04) | 1.33(1.05-1.6) |
| Brunei Darussalam | 86.35(61.13-116.37) | 79.55(56.11-106.73) | 206.76(152.14-268.25) | 60.90(43.88-79.94) | -0.79(-0.88--0.69) |
| Bulgaria | 1520.72(1317.53-1714.20) | 13.13(11.52-14.76) | 1713.27(1450.08-2013.19) | 14.39(12.18-16.94) | 0.31(0.11-0.51) |
| Burkina Faso | 1370.65(432.38-2322.76) | 28.18(9.76-46.49) | 2430.07(945.17-4139.96) | 21.84(8.89-40.01) | -0.86(-0.99--0.72) |
| Burundi | 872.66(248.06-1563.39) | 30.30(10.03-53.06) | 1425.81(560.78-2817.25) | 24.48(9.20-50.04) | -0.9(-1.04--0.76) |
| Cabo Verde | 137.69(42.01-278.74) | 58.19(17.61-118.16) | 115.97(48.31-221.61) | 25.03(10.34-47.29) | -2.43(-3.09--1.77) |
| Cambodia | 230.68(68.89-680.18) | 4.54(1.35-13.04) | 674.21(248.77-1643.78) | 5.27(1.90-13.20) | 0.6(0.54-0.66) |
| Cameroon | 2588.48(988.13-4218.69) | 53.02(20.89-89.53) | 5502.12(2217.29-9768.31) | 36.77(14.63-67.23) | -1.1(-1.32--0.88) |
| Canada | 16071.14(14744.49-17578.96) | 49.71(45.66-54.49) | 57701.30(52031.99-62693.14) | 80.23(73.05-86.95) | 1.94(1.69-2.2) |
| Central African Republic | 438.36(134.31-858.16) | 35.46(12.24-67.98) | 842.12(300.94-1631.48) | 33.74(12.83-69.87) | -0.22(-0.29--0.15) |
| Chad | 1259.18(438.96-2178.61) | 41.30(14.79-72.24) | 2410.74(991.76-4285.01) | 35.39(14.63-65.23) | -0.36(-0.54--0.18) |
| Chile | 8497.52(7974.81-8997.94) | 84.29(79.01-89.34) | 41509.14(38123.62-44276.21) | 161.71(148.71-172.35) | 2.29(1.98-2.6) |
| China | 112641.81(83845.24-167128.83) | 12.55(9.49-18.57) | 222288.42(158550.51-288300.69) | 10.81(7.70-13.97) | -0.23(-0.38--0.08) |
| Colombia | 2777.92(2556.22-3007.61) | 13.53(12.39-14.68) | 19221.14(16296.32-22708.96) | 35.07(29.71-41.44) | 3.08(2.65-3.5) |
| Comoros | 64.83(21.12-121.23) | 27.90(9.62-52.83) | 116.04(48.74-236.14) | 22.00(9.15-45.41) | -0.96(-1.19--0.73) |
| Congo | 432.21(141.04-854.89) | 37.65(12.97-78.71) | 950.07(397.61-1683.54) | 31.68(13.25-58.93) | -0.72(-0.81--0.63) |
| Cook Islands | 7.17(5.05-11.27) | 44.85(31.56-71.65) | 6.58(3.73-11.99) | 31.24(17.27-56.50) | -1.37(-1.49--1.24) |
| Costa Rica | 918.14(848.21-990.38) | 48.22(44.26-52.15) | 4207.88(3768.41-4663.18) | 77.17(69.18-85.37) | 1.73(1.44-2.02) |
| Croatia | 2206.14(792.67-3731.95) | 48.11(18.06-83.20) | 4415.84(1664.38-8190.02) | 33.59(12.55-64.16) | -1.06(-1.29--0.83) |
| Cuba | 553.28(479.02-628.13) | 9.38(8.17-10.63) | 793.12(680.07-903.35) | 10.45(8.98-12.00) | 0.78(0.5-1.05) |
| Cyprus | 577.88(512.50-647.57) | 5.54(4.93-6.20) | 1607.91(1386.48-1820.75) | 9.05(7.82-10.28) | 1.96(1.71-2.21) |
| Czechia | 933.49(625.19-1409.67) | 131.37(90.08-197.63) | 1666.46(1199.09-2195.78) | 81.34(59.68-106.60) | -1.4(-1.59--1.21) |
| Côte d'Ivoire | 2839.21(2537.47-3179.53) | 22.31(20.06-24.79) | 7627.85(6618.09-8640.07) | 37.61(32.57-42.63) | 2.6(2.3-2.89) |
| Democratic People's Republic of Korea | 1715.99(957.48-3155.23) | 10.40(5.85-19.24) | 3649.90(2076.00-6683.08) | 11.26(6.51-20.35) | 0.45(0.36-0.55) |
| Democratic Republic of the Congo | 5632.02(2050.69-11528.37) | 33.02(12.29-73.75) | 14919.72(5881.98-35891.06) | 37.12(14.13-97.53) | 0.4(0.27-0.53) |
| Denmark | 2099.40(1920.48-2304.62) | 27.22(24.90-29.89) | 5891.30(5287.17-6537.58) | 50.09(45.44-55.08) | 2.27(2.06-2.49) |
| Djibouti | 39.03(13.42-69.79) | 22.85(8.60-40.11) | 133.10(53.81-271.92) | 18.40(7.42-36.50) | -0.8(-0.91--0.69) |
| Dominica | 10.68(8.09-15.75) | 17.70(13.45-26.37) | 21.85(14.39-29.93) | 27.23(18.04-37.72) | 1.59(1.46-1.72) |
| Dominican Republic | 390.65(255.42-676.82) | 8.76(5.61-16.12) | 1371.04(813.11-2304.83) | 13.22(7.81-22.07) | 1.68(1.52-1.85) |
| Ecuador | 3375.27(3073.48-3726.43) | 62.34(56.34-69.21) | 23486.70(19334.40-28546.61) | 147.19(121.71-177.96) | 3.9(3.44-4.37) |
| Egypt | 5124.18(3771.70-7073.28) | 17.23(13.04-23.96) | 8173.55(6084.27-10662.44) | 12.42(9.39-16.42) | -1.41(-1.68--1.15) |
| El Salvador | 1241.69(971.04-1812.02) | 37.40(29.14-54.07) | 2828.87(1890.44-3717.82) | 45.22(30.25-59.36) | 0.97(0.81-1.14) |
| Equatorial Guinea | 69.77(22.32-139.04) | 33.32(11.51-62.63) | 168.31(65.28-341.93) | 27.55(11.03-56.55) | -0.7(-0.77--0.64) |
| Eritrea | 437.66(113.14-864.75) | 29.04(9.06-52.15) | 863.22(343.13-1448.27) | 25.35(10.48-42.28) | -0.53(-0.61--0.44) |
| Estonia | 1204.05(1055.60-1380.17) | 59.71(52.29-68.24) | 222.18(188.69-263.94) | 9.09(7.52-10.94) | -7.13(-8.8--5.42) |
| Eswatini | 146.52(44.49-288.39) | 47.16(14.29-93.99) | 254.79(84.21-494.99) | 41.75(13.90-79.39) | -0.24(-0.43--0.06) |
| Ethiopia | 5418.61(1473.09-9656.82) | 22.80(6.90-39.47) | 9411.71(3682.94-18421.03) | 18.25(6.71-37.18) | -0.99(-1.09--0.89) |
| Fiji | 138.09(103.08-185.42) | 23.43(17.46-31.05) | 208.47(130.81-288.25) | 24.13(15.12-33.14) | 0.22(0.11-0.32) |
| Finland | 2134.04(1887.19-2409.64) | 30.19(26.63-34.23) | 7656.96(6780.97-8399.03) | 59.88(54.11-65.52) | 2.83(2.49-3.17) |
| France | 18766.52(17098.82-20510.06) | 23.07(21.03-25.15) | 54167.11(47666.66-60294.71) | 39.23(35.16-43.40) | 1.96(1.72-2.21) |
| Gabon | 196.48(70.80-347.99) | 33.50(12.44-60.22) | 294.87(119.27-607.85) | 26.82(10.86-55.94) | -0.83(-0.89--0.77) |
| Gambia | 192.02(66.72-329.34) | 47.67(17.26-84.54) | 438.03(185.00-848.44) | 38.46(15.79-76.36) | -0.69(-0.99--0.39) |
| Georgia | 1000.27(847.17-1191.43) | 16.49(14.00-19.63) | 850.18(724.31-984.85) | 15.21(12.98-17.64) | 1.21(0.32-2.12) |
| Germany | 35060.86(31245.18-39241.59) | 28.69(25.55-32.07) | 79027.35(71415.10-85689.30) | 41.50(38.20-44.56) | 1.71(1.55-1.86) |
| Ghana | 2385.63(878.42-3939.72) | 33.51(12.93-53.64) | 6346.31(2800.10-10446.55) | 33.89(14.89-55.11) | 0.2(0.11-0.3) |
| Greece | 1531.58(1404.99-1680.62) | 10.73(9.87-11.72) | 9475.43(8534.98-10294.73) | 41.76(38.48-44.91) | 5.3(4.67-5.94) |
| Greenland | 35.94(24.20-46.41) | 107.78(66.11-145.04) | 55.94(30.69-78.31) | 85.04(46.00-118.85) | -0.47(-0.63--0.31) |
| Grenada | 12.69(10.87-14.80) | 17.59(15.06-20.63) | 43.00(37.00-49.03) | 38.81(33.46-44.03) | 3.09(2.78-3.41) |
| Guam | 149.09(112.26-179.95) | 140.54(106.06-171.54) | 145.04(118.88-198.89) | 79.60(65.45-108.96) | -1.35(-1.53--1.17) |
| Guatemala | 2433.52(2031.38-2968.43) | 58.26(50.11-68.79) | 8716.41(7465.27-10003.52) | 75.00(64.14-86.27) | 0.86(0.41-1.32) |
| Guinea | 1501.59(563.97-2454.03) | 42.70(16.73-70.61) | 2208.24(920.17-4240.41) | 34.76(14.28-70.17) | -0.49(-0.69--0.3) |
| Guinea-Bissau | 260.41(75.70-511.84) | 58.02(18.36-108.36) | 362.30(144.18-612.79) | 40.53(16.88-69.97) | -1(-1.23--0.76) |
| Guyana | 145.88(128.80-163.29) | 34.50(30.33-38.76) | 320.64(241.29-413.35) | 47.16(35.88-60.39) | 2.2(1.45-2.96) |
| Haiti | 1503.89(510.35-3043.52) | 39.07(15.11-67.89) | 4163.75(1715.27-8003.72) | 49.35(22.24-90.36) | 0.99(0.89-1.08) |
| Honduras | 1593.37(1050.36-2504.24) | 59.67(40.44-90.75) | 5757.87(3205.61-8487.86) | 83.78(45.77-121.53) | 1.27(1.12-1.41) |
| Hungary | 3905.38(3622.90-4229.90) | 28.63(26.49-31.00) | 5916.98(5194.60-6670.17) | 34.33(30.03-38.71) | 1.08(0.79-1.37) |
| Iceland | 45.77(41.44-50.82) | 16.04(14.54-17.88) | 233.95(208.21-258.44) | 41.27(36.96-45.40) | 3.69(3.32-4.06) |
| India | 392464.72(218814.51-615381.97) | 85.19(48.25-131.89) | 1124247.84(750835.18-1523498.82) | 95.50(63.70-129.59) | 0.49(0.41-0.58) |
| Indonesia | 7739.48(2463.05-20083.53) | 6.78(2.14-17.42) | 20228.64(7241.74-47750.40) | 7.94(2.79-19.06) | 0.58(0.52-0.63) |
| Iran (Islamic Republic of) | 710.00(458.81-998.11) | 2.30(1.49-3.18) | 2571.14(1498.31-3631.14) | 3.00(1.74-4.26) | 1.19(1.06-1.31) |
| Iraq | 1561.70(1123.23-2128.65) | 16.92(11.90-23.64) | 5105.83(3393.72-7065.11) | 19.64(13.05-26.71) | 0.43(0.34-0.52) |
| Ireland | 1923.42(1786.80-2071.29) | 46.77(43.52-50.41) | 7434.98(6599.22-8251.17) | 93.78(83.81-103.76) | 3.01(2.61-3.41) |
| Israel | 1049.85(945.31-1148.95) | 21.35(19.26-23.38) | 3728.73(3342.82-4074.89) | 30.30(27.45-33.10) | 2.17(1.78-2.56) |
| Italy | 7776.49(6450.60-9198.65) | 9.68(7.97-11.50) | 59745.48(53515.20-64591.29) | 43.50(39.95-46.84) | 5.97(4.88-7.08) |
| Jamaica | 193.03(175.34-212.97) | 10.37(9.40-11.45) | 778.80(602.02-980.75) | 25.29(19.47-31.85) | 3.43(3.07-3.79) |
| Japan | 118273.67(108494.49-128376.59) | 70.37(64.62-76.52) | 383903.14(335660.18-419247.70) | 97.21(87.13-105.88) | 0.82(0.63-1.02) |
| Jordan | 915.57(601.43-1478.26) | 57.49(38.26-93.38) | 4098.65(2971.74-5478.70) | 52.06(37.66-70.17) | -0.27(-0.44--0.09) |
| Kazakhstan | 3275.81(2909.19-3655.32) | 24.35(21.52-27.22) | 4398.35(3454.06-5532.22) | 23.60(18.54-29.66) | -0.3(-0.88-0.29) |
| Kenya | 2207.78(756.17-5543.25) | 22.84(7.43-61.20) | 7129.93(2396.91-20416.51) | 28.11(8.93-84.46) | 0.79(0.7-0.87) |
| Kiribati | 39.33(17.95-68.92) | 63.92(30.90-109.57) | 66.99(35.80-112.52) | 63.30(34.59-106.98) | -0.11(-0.16--0.06) |
| Kuwait | 437.31(396.82-474.46) | 60.48(53.86-66.52) | 1380.53(1161.50-1620.79) | 45.69(38.06-53.15) | 0.13(-0.78-1.04) |
| Kyrgyzstan | 941.10(818.28-1036.99) | 28.41(24.42-31.82) | 402.59(335.34-471.79) | 7.35(6.11-8.59) | -5.03(-5.98--4.07) |
| Lao People's Democratic Republic | 164.44(38.64-518.83) | 7.00(1.73-20.92) | 368.35(124.34-942.44) | 7.10(2.33-18.17) | 0.02(-0.01-0.04) |
| Latvia | 2977.03(2649.45-3316.03) | 84.00(74.70-93.18) | 329.09(279.60-383.97) | 9.37(7.86-11.12) | -7.79(-9.33--6.22) |
| Lebanon | 962.41(479.33-1754.36) | 43.48(21.50-79.91) | 2414.93(1824.32-3439.66) | 39.31(29.86-55.34) | 0.01(-0.14-0.15) |
| Lesotho | 305.08(92.36-587.28) | 35.40(10.43-69.12) | 418.36(128.97-829.35) | 37.04(11.27-73.77) | 0.41(0.23-0.59) |
| Liberia | 564.82(197.87-987.17) | 44.81(16.30-82.22) | 894.44(338.77-1810.77) | 35.06(12.82-73.94) | -0.67(-0.95--0.39) |
| Libya | 84.33(41.79-152.25) | 3.78(1.88-6.84) | 576.29(173.88-1653.80) | 9.74(2.78-28.88) | 3.83(3.54-4.11) |
| Lithuania | 1495.16(1288.97-1769.45) | 33.61(29.13-39.62) | 279.78(235.26-328.95) | 5.63(4.66-6.78) | -6.46(-8.04--4.86) |
| Luxembourg | 95.43(86.52-104.64) | 17.64(15.97-19.37) | 392.17(345.88-437.59) | 37.43(33.20-41.86) | 3.09(2.65-3.53) |
| Madagascar | 2429.90(824.47-4009.84) | 38.57(14.90-67.36) | 5845.94(2357.16-11427.64) | 40.76(16.49-80.39) | 0.08(0.03-0.12) |
| Malawi | 999.41(317.77-1700.46) | 21.20(7.59-37.98) | 2145.45(878.93-4025.46) | 24.08(9.77-47.05) | 0.29(0.2-0.38) |
| Malaysia | 2055.01(1574.58-2941.55) | 20.11(15.23-28.71) | 6816.17(5105.03-8876.05) | 23.33(17.30-30.58) | 0.57(0.44-0.7) |
| Maldives | 154.17(69.17-281.30) | 146.01(75.04-253.36) | 329.16(232.18-457.82) | 85.52(62.56-112.50) | -1.89(-1.97--1.81) |
| Mali | 2838.14(909.35-4779.61) | 65.42(22.08-109.52) | 5892.09(2196.91-11747.17) | 57.33(20.50-118.30) | -0.37(-0.46--0.28) |
| Malta | 167.88(152.87-181.81) | 39.56(36.15-42.86) | 860.80(758.80-963.36) | 88.88(79.16-99.48) | 2.85(2.42-3.29) |
| Marshall Islands | 21.00(11.81-34.94) | 66.11(37.97-111.50) | 30.93(17.05-55.89) | 61.64(33.85-112.18) | -0.22(-0.26--0.18) |
| Mauritania | 491.98(182.60-808.98) | 46.11(17.22-76.22) | 684.97(293.00-1254.91) | 29.51(12.32-54.90) | -1.46(-1.8--1.12) |
| Mauritius | 504.47(469.41-544.17) | 67.88(63.40-73.13) | 3505.12(3245.93-3724.90) | 200.52(184.87-212.76) | 3.86(3.36-4.37) |
| Mexico | 21336.94(20355.37-22354.96) | 45.77(43.60-47.95) | 102647.74(91207.78-115067.50) | 80.89(72.04-90.38) | 1.91(1.67-2.16) |
| Micronesia (Federated States of) | 59.55(30.74-96.82) | 73.58(40.40-121.25) | 53.94(32.84-93.29) | 58.78(35.48-101.82) | -0.72(-0.79--0.64) |
| Monaco | 34.13(25.01-45.12) | 53.31(39.98-68.91) | 51.80(37.08-70.43) | 58.19(42.40-78.91) | 0.35(0.22-0.48) |
| Mongolia | 884.60(549.15-1416.63) | 65.54(42.38-98.44) | 946.02(608.39-1368.04) | 37.34(24.02-54.79) | -2.28(-2.49--2.07) |
| Montenegro | 32.94(23.54-45.28) | 5.15(3.68-7.07) | 41.68(29.34-58.18) | 4.71(3.35-6.46) | -0.21(-0.26--0.17) |
| Morocco | 521.19(253.42-907.05) | 3.20(1.56-5.43) | 2705.10(1007.17-5894.75) | 7.65(2.76-17.01) | 3.47(3.21-3.73) |
| Mozambique | 1330.56(402.81-2436.18) | 17.72(6.39-31.73) | 2774.46(1097.87-5300.13) | 19.80(8.24-37.67) | 0.5(0.41-0.59) |
| Myanmar | 2580.21(613.19-8522.90) | 10.44(2.43-33.09) | 6074.24(2005.00-15214.07) | 12.50(4.01-31.63) | 0.57(0.51-0.62) |
| Namibia | 300.91(89.14-597.67) | 45.59(13.24-92.51) | 599.10(188.55-1214.89) | 42.24(13.16-85.49) | -0.37(-0.46--0.27) |
| Nauru | 5.92(3.21-10.59) | 73.74(41.22-131.88) | 6.13(3.13-11.50) | 67.92(35.28-125.92) | -0.3(-0.34--0.26) |
| Nepal | 10138.43(5266.81-16882.00) | 106.28(56.49-175.62) | 27663.65(18562.19-38518.63) | 119.01(79.90-165.70) | 0.51(0.35-0.67) |
| Netherlands | 2900.40(2620.81-3211.80) | 15.24(13.82-16.86) | 17595.16(15605.78-19384.56) | 50.88(45.67-55.63) | 4.13(3.52-4.75) |
| New Zealand | 1124.96(1033.89-1227.96) | 28.37(26.13-30.88) | 4826.74(4348.72-5265.77) | 56.32(51.15-60.99) | 2.25(2.04-2.45) |
| Nicaragua | 376.27(305.86-520.09) | 19.56(15.64-28.07) | 1320.13(875.42-1709.26) | 25.54(16.81-33.28) | 1.45(1.21-1.69) |
| Niger | 1476.45(479.33-2667.61) | 46.12(16.70-81.23) | 3029.88(1240.50-5699.27) | 32.31(12.63-63.88) | -0.96(-1.22--0.7) |
| Nigeria | 14950.28(5849.42-23657.76) | 32.72(12.89-52.29) | 26938.24(10934.70-48502.28) | 27.08(10.92-49.97) | -0.64(-0.69--0.58) |
| Niue | 1.42(1.01-2.12) | 63.96(45.06-96.03) | 1.41(0.93-2.16) | 85.01(56.19-133.47) | -0.3(-0.73-0.14) |
| North Macedonia | 143.06(109.68-187.90) | 7.37(5.66-9.70) | 236.42(140.91-387.37) | 7.58(4.51-12.24) | 0.17(0-0.34) |
| Northern Mariana Islands | 34.10(23.54-47.37) | 95.49(69.38-129.46) | 30.80(19.67-49.57) | 60.28(39.38-97.64) | -1.6(-1.92--1.27) |
| Norway | 1952.66(1803.57-2115.35) | 29.31(26.93-31.99) | 4672.41(4295.22-5012.23) | 47.57(44.10-51.06) | 1.92(1.63-2.2) |
| Oman | 125.27(84.26-197.64) | 14.84(9.78-24.16) | 474.81(290.54-718.31) | 20.14(11.97-30.90) | 1.67(1.35-2) |
| Pakistan | 32934.75(20838.16-47379.16) | 57.91(36.39-83.83) | 71586.34(48229.68-97471.88) | 58.58(39.37-80.83) | -0.11(-0.24-0.01) |
| Palau | 8.63(5.61-13.38) | 62.90(41.53-97.20) | 11.92(7.85-18.82) | 60.93(40.61-95.31) | -0.04(-0.11-0.02) |
| Palestine | 852.21(587.94-1172.72) | 90.53(63.16-122.90) | 2239.09(1579.75-2892.14) | 87.14(60.49-113.03) | 0(-0.09-0.1) |
| Panama | 477.45(438.71-516.77) | 28.89(26.53-31.40) | 2797.52(2218.73-3318.81) | 63.22(50.11-75.04) | 3.17(2.99-3.36) |
| Papua New Guinea | 2266.84(1372.01-3464.04) | 80.09(50.85-125.45) | 6598.42(4437.77-10357.44) | 86.94(55.92-141.03) | 0.28(0.21-0.36) |
| Paraguay | 501.41(391.80-749.91) | 20.55(15.82-30.89) | 1790.11(1147.54-2496.06) | 30.08(19.38-42.23) | 1.57(1.45-1.7) |
| Peru | 25130.92(18465.57-34915.11) | 198.95(147.16-272.02) | 82312.40(59628.99-106112.62) | 246.21(178.27-317.79) | 1.2(0.99-1.42) |
| Philippines | 728.94(558.18-933.72) | 2.16(1.65-2.86) | 1883.68(1392.74-2382.35) | 2.08(1.54-2.61) | -0.17(-0.24--0.1) |
| Poland | 11119.25(10325.20-12001.02) | 25.75(23.92-27.77) | 18701.56(17067.45-20554.04) | 28.95(26.45-31.86) | 0.69(0.32-1.07) |
| Portugal | 2612.95(2407.94-2822.23) | 19.66(18.15-21.20) | 12587.81(11192.88-13718.99) | 52.28(47.33-56.63) | 3.82(3.24-4.4) |
| Puerto Rico | 1348.61(1253.49-1440.78) | 37.56(34.98-40.17) | 4297.49(3603.44-5013.42) | 68.43(57.19-79.96) | 2.01(1.64-2.38) |
| Qatar | 35.60(27.03-49.64) | 26.96(20.17-41.27) | 258.95(162.18-378.47) | 21.84(13.48-32.24) | -0.45(-0.65--0.24) |
| Republic of Korea | 13334.11(9051.01-21096.56) | 45.83(30.25-74.13) | 46534.38(30378.30-59899.36) | 50.40(33.11-64.87) | 0.8(0.61-0.99) |
| Republic of Moldova | 630.37(554.14-720.72) | 14.64(12.92-16.63) | 162.47(119.20-212.32) | 3.06(2.22-4.05) | -5.64(-6.87--4.4) |
| Romania | 19156.59(16947.82-21598.14) | 71.34(63.51-79.40) | 10894.25(9533.94-12331.40) | 34.87(30.69-39.52) | -2.14(-2.46--1.82) |
| Russian Federation | 41660.80(35359.55-47064.34) | 23.73(20.17-26.80) | 25017.86(22852.70-27525.43) | 11.33(10.34-12.50) | -3.82(-4.74--2.89) |
| Rwanda | 1163.55(330.81-2121.99) | 32.31(10.51-55.85) | 1794.40(724.47-3868.88) | 24.34(9.47-53.63) | -1.43(-1.67--1.2) |
| Saint Kitts and Nevis | 8.45(7.11-9.91) | 22.86(19.31-26.81) | 24.77(20.82-29.35) | 37.96(32.36-44.45) | 2.18(1.84-2.52) |
| Saint Lucia | 27.58(24.58-31.25) | 30.18(26.83-34.08) | 127.90(105.11-152.88) | 56.17(46.54-67.01) | 2.27(2.08-2.47) |
| Saint Vincent and the Grenadines | 3.67(3.18-4.21) | 4.98(4.33-5.69) | 37.84(32.96-42.93) | 27.81(24.30-31.52) | 5.67(4.66-6.69) |
| Samoa | 86.60(54.51-139.65) | 66.63(42.29-108.66) | 103.89(64.22-177.24) | 58.85(36.49-100.23) | -0.32(-0.41--0.23) |
| San Marino | 4.65(3.40-6.22) | 13.31(9.83-17.55) | 6.70(4.21-10.79) | 9.34(5.99-14.92) | -0.31(-0.59--0.03) |
| Sao Tome and Principe | 65.19(23.01-115.22) | 95.88(34.10-172.70) | 107.69(41.19-239.98) | 87.61(34.05-190.10) | -0.36(-0.44--0.27) |
| Saudi Arabia | 6173.97(3928.65-10032.40) | 94.75(62.06-156.57) | 26637.55(18326.01-36864.35) | 117.12(78.33-162.70) | 1.04(0.85-1.23) |
| Senegal | 1645.71(566.08-2679.08) | 45.84(16.68-75.78) | 2917.09(1232.44-5464.56) | 34.25(14.07-65.65) | -0.74(-1.09--0.38) |
| Serbia | 1612.87(1229.25-2160.59) | 15.12(11.44-20.65) | 1929.75(1238.41-2673.18) | 12.93(8.36-17.78) | -0.33(-0.42--0.23) |
| Seychelles | 3.48(1.27-8.40) | 5.98(2.16-14.39) | 6.36(2.41-14.60) | 5.45(2.06-12.71) | -0.22(-0.29--0.15) |
| Sierra Leone | 921.50(312.62-1566.49) | 41.60(14.57-72.91) | 1378.12(527.57-2660.67) | 31.57(12.04-63.86) | -0.71(-0.98--0.44) |
| Singapore | 533.03(478.25-594.94) | 23.44(21.27-25.86) | 2058.23(1824.51-2325.52) | 24.67(21.84-27.75) | 0.56(0.37-0.75) |
| Slovakia | 1023.31(775.66-1442.26) | 17.44(13.24-24.48) | 1524.15(949.98-2233.29) | 17.35(10.89-25.46) | 0.3(0.18-0.42) |
| Slovenia | 648.66(595.15-707.58) | 27.36(25.12-29.89) | 1355.20(1153.76-1559.28) | 32.51(27.86-37.51) | 0.77(0.53-1.01) |
| Solomon Islands | 68.97(44.66-102.35) | 32.64(21.69-48.61) | 173.17(113.59-295.77) | 34.82(22.94-59.08) | 0.25(0.15-0.35) |
| Somalia | 913.60(239.25-1766.23) | 29.78(9.07-54.94) | 1963.01(702.76-3663.29) | 24.73(9.89-44.63) | -0.62(-0.68--0.55) |
| South Africa | 12120.63(7743.20-16434.87) | 54.31(33.06-76.23) | 23448.43(17434.40-32908.18) | 50.17(37.17-70.12) | -0.48(-0.73--0.23) |
| South Sudan | 887.63(281.73-1761.09) | 29.32(10.24-57.56) | 1200.90(466.54-2321.15) | 25.97(10.48-53.42) | -0.52(-0.74--0.3) |
| Spain | 22323.38(20742.18-23925.04) | 41.59(38.69-44.52) | 83333.79(71906.03-91469.16) | 80.43(71.28-87.44) | 2.48(2.19-2.77) |
| Sri Lanka | 3274.62(2535.80-4567.99) | 28.77(22.28-40.32) | 6422.40(3840.03-10480.20) | 24.41(14.57-39.23) | -0.51(-0.7--0.32) |
| Sudan | 382.99(161.27-840.03) | 3.41(1.46-6.97) | 1879.75(618.98-4733.62) | 7.82(2.46-19.96) | 3.37(3.08-3.65) |
| Suriname | 73.10(57.51-104.02) | 26.51(20.80-38.54) | 216.38(133.54-298.82) | 34.68(21.39-47.92) | 1.36(1.17-1.54) |
| Sweden | 4968.38(4552.50-5397.69) | 33.50(30.72-36.43) | 12085.73(10697.24-13557.42) | 54.67(48.53-60.89) | 2.02(1.86-2.18) |
| Switzerland | 2726.77(2463.78-3013.38) | 26.52(23.93-29.32) | 6633.24(5821.97-7304.68) | 36.92(32.88-40.46) | 1.82(1.5-2.14) |
| Syrian Arab Republic | 1028.37(729.40-1545.75) | 17.24(11.83-26.45) | 3023.42(2019.50-4612.53) | 23.47(15.83-35.97) | 1.14(1.03-1.26) |
| Taiwan (Province of China) | 1087.29(968.99-1220.33) | 6.83(6.11-7.64) | 8327.48(7462.02-9247.94) | 19.83(17.86-21.99) | 3.71(3.08-4.33) |
| Tajikistan | 2567.71(1489.36-4108.31) | 89.60(51.58-144.85) | 3854.49(2212.90-6769.55) | 65.05(37.99-114.85) | -1.32(-1.45--1.19) |
| Thailand | 2634.16(2014.31-3373.85) | 6.71(5.12-8.91) | 5828.62(4146.36-8847.49) | 5.79(4.12-8.71) | -0.77(-0.89--0.65) |
| Timor-Leste | 21.58(6.43-58.42) | 5.91(1.80-15.27) | 60.12(20.25-141.13) | 6.43(2.16-15.01) | 0.41(0.22-0.6) |
| Togo | 657.28(238.75-1093.18) | 45.14(17.44-78.81) | 1585.89(624.24-3082.55) | 36.25(13.88-73.85) | -0.61(-0.84--0.39) |
| Tokelau | 0.85(0.45-1.44) | 58.36(32.08-99.91) | 1.15(0.71-1.82) | 83.05(51.60-132.27) | -0.07(-0.54-0.39) |
| Tonga | 31.75(22.29-48.80) | 42.61(29.58-67.06) | 39.04(23.74-66.67) | 42.59(25.75-73.56) | 0.13(0.07-0.2) |
| Trinidad and Tobago | 318.13(293.44-345.30) | 36.30(33.37-39.40) | 1118.17(884.21-1411.65) | 61.05(48.38-76.79) | 2.33(2.06-2.61) |
| Tunisia | 190.29(96.14-315.38) | 3.39(1.70-5.71) | 962.59(384.19-2475.82) | 7.21(2.83-18.62) | 2.93(2.73-3.14) |
| Turkey | 9156.68(5633.97-14550.51) | 24.24(14.92-38.36) | 25362.78(17689.02-34576.41) | 27.31(19.22-37.30) | 0.77(0.56-0.98) |
| Turkmenistan | 784.08(670.78-884.55) | 34.66(29.73-38.39) | 870.11(696.17-1118.33) | 18.20(14.72-23.09) | -2.68(-3.3--2.05) |
| Tuvalu | 5.66(2.95-10.12) | 68.64(36.94-121.00) | 6.43(3.73-10.61) | 56.00(32.53-93.49) | -0.69(-0.74--0.64) |
| Uganda | 2139.70(749.08-4081.51) | 27.60(9.87-54.60) | 4673.97(1796.53-10621.25) | 25.52(9.76-58.17) | -0.53(-0.62--0.44) |
| Ukraine | 26162.60(23334.97-28937.86) | 38.64(34.60-42.81) | 6953.67(5328.52-8778.20) | 10.47(8.07-13.20) | -5.44(-5.87--5) |
| United Arab Emirates | 806.62(494.91-1297.29) | 123.77(76.77-198.24) | 4382.44(3079.86-6262.86) | 105.79(75.90-151.27) | 1.04(0.55-1.54) |
| United Kingdom | 40195.21(37691.70-42908.18) | 45.65(42.69-48.95) | 143237.88(132272.94-150345.13) | 108.25(101.07-113.45) | 3.87(3.43-4.31) |
| United Republic of Tanzania | 2646.09(956.50-4529.55) | 20.04(7.64-36.35) | 6019.49(2395.91-12684.70) | 19.59(7.76-41.93) | -0.15(-0.18--0.12) |
| United States of America | 194744.25(180633.93-210217.80) | 62.96(58.45-68.03) | 524808.48(478755.29-560666.59) | 91.67(84.23-97.91) | 1.22(0.93-1.5) |
| United States Virgin Islands | 34.89(27.05-50.19) | 40.42(31.09-57.87) | 52.90(36.90-76.14) | 35.18(23.43-52.12) | -0.1(-0.32-0.13) |
| Uruguay | 1220.44(1118.60-1327.46) | 31.88(29.30-34.68) | 3323.03(3051.60-3575.17) | 59.55(55.20-63.82) | 2.32(2.08-2.55) |
| Uzbekistan | 7070.84(5979.64-8332.33) | 56.93(47.00-69.24) | 4838.57(4017.49-5886.21) | 17.22(14.59-20.74) | -4.55(-5.24--3.85) |
| Vanuatu | 72.66(36.35-115.11) | 65.12(34.69-105.02) | 165.03(88.15-282.72) | 64.07(34.78-111.26) | -0.23(-0.32--0.13) |
| Venezuela (Bolivarian Republic of) | 2467.25(2263.57-2692.67) | 22.29(20.37-24.42) | 10579.64(8171.66-13348.33) | 36.21(28.12-45.32) | 1.96(1.71-2.21) |
| Viet Nam | 2371.09(790.90-5756.59) | 5.63(1.82-13.88) | 6768.48(2510.82-15187.36) | 6.89(2.50-15.68) | 0.8(0.74-0.87) |
| Yemen | 199.20(90.78-406.92) | 3.21(1.46-6.17) | 1177.64(422.66-2763.57) | 6.97(2.33-16.87) | 3.17(2.86-3.48) |
| Zambia | 824.04(281.35-1454.13) | 23.14(8.87-38.80) | 2286.98(942.87-4400.03) | 26.19(10.88-50.65) | 0.38(0.3-0.45) |
| Zimbabwe | 519.78(183.39-861.97) | 12.44(4.10-21.34) | 1018.17(343.66-1864.28) | 13.76(4.36-25.67) | 0.37(0.23-0.52) |

ILD&PS, Interstitial lung disease and pulmonary sarcoidosis; DALYs, Disability-adjusted life years; ASDR, Age-standardized DALYs rate; EAPC, Estimated annual percentage change; UI, Uncertainty interval; CI, Confidence interval.

**Table S9. Number and age-standardized mortality of ILD&PS, 1990 vs. 2021 (Nations).**

| **Location** | **Number in 1990  (95% UI)** | **ASMR in 1990  (per 100 000, 95% UI)** | **Number in 2021  (95% UI)** | **ASMR in 2021 (per 100 000, 95% UI)** | **EAPC of ASMR (95% CI)** |
| --- | --- | --- | --- | --- | --- |
| Afghanistan | 3.83(0.05-15.47) | 0.06(0.00-0.25) | 22.05(1.90-79.55) | 0.26(0.02-0.86) | 5.9(5.31-6.49) |
| Albania | 22.39(14.20-33.76) | 1.18(0.75-1.79) | 36.03(18.66-62.30) | 0.84(0.43-1.44) | -1.12(-1.31--0.94) |
| Algeria | 4.05(0.10-13.17) | 0.04(0.00-0.14) | 59.31(4.88-217.89) | 0.21(0.02-0.73) | 6.88(6.2-7.56) |
| American Samoa | 0.34(0.20-0.46) | 1.26(0.74-1.73) | 0.38(0.26-0.64) | 0.86(0.60-1.44) | -1.2(-1.42--0.98) |
| Andorra | 1.17(0.65-1.96) | 2.19(1.24-3.66) | 3.18(0.94-6.25) | 1.98(0.59-3.86) | -0.02(-0.22-0.18) |
| Angola | 39.72(13.13-79.37) | 1.22(0.43-2.65) | 103.01(38.11-191.97) | 1.06(0.40-2.10) | -0.63(-0.68--0.58) |
| Antigua and Barbuda | 0.27(0.24-0.30) | 0.46(0.41-0.51) | 1.05(0.97-1.15) | 1.13(1.03-1.23) | 3.52(3.18-3.86) |
| Argentina | 730.69(662.92-800.11) | 2.37(2.14-2.59) | 2000.97(1784.82-2186.36) | 3.44(3.08-3.76) | 1.57(1.13-2.02) |
| Armenia | 55.22(50.31-60.20) | 2.14(1.93-2.32) | 57.15(49.74-64.93) | 1.34(1.17-1.53) | 0.02(-1.08-1.13) |
| Australia | 214.79(195.39-233.38) | 1.11(1.01-1.21) | 1632.58(1371.04-1797.09) | 3.21(2.72-3.51) | 3.66(3.05-4.29) |
| Austria | 67.03(61.85-72.36) | 0.54(0.50-0.58) | 249.17(215.86-276.24) | 1.22(1.07-1.34) | 3.78(3.48-4.09) |
| Azerbaijan | 61.45(28.89-113.56) | 1.35(0.61-2.63) | 72.03(32.81-128.93) | 0.81(0.38-1.42) | -1.84(-2.24--1.45) |
| Bahamas | 1.85(1.67-2.04) | 1.24(1.11-1.37) | 10.30(8.35-12.56) | 2.78(2.28-3.36) | 3.12(2.85-3.4) |
| Bahrain | 2.99(2.06-5.27) | 2.43(1.71-4.34) | 17.39(10.12-24.94) | 3.16(1.91-4.53) | 1.06(0.7-1.43) |
| Bangladesh | 1374.10(748.06-2190.08) | 3.13(1.68-5.00) | 3504.82(2282.79-5282.83) | 2.72(1.77-4.08) | -0.58(-0.75--0.41) |
| Barbados | 2.76(2.49-3.02) | 0.90(0.81-0.98) | 9.73(7.88-11.80) | 1.90(1.55-2.30) | 3.27(2.92-3.63) |
| Belarus | 232.80(199.69-264.31) | 1.93(1.65-2.20) | 41.89(34.12-50.17) | 0.26(0.22-0.32) | -7.61(-8.18--7.03) |
| Belgium | 161.75(147.70-173.59) | 1.01(0.92-1.08) | 568.20(488.77-624.92) | 2.16(1.90-2.36) | 3.21(2.89-3.53) |
| Belize | 2.12(1.80-2.53) | 2.15(1.81-2.60) | 13.30(11.41-15.37) | 4.57(3.90-5.34) | 2.81(2.39-3.23) |
| Benin | 37.81(13.83-65.18) | 2.02(0.73-3.59) | 63.93(23.39-137.91) | 1.37(0.49-3.03) | -1.19(-1.45--0.94) |
| Bermuda | 1.63(1.38-1.90) | 2.80(2.37-3.26) | 5.38(4.52-6.44) | 3.69(3.10-4.43) | 1.22(1-1.43) |
| Bhutan | 5.96(3.43-8.93) | 2.98(1.78-4.45) | 18.35(11.53-28.81) | 3.29(2.05-5.16) | 0.25(0.2-0.3) |
| Bolivia (Plurinational State of) | 216.73(103.51-429.31) | 8.23(4.09-15.86) | 729.91(457.59-1068.74) | 9.74(6.25-14.08) | 0.82(0.72-0.92) |
| Bosnia and Herzegovina | 30.95(18.30-48.51) | 0.83(0.49-1.31) | 33.66(18.92-56.12) | 0.54(0.31-0.91) | -1.6(-1.78--1.42) |
| Botswana | 9.35(2.17-19.30) | 2.14(0.50-4.53) | 16.79(4.15-36.13) | 1.44(0.36-3.08) | -1.21(-1.35--1.07) |
| Brazil | 827.40(781.13-873.83) | 0.98(0.91-1.05) | 4452.50(4017.32-4762.87) | 1.84(1.66-1.97) | 2.14(1.8-2.47) |
| Brunei Darussalam | 2.82(1.83-4.09) | 3.21(2.07-4.61) | 6.77(4.57-9.29) | 2.50(1.62-3.46) | -0.58(-0.73--0.43) |
| Bulgaria | 48.31(42.26-55.25) | 0.43(0.38-0.48) | 67.08(55.69-79.95) | 0.48(0.40-0.57) | 0.42(0.12-0.71) |
| Burkina Faso | 46.78(15.22-79.34) | 1.25(0.42-2.19) | 79.56(30.28-150.07) | 0.95(0.36-1.85) | -0.94(-1.07--0.82) |
| Burundi | 27.46(8.14-48.57) | 1.24(0.40-2.26) | 44.15(14.79-94.28) | 1.03(0.34-2.26) | -0.83(-0.98--0.67) |
| Cabo Verde | 6.09(1.62-12.95) | 2.55(0.68-5.42) | 4.70(1.78-8.82) | 1.11(0.42-2.06) | -2.45(-3.14--1.75) |
| Cambodia | 6.04(0.91-20.73) | 0.16(0.02-0.52) | 18.73(3.64-57.94) | 0.19(0.04-0.57) | 0.65(0.57-0.73) |
| Cameroon | 90.84(34.47-154.77) | 2.37(0.90-4.18) | 177.69(66.12-331.64) | 1.57(0.59-2.97) | -1.27(-1.5--1.04) |
| Canada | 665.29(608.25-720.69) | 2.04(1.86-2.21) | 3133.21(2760.17-3408.52) | 3.92(3.47-4.27) | 2.55(2.28-2.81) |
| Central African Republic | 13.94(4.14-26.94) | 1.53(0.50-3.29) | 25.61(8.89-52.99) | 1.43(0.53-3.42) | -0.25(-0.31--0.18) |
| Chad | 48.40(16.49-86.81) | 1.85(0.65-3.27) | 79.99(31.52-149.97) | 1.54(0.61-3.01) | -0.47(-0.66--0.28) |
| Chile | 356.05(332.56-379.48) | 3.83(3.56-4.10) | 2169.38(1943.36-2335.89) | 8.25(7.41-8.88) | 2.68(2.31-3.05) |
| China | 2954.65(2186.15-4687.88) | 0.41(0.31-0.66) | 7674.10(4637.26-10371.39) | 0.39(0.24-0.53) | 0.24(0.06-0.42) |
| Colombia | 83.03(77.39-88.35) | 0.49(0.46-0.53) | 811.57(672.60-960.50) | 1.47(1.22-1.74) | 3.55(3.1-4.01) |
| Comoros | 2.02(0.62-3.93) | 1.15(0.36-2.30) | 3.99(1.45-8.62) | 0.90(0.32-1.97) | -0.94(-1.15--0.73) |
| Congo | 14.41(4.49-30.79) | 1.62(0.53-3.74) | 30.35(11.63-57.84) | 1.35(0.51-2.72) | -0.77(-0.85--0.69) |
| Cook Islands | 0.17(0.11-0.28) | 1.29(0.89-2.19) | 0.21(0.10-0.41) | 0.91(0.45-1.78) | -1.29(-1.4--1.18) |
| Costa Rica | 33.55(30.48-36.20) | 1.96(1.76-2.12) | 179.10(156.49-200.88) | 3.25(2.84-3.64) | 1.99(1.55-2.42) |
| Croatia | 70.53(24.52-122.16) | 2.17(0.78-3.84) | 141.63(49.26-276.76) | 1.45(0.52-2.94) | -1.2(-1.43--0.97) |
| Cuba | 15.80(14.73-17.09) | 0.28(0.26-0.30) | 30.89(27.02-34.83) | 0.33(0.29-0.38) | 1.16(0.89-1.44) |
| Cyprus | 17.04(15.62-18.52) | 0.17(0.16-0.19) | 65.32(56.81-73.86) | 0.33(0.29-0.38) | 2.67(2.36-2.98) |
| Czechia | 48.12(30.72-75.99) | 8.03(5.22-12.30) | 88.94(60.41-123.09) | 4.46(3.01-6.14) | -1.72(-2.01--1.43) |
| Côte d'Ivoire | 95.21(83.72-109.70) | 0.71(0.63-0.81) | 354.96(304.89-409.59) | 1.56(1.34-1.80) | 3.64(3.3-3.97) |
| Democratic People's Republic of Korea | 51.83(25.28-106.81) | 0.38(0.19-0.77) | 127.49(66.66-246.90) | 0.41(0.22-0.79) | 0.53(0.42-0.64) |
| Democratic Republic of the Congo | 181.08(61.04-410.34) | 1.44(0.47-3.68) | 491.69(169.62-1335.41) | 1.62(0.52-4.85) | 0.4(0.26-0.54) |
| Denmark | 88.58(81.03-96.75) | 1.05(0.96-1.13) | 316.97(275.26-357.20) | 2.40(2.10-2.67) | 2.97(2.8-3.14) |
| Djibouti | 1.09(0.35-1.98) | 0.95(0.33-1.75) | 3.99(1.35-8.50) | 0.75(0.26-1.55) | -0.86(-0.99--0.73) |
| Dominica | 0.45(0.34-0.70) | 0.77(0.57-1.18) | 0.90(0.57-1.28) | 1.15(0.72-1.63) | 1.45(1.31-1.6) |
| Dominican Republic | 11.56(6.85-23.03) | 0.34(0.20-0.71) | 47.39(24.83-83.58) | 0.48(0.25-0.84) | 1.48(1.24-1.72) |
| Ecuador | 150.21(133.89-167.54) | 3.27(2.91-3.66) | 1208.57(996.19-1470.91) | 8.11(6.71-9.78) | 4.09(3.57-4.6) |
| Egypt | 153.50(110.94-223.18) | 0.70(0.50-1.02) | 219.97(154.21-311.63) | 0.46(0.32-0.63) | -1.66(-1.95--1.37) |
| El Salvador | 44.00(32.50-68.61) | 1.48(1.08-2.35) | 116.67(70.85-159.61) | 1.79(1.09-2.44) | 1.02(0.85-1.2) |
| Equatorial Guinea | 2.33(0.73-4.50) | 1.42(0.49-2.88) | 5.16(1.75-11.11) | 1.16(0.40-2.50) | -0.75(-0.8--0.69) |
| Eritrea | 11.44(2.84-21.69) | 1.13(0.35-1.97) | 24.96(9.33-42.61) | 1.02(0.40-1.79) | -0.4(-0.49--0.31) |
| Estonia | 54.87(48.71-62.03) | 2.76(2.46-3.10) | 9.45(8.03-10.87) | 0.30(0.26-0.35) | -8.83(-10.9--6.71) |
| Eswatini | 5.04(1.25-10.39) | 2.13(0.52-4.51) | 8.40(2.39-16.61) | 1.74(0.50-3.36) | -0.43(-0.59--0.27) |
| Ethiopia | 164.36(42.65-292.16) | 0.90(0.26-1.62) | 306.98(96.33-653.59) | 0.76(0.23-1.65) | -0.82(-0.91--0.72) |
| Fiji | 2.81(2.00-3.78) | 0.65(0.46-0.89) | 5.05(2.96-7.23) | 0.69(0.41-0.98) | 0.29(0.2-0.39) |
| Finland | 94.42(83.74-106.24) | 1.28(1.14-1.44) | 445.29(380.54-493.55) | 3.00(2.60-3.30) | 3.46(3.17-3.75) |
| France | 875.68(790.33-946.01) | 0.99(0.90-1.07) | 3121.39(2622.12-3507.86) | 1.86(1.61-2.08) | 2.24(1.96-2.53) |
| Gabon | 7.47(2.57-14.17) | 1.47(0.51-2.90) | 10.15(3.62-22.22) | 1.15(0.42-2.53) | -0.89(-0.95--0.82) |
| Gambia | 6.54(2.23-11.78) | 2.12(0.73-3.91) | 15.10(5.82-31.00) | 1.67(0.65-3.50) | -0.72(-1.01--0.43) |
| Georgia | 35.49(29.05-43.35) | 0.61(0.50-0.74) | 35.18(29.85-41.17) | 0.57(0.49-0.67) | 1.83(0.75-2.91) |
| Germany | 1490.12(1312.83-1674.28) | 1.14(1.01-1.28) | 4380.03(3812.94-4827.86) | 1.97(1.75-2.14) | 2.37(2.19-2.56) |
| Ghana | 77.10(26.96-125.95) | 1.44(0.51-2.38) | 213.22(87.48-357.50) | 1.47(0.60-2.42) | 0.23(0.13-0.32) |
| Greece | 61.47(56.08-66.70) | 0.42(0.38-0.45) | 545.85(479.42-598.28) | 1.95(1.76-2.12) | 5.98(5.23-6.74) |
| Greenland | 1.25(0.71-1.69) | 4.72(2.50-6.70) | 2.06(0.88-3.10) | 3.57(1.46-5.39) | -0.58(-0.77--0.39) |
| Grenada | 0.53(0.45-0.60) | 0.68(0.58-0.78) | 1.71(1.47-1.94) | 1.68(1.45-1.91) | 3.5(3.1-3.9) |
| Guam | 3.59(2.62-4.49) | 4.68(3.36-6.02) | 4.09(3.12-6.09) | 2.09(1.61-3.06) | -1.97(-2.2--1.74) |
| Guatemala | 74.15(62.81-88.96) | 2.44(2.10-2.84) | 337.37(288.72-392.31) | 3.25(2.80-3.77) | 1.08(0.65-1.51) |
| Guinea | 57.88(21.59-99.14) | 1.93(0.73-3.38) | 78.83(30.52-162.52) | 1.53(0.58-3.29) | -0.58(-0.78--0.37) |
| Guinea-Bissau | 8.71(2.60-16.56) | 2.48(0.83-4.38) | 10.98(4.34-19.14) | 1.70(0.72-3.03) | -1.05(-1.29--0.8) |
| Guyana | 5.17(4.54-5.81) | 1.46(1.28-1.64) | 10.45(7.90-13.44) | 1.75(1.34-2.22) | 1.82(1.02-2.63) |
| Haiti | 45.69(16.08-81.13) | 1.60(0.62-2.71) | 129.15(56.50-240.84) | 2.02(0.93-3.82) | 0.94(0.83-1.06) |
| Honduras | 45.72(30.00-71.08) | 2.22(1.46-3.44) | 200.78(101.35-297.60) | 3.41(1.69-5.05) | 1.62(1.46-1.79) |
| Hungary | 134.78(125.56-144.93) | 0.95(0.88-1.01) | 246.22(214.03-277.61) | 1.25(1.09-1.42) | 1.62(1.27-1.97) |
| Iceland | 1.98(1.76-2.16) | 0.66(0.59-0.72) | 12.41(10.64-14.06) | 1.97(1.70-2.21) | 4.24(3.83-4.65) |
| India | 14472.03(7858.65-23087.63) | 3.81(2.10-6.01) | 47336.08(30672.40-65803.94) | 4.48(2.95-6.28) | 0.76(0.6-0.92) |
| Indonesia | 195.46(34.34-594.04) | 0.22(0.04-0.68) | 535.21(112.66-1496.70) | 0.26(0.05-0.75) | 0.58(0.51-0.64) |
| Iran (Islamic Republic of) | 3.96(1.27-7.63) | 0.02(0.01-0.04) | 31.85(4.59-58.61) | 0.05(0.01-0.09) | 3.83(3.3-4.37) |
| Iraq | 49.99(33.32-75.54) | 0.64(0.41-1.00) | 159.22(98.60-229.80) | 0.78(0.50-1.16) | 0.51(0.36-0.66) |
| Ireland | 85.22(79.16-92.41) | 2.04(1.90-2.21) | 397.99(336.97-453.68) | 4.72(4.02-5.37) | 3.59(3.19-4) |
| Israel | 48.07(43.61-52.82) | 0.99(0.90-1.08) | 190.62(163.48-210.22) | 1.43(1.24-1.58) | 2.43(1.94-2.92) |
| Italy | 190.78(178.08-199.37) | 0.21(0.20-0.22) | 3277.36(2813.27-3568.00) | 1.94(1.71-2.09) | 8.56(7.1-10.04) |
| Jamaica | 7.08(6.39-7.80) | 0.38(0.35-0.42) | 32.66(25.11-41.20) | 1.03(0.79-1.30) | 3.8(3.41-4.19) |
| Japan | 4703.14(4354.01-4901.32) | 2.84(2.61-2.97) | 24025.65(19890.74-26344.87) | 4.89(4.20-5.28) | 1.51(1.27-1.75) |
| Jordan | 27.75(17.57-47.69) | 2.40(1.52-4.07) | 133.66(90.50-186.38) | 2.24(1.51-3.14) | -0.13(-0.3-0.04) |
| Kazakhstan | 106.40(93.37-120.75) | 0.89(0.77-1.02) | 142.55(107.64-183.86) | 0.85(0.64-1.12) | -0.15(-0.82-0.52) |
| Kenya | 72.43(18.82-206.44) | 0.97(0.24-2.94) | 240.28(62.44-758.15) | 1.22(0.31-4.13) | 0.84(0.77-0.9) |
| Kiribati | 0.79(0.36-1.39) | 1.73(0.85-3.04) | 1.40(0.72-2.48) | 1.74(0.91-3.10) | -0.04(-0.07--0.01) |
| Kuwait | 13.71(12.31-15.04) | 2.72(2.39-3.02) | 51.53(41.95-60.40) | 2.22(1.79-2.61) | 0.53(-0.51-1.57) |
| Kyrgyzstan | 30.72(25.84-35.70) | 1.08(0.90-1.29) | 10.12(8.28-12.37) | 0.23(0.19-0.28) | -5.74(-6.86--4.6) |
| Lao People's Democratic Republic | 4.48(0.48-15.34) | 0.25(0.03-0.80) | 9.71(1.62-29.05) | 0.24(0.04-0.74) | -0.18(-0.2--0.15) |
| Latvia | 135.85(122.25-151.65) | 3.84(3.45-4.25) | 13.57(11.59-15.72) | 0.31(0.26-0.36) | -9.22(-11.2--7.2) |
| Lebanon | 35.99(16.87-67.97) | 1.89(0.90-3.52) | 121.64(87.26-181.42) | 1.88(1.35-2.79) | 0.38(0.21-0.55) |
| Lesotho | 12.01(2.75-24.10) | 1.60(0.36-3.28) | 14.69(3.52-30.83) | 1.55(0.38-3.13) | 0.2(0.04-0.36) |
| Liberia | 20.97(7.08-38.75) | 2.03(0.70-3.87) | 28.87(9.77-62.38) | 1.53(0.51-3.50) | -0.8(-1.07--0.53) |
| Libya | 0.83(0.02-2.86) | 0.05(0.00-0.16) | 14.40(1.07-56.05) | 0.30(0.02-1.20) | 8.21(7.54-8.88) |
| Lithuania | 66.01(57.19-77.24) | 1.48(1.29-1.72) | 10.02(8.71-11.31) | 0.16(0.14-0.18) | -8.59(-11.03--6.09) |
| Luxembourg | 4.12(3.79-4.47) | 0.74(0.68-0.80) | 20.69(18.23-23.24) | 1.81(1.60-2.03) | 3.72(3.28-4.16) |
| Madagascar | 75.45(26.99-133.67) | 1.62(0.59-3.06) | 164.86(62.01-330.78) | 1.71(0.63-3.54) | 0.08(0.03-0.13) |
| Malawi | 29.98(9.44-55.39) | 0.87(0.29-1.66) | 66.93(24.74-134.29) | 0.99(0.36-2.03) | 0.3(0.22-0.38) |
| Malaysia | 69.67(51.99-102.74) | 0.79(0.58-1.18) | 240.09(168.23-323.46) | 0.93(0.65-1.25) | 0.62(0.41-0.83) |
| Maldives | 4.61(2.22-8.22) | 6.09(3.17-10.63) | 10.70(7.48-14.52) | 3.48(2.41-4.77) | -1.96(-2.05--1.87) |
| Mali | 97.87(31.47-167.28) | 2.81(0.94-4.93) | 195.88(67.56-407.61) | 2.44(0.83-5.17) | -0.39(-0.49--0.29) |
| Malta | 7.62(6.86-8.34) | 1.85(1.67-2.03) | 47.65(40.62-54.09) | 4.38(3.76-4.96) | 3.1(2.64-3.56) |
| Marshall Islands | 0.42(0.22-0.72) | 1.82(1.00-3.22) | 0.65(0.32-1.26) | 1.69(0.85-3.27) | -0.23(-0.27--0.2) |
| Mauritania | 18.86(6.64-32.11) | 2.09(0.75-3.51) | 25.76(9.89-49.46) | 1.33(0.50-2.58) | -1.47(-1.83--1.12) |
| Mauritius | 18.81(17.43-20.23) | 2.98(2.74-3.22) | 162.06(146.97-173.31) | 9.57(8.60-10.25) | 4.25(3.73-4.77) |
| Mexico | 731.36(706.49-751.30) | 1.91(1.83-1.97) | 4232.35(3748.98-4703.62) | 3.56(3.15-3.96) | 2.16(1.88-2.44) |
| Micronesia (Federated States of) | 1.24(0.66-2.08) | 2.01(1.12-3.41) | 1.23(0.70-2.24) | 1.67(0.93-3.00) | -0.6(-0.68--0.51) |
| Monaco | 1.81(1.24-2.48) | 2.34(1.62-3.14) | 2.91(1.98-4.18) | 2.64(1.83-3.81) | 0.51(0.38-0.65) |
| Mongolia | 26.26(16.09-39.93) | 2.54(1.52-3.89) | 29.79(17.91-45.76) | 1.51(0.91-2.46) | -2.19(-2.44--1.95) |
| Montenegro | 0.67(0.39-1.15) | 0.11(0.06-0.19) | 1.03(0.56-1.69) | 0.11(0.06-0.18) | 0(-0.2-0.2) |
| Morocco | 5.09(0.10-16.74) | 0.04(0.00-0.13) | 63.89(6.18-199.84) | 0.21(0.02-0.67) | 7.17(6.5-7.83) |
| Mozambique | 38.41(11.86-72.01) | 0.71(0.24-1.40) | 80.87(29.45-158.75) | 0.79(0.31-1.63) | 0.47(0.36-0.57) |
| Myanmar | 77.79(10.33-276.48) | 0.40(0.05-1.38) | 197.41(37.33-597.24) | 0.48(0.09-1.47) | 0.51(0.46-0.55) |
| Namibia | 10.72(2.56-22.39) | 2.07(0.49-4.51) | 21.49(5.32-44.97) | 1.86(0.47-3.84) | -0.45(-0.54--0.36) |
| Nauru | 0.12(0.06-0.22) | 1.98(1.07-3.67) | 0.13(0.06-0.25) | 1.91(0.96-3.77) | -0.13(-0.18--0.08) |
| Nepal | 372.18(189.44-631.04) | 4.76(2.47-7.78) | 1118.34(722.06-1594.27) | 5.41(3.49-7.70) | 0.57(0.41-0.74) |
| Netherlands | 111.15(101.66-119.71) | 0.55(0.51-0.59) | 981.07(836.10-1091.31) | 2.54(2.18-2.81) | 5.12(4.41-5.83) |
| New Zealand | 48.64(44.46-52.72) | 1.22(1.12-1.32) | 271.20(235.74-298.57) | 2.97(2.60-3.26) | 2.92(2.68-3.17) |
| Nicaragua | 11.26(8.61-16.91) | 0.74(0.56-1.14) | 45.27(27.08-61.86) | 0.98(0.58-1.33) | 1.5(1.22-1.79) |
| Niger | 48.12(16.03-85.33) | 2.03(0.72-3.58) | 102.40(38.00-207.37) | 1.47(0.53-3.10) | -0.87(-1.12--0.62) |
| Nigeria | 576.77(210.32-941.59) | 1.49(0.55-2.44) | 960.15(350.11-1807.06) | 1.24(0.45-2.31) | -0.61(-0.66--0.56) |
| Niue | 0.04(0.03-0.06) | 1.77(1.20-2.86) | 0.03(0.02-0.06) | 1.87(1.17-3.06) | -0.48(-0.72--0.24) |
| North Macedonia | 3.63(2.58-5.07) | 0.21(0.15-0.29) | 7.30(3.43-13.65) | 0.24(0.12-0.45) | 0.52(0.27-0.78) |
| Northern Mariana Islands | 0.70(0.48-0.98) | 2.99(2.12-4.09) | 0.83(0.49-1.47) | 1.89(1.16-3.37) | -1.59(-1.98--1.21) |
| Norway | 86.36(80.60-90.76) | 1.16(1.09-1.22) | 245.16(218.20-262.47) | 2.23(2.01-2.38) | 2.38(2.12-2.64) |
| Oman | 3.43(2.08-5.97) | 0.55(0.33-0.97) | 12.78(6.51-21.28) | 0.80(0.42-1.26) | 2.09(1.63-2.55) |
| Pakistan | 1297.83(782.40-1911.59) | 2.60(1.56-3.84) | 2746.51(1804.69-3841.42) | 2.77(1.83-3.93) | 0.07(-0.08-0.22) |
| Palau | 0.19(0.12-0.30) | 1.69(1.09-2.80) | 0.29(0.18-0.50) | 1.60(1.03-2.73) | -0.09(-0.17--0.01) |
| Palestine | 31.92(21.60-44.43) | 4.21(2.87-5.79) | 79.78(50.51-105.94) | 4.08(2.52-5.46) | 0.03(-0.09-0.15) |
| Panama | 16.22(14.78-17.68) | 1.10(1.00-1.20) | 127.81(99.13-154.20) | 2.84(2.21-3.44) | 3.78(3.58-3.99) |
| Papua New Guinea | 50.60(31.04-81.11) | 2.57(1.56-4.32) | 151.49(92.43-253.44) | 2.80(1.59-4.84) | 0.31(0.24-0.38) |
| Paraguay | 19.19(14.19-29.71) | 0.90(0.67-1.40) | 74.59(46.09-108.18) | 1.36(0.83-1.97) | 1.76(1.63-1.88) |
| Peru | 1076.69(773.48-1518.68) | 9.75(6.89-13.64) | 4427.75(3073.09-5828.63) | 13.31(9.20-17.55) | 1.65(1.43-1.87) |
| Philippines | 11.45(7.43-17.28) | 0.05(0.03-0.08) | 35.69(20.97-48.48) | 0.05(0.03-0.07) | -0.01(-0.18-0.16) |
| Poland | 352.29(337.82-363.82) | 0.81(0.78-0.84) | 748.56(682.67-814.00) | 1.04(0.95-1.13) | 1.12(0.58-1.67) |
| Portugal | 106.94(98.68-115.12) | 0.81(0.75-0.88) | 723.71(614.58-799.51) | 2.50(2.17-2.74) | 4.48(3.83-5.14) |
| Puerto Rico | 55.16(50.67-59.66) | 1.59(1.46-1.71) | 226.68(188.21-266.80) | 2.86(2.35-3.35) | 1.93(1.57-2.3) |
| Qatar | 0.90(0.65-1.40) | 1.29(0.91-2.12) | 5.90(3.07-9.38) | 0.98(0.61-1.46) | -0.79(-1.11--0.47) |
| Republic of Korea | 472.58(284.64-837.54) | 2.00(1.18-3.57) | 2052.17(1110.08-2822.52) | 2.18(1.18-2.99) | 0.78(0.6-0.97) |
| Republic of Moldova | 22.27(19.60-25.45) | 0.59(0.53-0.67) | 2.37(2.04-2.71) | 0.04(0.03-0.05) | -10.08(-12.11--8) |
| Romania | 675.17(596.09-772.36) | 2.58(2.29-2.91) | 424.15(370.54-480.85) | 1.16(1.01-1.32) | -2.45(-2.84--2.06) |
| Russian Federation | 1489.49(1304.76-1639.60) | 0.90(0.79-0.99) | 857.71(794.11-925.11) | 0.36(0.34-0.39) | -4.76(-6--3.5) |
| Rwanda | 33.85(9.81-60.06) | 1.29(0.43-2.23) | 55.82(18.89-128.14) | 0.99(0.33-2.33) | -1.33(-1.55--1.12) |
| Saint Kitts and Nevis | 0.35(0.29-0.41) | 0.95(0.80-1.10) | 0.96(0.80-1.12) | 1.72(1.45-1.98) | 2.59(2.23-2.95) |
| Saint Lucia | 1.06(0.94-1.20) | 1.32(1.17-1.49) | 5.20(4.26-6.22) | 2.26(1.86-2.70) | 1.77(1.56-1.97) |
| Saint Vincent and the Grenadines | 0.13(0.11-0.14) | 0.19(0.17-0.21) | 1.56(1.36-1.76) | 1.19(1.04-1.34) | 6.12(5-7.26) |
| Samoa | 1.88(1.16-3.18) | 1.90(1.13-3.27) | 2.54(1.48-4.62) | 1.71(0.99-3.13) | -0.26(-0.33--0.19) |
| San Marino | 0.22(0.15-0.32) | 0.60(0.39-0.84) | 0.34(0.18-0.59) | 0.38(0.21-0.67) | -0.39(-0.75--0.02) |
| Sao Tome and Principe | 2.61(0.88-4.81) | 4.32(1.47-7.96) | 3.83(1.41-8.35) | 3.83(1.44-8.03) | -0.36(-0.42--0.3) |
| Saudi Arabia | 217.42(137.36-369.47) | 4.44(2.82-7.81) | 770.05(510.67-1084.26) | 5.31(3.27-7.60) | 0.92(0.71-1.12) |
| Senegal | 59.33(20.24-100.35) | 2.05(0.72-3.51) | 105.33(40.21-210.16) | 1.51(0.56-3.04) | -0.8(-1.17--0.43) |
| Serbia | 53.24(37.37-75.95) | 0.55(0.39-0.80) | 74.75(43.45-108.72) | 0.45(0.26-0.65) | -0.61(-0.71--0.51) |
| Seychelles | 0.11(0.02-0.34) | 0.20(0.04-0.60) | 0.18(0.03-0.51) | 0.17(0.03-0.51) | -0.43(-0.54--0.33) |
| Sierra Leone | 35.40(11.68-62.85) | 1.89(0.64-3.45) | 47.19(16.79-98.74) | 1.38(0.49-2.96) | -0.87(-1.13--0.62) |
| Singapore | 17.31(16.08-18.65) | 0.90(0.83-0.97) | 81.86(71.11-89.34) | 1.00(0.87-1.09) | 0.76(0.52-1) |
| Slovakia | 33.83(24.44-50.10) | 0.57(0.41-0.85) | 54.75(29.39-85.70) | 0.58(0.31-0.90) | 0.37(0.24-0.49) |
| Slovenia | 22.70(20.59-25.21) | 0.93(0.84-1.03) | 67.99(56.77-78.12) | 1.41(1.18-1.62) | 1.58(1.3-1.87) |
| Solomon Islands | 1.49(0.93-2.34) | 1.02(0.65-1.65) | 3.97(2.49-7.07) | 1.08(0.68-1.91) | 0.26(0.16-0.35) |
| Somalia | 25.44(6.54-48.19) | 1.24(0.37-2.29) | 55.11(19.26-102.36) | 0.98(0.37-1.85) | -0.71(-0.78--0.64) |
| South Africa | 439.35(247.04-654.46) | 2.35(1.29-3.59) | 893.51(649.08-1265.79) | 2.19(1.61-3.10) | -0.49(-0.76--0.22) |
| South Sudan | 29.53(9.31-60.54) | 1.23(0.40-2.55) | 36.83(13.59-77.91) | 1.08(0.41-2.38) | -0.53(-0.72--0.35) |
| Spain | 998.07(918.12-1068.77) | 1.81(1.67-1.94) | 5404.77(4469.69-6028.77) | 4.37(3.69-4.81) | 3.27(2.98-3.56) |
| Sri Lanka | 117.69(89.76-169.42) | 1.26(0.95-1.83) | 269.99(151.97-439.37) | 1.10(0.62-1.75) | -0.26(-0.47--0.05) |
| Sudan | 4.58(0.05-18.46) | 0.05(0.00-0.20) | 40.34(3.15-138.80) | 0.23(0.02-0.80) | 6.03(5.47-6.6) |
| Suriname | 2.56(1.92-3.90) | 1.06(0.78-1.64) | 7.65(4.40-11.07) | 1.27(0.72-1.83) | 1.15(0.96-1.34) |
| Sweden | 236.95(215.01-255.10) | 1.44(1.31-1.55) | 705.11(615.46-801.84) | 2.76(2.42-3.13) | 2.63(2.42-2.84) |
| Switzerland | 126.87(113.54-141.29) | 1.14(1.02-1.26) | 370.68(314.35-416.95) | 1.77(1.54-1.98) | 2.31(1.97-2.64) |
| Syrian Arab Republic | 32.83(21.44-54.06) | 0.71(0.44-1.14) | 103.78(62.76-169.57) | 1.02(0.62-1.64) | 1.28(1.13-1.43) |
| Taiwan (Province of China) | 35.58(32.72-38.39) | 0.26(0.24-0.28) | 388.08(340.93-433.35) | 0.89(0.78-0.99) | 4.23(3.51-4.96) |
| Tajikistan | 106.95(53.18-189.46) | 4.33(2.06-7.91) | 131.10(70.13-240.30) | 2.93(1.59-5.22) | -1.55(-1.75--1.35) |
| Thailand | 75.76(54.28-108.92) | 0.23(0.16-0.33) | 189.38(118.03-318.67) | 0.18(0.11-0.30) | -1.14(-1.27--1) |
| Timor-Leste | 0.50(0.08-1.52) | 0.20(0.03-0.59) | 1.66(0.29-4.63) | 0.21(0.04-0.60) | 0.3(0.11-0.49) |
| Togo | 21.66(7.77-38.09) | 2.00(0.73-3.62) | 51.42(18.59-106.97) | 1.56(0.57-3.38) | -0.73(-0.95--0.51) |
| Tokelau | 0.02(0.01-0.04) | 1.66(0.86-2.85) | 0.03(0.02-0.04) | 1.87(1.13-3.09) | -0.22(-0.46-0.02) |
| Tonga | 0.73(0.49-1.21) | 1.22(0.80-2.05) | 1.02(0.59-1.87) | 1.22(0.70-2.25) | 0.16(0.09-0.23) |
| Trinidad and Tobago | 12.16(11.08-13.34) | 1.59(1.44-1.75) | 45.98(35.75-58.40) | 2.48(1.93-3.13) | 2.19(1.86-2.52) |
| Tunisia | 1.59(0.04-5.17) | 0.04(0.00-0.12) | 22.23(1.69-85.30) | 0.18(0.01-0.70) | 6.55(5.96-7.13) |
| Turkey | 300.18(173.10-501.00) | 0.96(0.55-1.62) | 975.83(644.74-1429.58) | 1.12(0.74-1.66) | 0.92(0.64-1.2) |
| Turkmenistan | 23.38(19.68-25.96) | 1.27(1.06-1.41) | 21.38(16.52-27.90) | 0.52(0.40-0.66) | -3.58(-4.3--2.85) |
| Tuvalu | 0.13(0.07-0.24) | 1.90(1.02-3.43) | 0.16(0.09-0.29) | 1.57(0.86-2.80) | -0.6(-0.65--0.55) |
| Uganda | 69.02(22.02-139.74) | 1.17(0.37-2.47) | 142.99(46.99-341.32) | 1.05(0.34-2.60) | -0.61(-0.7--0.52) |
| Ukraine | 858.93(769.42-940.68) | 1.24(1.11-1.35) | 218.55(162.80-282.84) | 0.30(0.22-0.38) | -5.93(-6.39--5.46) |
| United Arab Emirates | 21.98(13.46-36.08) | 5.33(3.21-8.75) | 117.55(79.13-170.11) | 5.29(3.70-7.50) | 2.05(1.39-2.72) |
| United Kingdom | 1744.64(1656.81-1797.59) | 1.83(1.74-1.89) | 8411.95(7513.86-8862.23) | 5.67(5.12-5.95) | 4.8(4.32-5.28) |
| United Republic of Tanzania | 80.96(27.22-148.97) | 0.81(0.27-1.60) | 187.08(62.58-419.60) | 0.78(0.26-1.75) | -0.23(-0.27--0.18) |
| United States of America | 7398.93(6816.46-7738.07) | 2.25(2.09-2.36) | 26601.58(23244.93-28216.49) | 4.28(3.77-4.53) | 2.13(1.76-2.49) |
| United States Virgin Islands | 1.26(0.94-1.85) | 1.75(1.29-2.63) | 2.28(1.50-3.44) | 1.35(0.88-2.05) | -0.59(-0.86--0.31) |
| Uruguay | 53.38(48.35-58.30) | 1.36(1.24-1.49) | 185.93(164.31-203.64) | 2.91(2.61-3.16) | 2.8(2.54-3.07) |
| Uzbekistan | 269.11(208.17-357.37) | 2.44(1.84-3.34) | 130.51(109.51-157.01) | 0.58(0.49-0.69) | -5.15(-5.94--4.36) |
| Vanuatu | 1.45(0.72-2.41) | 1.81(0.94-2.98) | 3.55(1.80-6.43) | 1.78(0.91-3.23) | -0.22(-0.3--0.14) |
| Venezuela (Bolivarian Republic of) | 80.64(73.84-88.12) | 0.87(0.79-0.95) | 424.50(327.21-552.13) | 1.51(1.17-1.95) | 2.22(1.94-2.49) |
| Viet Nam | 75.39(13.43-221.25) | 0.21(0.04-0.60) | 203.15(36.88-564.77) | 0.24(0.04-0.68) | 0.64(0.56-0.72) |
| Yemen | 2.05(0.03-7.65) | 0.05(0.00-0.16) | 25.41(2.43-81.36) | 0.21(0.02-0.67) | 6.32(5.7-6.94) |
| Zambia | 24.59(8.20-43.04) | 0.94(0.33-1.70) | 67.75(25.39-136.19) | 1.05(0.38-2.13) | 0.37(0.28-0.45) |
| Zimbabwe | 16.87(3.12-32.23) | 0.53(0.10-1.00) | 31.18(6.30-63.41) | 0.56(0.11-1.14) | 0.29(0.15-0.42) |

ILD&PS, Interstitial lung disease and pulmonary sarcoidosis; ASMR, Age-standardized mortality rate; EAPC, Estimated annual percentage change; UI, Uncertainty interval; CI, Confidence interval.

**Table S10. Number and age-standardized prevalence of ILD&PS, 1990 vs. 2021 (Global distribution by sex and age).**

| **Items** | **Number in 1990  (95% UI)** | **ASPR in 1990  (per 100 000, 95% UI)** | **Number in 2021  (95% UI)** | **ASPR in 2021  (per 100 000, 95% UI)** | **EAPC of ASPR (95% CI)** |
| --- | --- | --- | --- | --- | --- |
| **Sex** |  |  |  |  |  |
| Male | 921706.73(784632.58-1078238.04) | 48.42(41.44-56.47) | 2149200.96(1902460.13-2433401.43) | 53.73(47.69-60.59) | 0.44(0.35-0.52) |
| Female | 965738.54(828761.65-1129611.72) | 44.42(38.17-51.94) | 2157426.76(1902023.19-2464269.61) | 47.30(41.74-54.01) | 0.29(0.22-0.36) |
| **Age-group** |  |  |  |  |  |
| 0-14 years | 0.00(0.00-0.00) | 0.00(0.00-0.00) | 0.00(0.00-0.00) | 0.00(0.00-0.00) | 0.00(0.00-0.00) |
| 15-19 years | 2492.03(880.39-5082.65) | 0.48(0.17-0.98) | 2933.05(1131.37-5788.96) | 0.47(0.18-0.93) | 0.13(0.04-0.22) |
| 20-24 years | 16146.26(8716.61-27483.30) | 3.28(1.77-5.59) | 18647.84(10253.79-31230.61) | 3.12(1.72-5.23) | -0.11(-0.16--0.05) |
| 25-29 years | 38971.51(24250.29-57085.77) | 8.80(5.48-12.90) | 44874.83(28588.92-64228.86) | 7.63(4.86-10.92) | -0.45(-0.5--0.41) |
| 30-34 years | 63454.82(40561.28-88388.09) | 16.46(10.52-22.93) | 79896.95(53148.26-110238.18) | 13.22(8.79-18.24) | -0.65(-0.71--0.6) |
| 35-39 years | 90753.46(60925.35-123705.13) | 25.76(17.30-35.12) | 118864.73(83580.57-158019.42) | 21.19(14.90-28.17) | -0.65(-0.69--0.61) |
| 40-44 years | 112979.67(80821.35-150143.29) | 39.44(28.21-52.41) | 163252.58(120037.56-213101.83) | 32.63(24.00-42.60) | -0.66(-0.7--0.62) |
| 45-49 years | 133095.50(96055.69-173859.20) | 57.32(41.37-74.88) | 232572.63(169920.59-298882.36) | 49.12(35.89-63.12) | -0.56(-0.61--0.51) |
| 50-54 years | 174801.20(128073.73-233630.07) | 82.23(60.25-109.91) | 330906.01(248122.46-429735.88) | 74.37(55.77-96.59) | -0.37(-0.44--0.29) |
| 55-59 years | 213865.47(164226.87-265974.58) | 115.48(88.68-143.61) | 431199.73(342749.33-535796.15) | 108.96(86.61-135.40) | -0.11(-0.19--0.02) |
| 60-64 years | 255096.82(203241.81-315409.72) | 158.83(126.54-196.38) | 518793.21(420560.63-632628.94) | 162.10(131.41-197.67) | 0.16(0.04-0.27) |
| 65-69 years | 254861.91(204501.97-311802.95) | 206.18(165.44-252.25) | 611071.14(497398.13-741096.41) | 221.53(180.32-268.67) | 0.46(0.34-0.59) |
| 70-74 years | 211934.42(173117.52-259654.67) | 250.33(204.48-306.70) | 632825.47(520341.87-762125.37) | 307.44(252.79-370.25) | 0.82(0.75-0.88) |
| 75-79 years | 170044.30(140332.00-203574.79) | 276.25(227.98-330.72) | 495581.27(409184.97-590168.65) | 375.77(310.26-447.49) | 1.03(0.94-1.13) |
| 80-84 years | 98239.44(82362.85-117153.13) | 277.70(232.82-331.17) | 353010.22(298074.81-414873.13) | 403.06(340.33-473.69) | 1.3(1.12-1.48) |
| 85-89 years | 38393.43(31865.36-45638.95) | 254.07(210.87-302.02) | 185407.61(156928.84-218788.28) | 405.51(343.23-478.52) | 1.7(1.46-1.94) |
| 90-94 years | 10087.45(8295.55-12082.34) | 235.40(193.59-281.96) | 67980.89(57541.82-80038.12) | 380.01(321.65-447.41) | 1.86(1.64-2.08) |
| 95+ years | 2227.58(1715.78-2874.10) | 218.80(168.53-282.30) | 18809.58(15130.42-23438.59) | 345.11(277.61-430.04) | 1.65(1.55-1.76) |

ILD&PS, Interstitial lung disease and pulmonary sarcoidosis; ASPR, Age-standardized prevalence rate; EAPC, Estimated annual percentage change; UI, Uncertainty interval; CI, Confidence interval.

**Table S11. Number and age-standardized incidence of ILD&PS, 1990 vs. 2021 (Global distribution by sex and age).**

| **Items** | **Number in 1990  (95% UI)** | **ASIR in 1990  (per 100 000, 95% UI)** | **Number in 2021  (95% UI)** | **ASIR in 2021  (per 100 000, 95% UI)** | **EAPC of ASIR  (95% CI)** |
| --- | --- | --- | --- | --- | --- |
| **Sex** |  |  |  |  |  |
| Male | 86263.97(74477.21-98468.22) | 4.48(3.89-5.05) | 214681.18(190533.20-238498.19) | 5.36(4.80-5.95) | 0.71(0.6-0.81) |
| Female | 71177.21(61733.29-81297.25) | 3.23(2.80-3.68) | 175585.93(155725.25-195606.72) | 3.89(3.46-4.31) | 0.71(0.63-0.8) |
| **Age-group** |  |  |  |  |  |
| 0-14 years | 0.00(0.00-0.00) | 0.00(0.00-0.00) | 0.00(0.00-0.00) | 0.00(0.00-0.00) | 0.00(0.00-0.00) |
| 15-19 years | 1500.46(528.49-3064.13) | 0.29(0.10-0.59) | 1767.20(681.03-3491.53) | 0.28(0.11-0.56) | 0.13(0.04-0.22) |
| 20-24 years | 4336.39(2476.56-6815.73) | 0.88(0.50-1.39) | 4733.90(2869.27-7257.73) | 0.79(0.48-1.22) | -0.38(-0.43--0.33) |
| 25-29 years | 6209.93(3290.85-9678.10) | 1.40(0.74-2.19) | 6808.14(3711.74-10563.59) | 1.16(0.63-1.80) | -0.67(-0.72--0.62) |
| 30-34 years | 7133.03(3747.31-11239.04) | 1.85(0.97-2.92) | 9078.74(4866.28-13988.22) | 1.50(0.81-2.31) | -0.62(-0.67--0.58) |
| 35-39 years | 8564.47(4891.60-13743.75) | 2.43(1.39-3.90) | 11931.70(6972.50-18689.79) | 2.13(1.24-3.33) | -0.42(-0.44--0.4) |
| 40-44 years | 10080.48(5635.35-15656.33) | 3.52(1.97-5.47) | 15917.83(9240.99-24056.96) | 3.18(1.85-4.81) | -0.29(-0.32--0.25) |
| 45-49 years | 12231.05(7313.28-18915.87) | 5.27(3.15-8.15) | 23443.51(14332.24-34940.29) | 4.95(3.03-7.38) | -0.17(-0.21--0.13) |
| 50-54 years | 15079.56(8362.77-22212.45) | 7.09(3.93-10.45) | 31699.14(18533.25-45293.35) | 7.12(4.17-10.18) | -0.01(-0.08-0.05) |
| 55-59 years | 17969.39(10981.89-26056.50) | 9.70(5.93-14.07) | 40476.87(25780.57-57982.32) | 10.23(6.51-14.65) | 0.25(0.16-0.34) |
| 60-64 years | 20156.13(12390.74-29666.49) | 12.55(7.71-18.47) | 48087.03(30735.90-68066.51) | 15.02(9.60-21.27) | 0.65(0.5-0.8) |
| 65-69 years | 17908.58(11596.69-26767.91) | 14.49(9.38-21.66) | 52601.22(34568.21-74257.41) | 19.07(12.53-26.92) | 1.17(0.98-1.35) |
| 70-74 years | 14063.37(8520.72-20085.01) | 16.61(10.06-23.72) | 52707.37(33629.15-72822.83) | 25.61(16.34-35.38) | 1.17(0.98-1.35) |
| 75-79 years | 10336.43(6766.97-14822.63) | 16.79(10.99-24.08) | 37702.18(25772.23-51768.69) | 28.59(19.54-39.25) | 1.75(1.62-1.88) |
| 80-84 years | 6654.17(4471.69-9757.63) | 18.81(12.64-27.58) | 26369.85(18236.18-38194.71) | 30.11(20.82-43.61) | 1.81(1.75-1.87) |
| 85-89 years | 3592.45(2732.78-4686.57) | 23.77(18.08-31.01) | 16721.71(13177.55-21243.05) | 36.57(28.82-46.46) | 1.6(1.38-1.81) |
| 90-94 years | 1263.80(914.86-1752.38) | 29.49(21.35-40.89) | 7606.06(5645.30-10266.62) | 42.52(31.56-57.39) | 1.46(1.24-1.68) |
| 95+ years | 361.50(230.55-521.30) | 35.51(22.64-51.20) | 2614.64(1745.20-3738.11) | 47.97(32.02-68.59) | 1.28(1.1-1.46) |

ILD&PS, Interstitial lung disease and pulmonary sarcoidosis; ASIR, Age-standardized incidence rate; EAPC, Estimated annual percentage change; UI, Uncertainty interval; CI, Confidence interval.

**Table S12. Number and age-standardized DALYs of ILD&PS, 1990 vs. 2021 (Global distribution by sex and age).**

| **Items** | **Number in 1990  (95% UI)** | **ASDR in 1990  (per 100 000, 95% UI)** | **Number in 2021  (95% UI)** | **ASDR in 2021  (per 100 000, 95% UI)** | **EAPC of ASDR  (95% CI)** |
| --- | --- | --- | --- | --- | --- |
| **Sex** |  |  |  |  |  |
| Male | 853132.96(666666.89-1066356.17) | 46.48(36.62-57.59) | 2237269.37(1839499.94-2555199.73) | 57.79(47.50-65.77) | 0.85(0.76-0.94) |
| Female | 647895.47(500164.80-883303.45) | 29.79(23.23-40.19) | 1804881.12(1465706.81-2216375.57) | 39.49(31.95-48.62) | 1.07(0.96-1.17) |
| **Age-group** |  |  |  |  |  |
| 0-14 years | 42350.69(22243.53-73782.34) | 2.44(1.28-4.24) | 24876.66(15521.28-34143.63) | 1.24(0.77-1.70) | -1.94(-2.12--1.76) |
| 15-19 years | 13415.48(9852.31-18665.44) | 2.58(1.90-3.59) | 18161.67(14446.85-21485.55) | 2.91(2.32-3.44) | 0.5(0.36-0.64) |
| 20-24 years | 18614.48(13861.64-24934.43) | 3.78(2.82-5.07) | 28714.24(22808.75-34551.82) | 4.81(3.82-5.79) | 0.71(0.62-0.81) |
| 25-29 years | 27509.27(21170.11-35325.99) | 6.22(4.78-7.98) | 41574.76(34413.11-49214.92) | 7.07(5.85-8.37) | 0.47(0.35-0.58) |
| 30-34 years | 38026.23(28893.99-48238.04) | 9.87(7.50-12.52) | 62193.45(48212.16-74477.72) | 10.29(7.98-12.32) | 0.19(0.11-0.27) |
| 35-39 years | 51874.27(39696.69-67234.11) | 14.73(11.27-19.09) | 86301.49(70633.03-102999.74) | 15.39(12.59-18.36) | 0.13(0.07-0.19) |
| 40-44 years | 62224.86(47792.42-82282.61) | 21.72(16.68-28.72) | 114509.01(92099.62-137452.23) | 22.89(18.41-27.48) | 0.02(-0.06-0.1) |
| 45-49 years | 79659.01(60791.39-101219.41) | 34.31(26.18-43.59) | 160193.51(129614.29-193353.85) | 33.83(27.37-40.83) | -0.08(-0.15--0.01) |
| 50-54 years | 111032.85(84524.50-145624.15) | 52.23(39.76-68.51) | 221371.97(177304.60-267163.00) | 49.76(39.85-60.05) | -0.19(-0.27--0.11) |
| 55-59 years | 151442.43(116196.70-195311.16) | 81.77(62.74-105.46) | 330651.60(266121.54-388739.69) | 83.56(67.25-98.23) | 0.11(0.02-0.21) |
| 60-64 years | 195518.40(152620.46-252684.16) | 121.74(95.03-157.33) | 419878.38(344893.88-501939.70) | 131.19(107.76-156.83) | 0.21(0.08-0.33) |
| 65-69 years | 206531.22(165763.39-253520.53) | 167.08(134.10-205.10) | 522370.04(434191.57-604072.93) | 189.37(157.41-218.99) | 0.62(0.45-0.78) |
| 70-74 years | 184848.23(149900.65-230258.10) | 218.34(177.06-271.98) | 610775.30(514813.62-715439.22) | 296.72(250.10-347.57) | 1.2(1.09-1.31) |
| 75-79 years | 159645.63(136680.21-191155.79) | 259.35(222.04-310.54) | 538844.88(470898.59-607654.97) | 408.57(357.05-460.75) | 1.56(1.44-1.68) |
| 80-84 years | 97168.23(83255.56-114807.16) | 274.67(235.35-324.53) | 422882.23(360008.95-483633.26) | 482.84(411.05-552.20) | 2.14(1.95-2.34) |
| 85-89 years | 43762.19(38232.95-50032.29) | 289.60(253.01-331.10) | 265665.82(222273.47-298319.83) | 581.05(486.14-652.47) | 2.7(2.44-2.96) |
| 90-94 years | 13796.08(11511.82-15855.89) | 321.95(268.64-370.02) | 127031.41(100800.39-144301.05) | 710.09(563.47-806.63) | 3.05(2.79-3.32) |
| 95+ years | 3608.87(2838.20-4212.36) | 354.48(278.78-413.75) | 46154.07(33944.37-53418.97) | 846.82(622.80-980.11) | 3.15(2.94-3.36) |

ILD&PS, Interstitial lung disease and pulmonary sarcoidosis; DALYs, Disability-adjusted life years; ASDR, Age-standardized DALYs rate; EAPC, Estimated annual percentage change; UI, Uncertainty interval; CI, Confidence interval.

**Table S13. Number and age-standardized mortality of ILD&PS, 1990 vs. 2021 (Global distribution by sex and age).**

| **Items** | **Number in 1990  (95% UI)** | **ASMR in 1990  (per 100 000, 95% UI)** | **Number in 2021  (95% UI)** | **ASMR in 2021  (per 100 000, 95% UI)** | **EAPC of ASMR  (95% CI)** |
| --- | --- | --- | --- | --- | --- |
| **Sex** |  |  |  |  |  |
| Male | 31182.61(24196.09-39011.60) | 2.01(1.61-2.48) | 103056.70(84156.40-115833.40) | 2.90(2.40-3.24) | 1.41(1.28-1.53) |
| Female | 23784.63(18180.45-32746.57) | 1.17(0.90-1.60) | 85165.67(68720.00-105539.29) | 1.83(1.48-2.27) | 1.7(1.54-1.86) |
| **Age-group** |  |  |  |  |  |
| 0-14 years | 502.87(266.85-872.12) | 0.03(0.02-0.05) | 300.45(189.49-409.59) | 0.01(0.01-0.02) | -1.89(-2.07--1.71) |
| 15-19 years | 180.89(132.59-252.86) | 0.03(0.03-0.05) | 245.69(195.26-292.58) | 0.04(0.03-0.05) | 0.51(0.36-0.65) |
| 20-24 years | 247.69(179.61-326.99) | 0.05(0.04-0.07) | 392.35(305.51-472.52) | 0.07(0.05-0.08) | 0.79(0.68-0.89) |
| 25-29 years | 367.72(275.74-500.92) | 0.08(0.06-0.11) | 580.60(469.71-689.92) | 0.10(0.08-0.12) | 0.61(0.48-0.75) |
| 30-34 years | 533.80(392.14-688.22) | 0.14(0.10-0.18) | 919.11(699.47-1094.61) | 0.15(0.12-0.18) | 0.36(0.27-0.45) |
| 35-39 years | 791.25(590.41-1052.01) | 0.22(0.17-0.30) | 1381.01(1098.62-1680.77) | 0.25(0.20-0.30) | 0.28(0.21-0.35) |
| 40-44 years | 1040.77(767.45-1400.37) | 0.36(0.27-0.49) | 2013.30(1561.09-2543.95) | 0.40(0.31-0.51) | 0.16(0.07-0.24) |
| 45-49 years | 1520.12(1115.19-1981.91) | 0.65(0.48-0.85) | 3138.32(2461.83-3855.19) | 0.66(0.52-0.81) | 0.02(-0.06-0.09) |
| 50-54 years | 2429.09(1781.65-3326.94) | 1.14(0.84-1.57) | 4883.72(3776.27-6017.98) | 1.10(0.85-1.35) | -0.16(-0.25--0.07) |
| 55-59 years | 3869.47(2913.38-5128.93) | 2.09(1.57-2.77) | 8544.45(6736.81-10399.99) | 2.16(1.70-2.63) | 0.14(0.04-0.25) |
| 60-64 years | 5883.51(4418.87-7865.57) | 3.66(2.75-4.90) | 12726.43(10023.38-15308.08) | 3.98(3.13-4.78) | 0.21(0.08-0.33) |
| 65-69 years | 7448.28(5748.64-9365.89) | 6.03(4.65-7.58) | 18982.84(15254.04-21873.08) | 6.88(5.53-7.93) | 0.63(0.46-0.81) |
| 70-74 years | 8205.13(6492.32-10590.12) | 9.69(7.67-12.51) | 27436.11(22263.24-32855.84) | 13.33(10.82-15.96) | 1.24(1.12-1.37) |
| 75-79 years | 8978.89(7524.17-10932.15) | 14.59(12.22-17.76) | 30777.94(26415.78-34918.06) | 23.34(20.03-26.48) | 1.62(1.5-1.74) |
| 80-84 years | 7018.98(5872.63-8454.76) | 19.84(16.60-23.90) | 31227.21(26079.54-36046.41) | 35.65(29.78-41.16) | 2.24(2.04-2.44) |
| 85-89 years | 4040.10(3465.98-4673.05) | 26.74(22.94-30.92) | 25124.42(20406.10-28319.72) | 54.95(44.63-61.94) | 2.8(2.54-3.06) |
| 90-94 years | 1489.94(1215.92-1721.22) | 34.77(28.37-40.17) | 14012.65(10960.63-15981.50) | 78.33(61.27-89.34) | 3.13(2.87-3.4) |
| 95+ years | 418.73(325.19-494.08) | 41.13(31.94-48.53) | 5535.79(3988.01-6452.37) | 101.57(73.17-118.39) | 3.28(3.06-3.5) |

ILD&PS, Interstitial lung disease and pulmonary sarcoidosis; ASMR, Age-standardized mortality rate; EAPC, Estimated annual percentage change; UI, Uncertainty interval; CI, Confidence interval.
