# Supplementary material for: Global, regional and national burden of interstitial lung disease and pulmonary sarcoidosis, 1990–2021 and projection to 2040
Source: Front Med (Lausanne). 2025 Oct 27;12:1650997. doi: 10.3389/fmed.2025.1650997 (PMC12599143; doi:10.3389/fmed.2025.1650997)
Supplement: Supplementary file 2 [file Supplementary_file_1.docx]

**
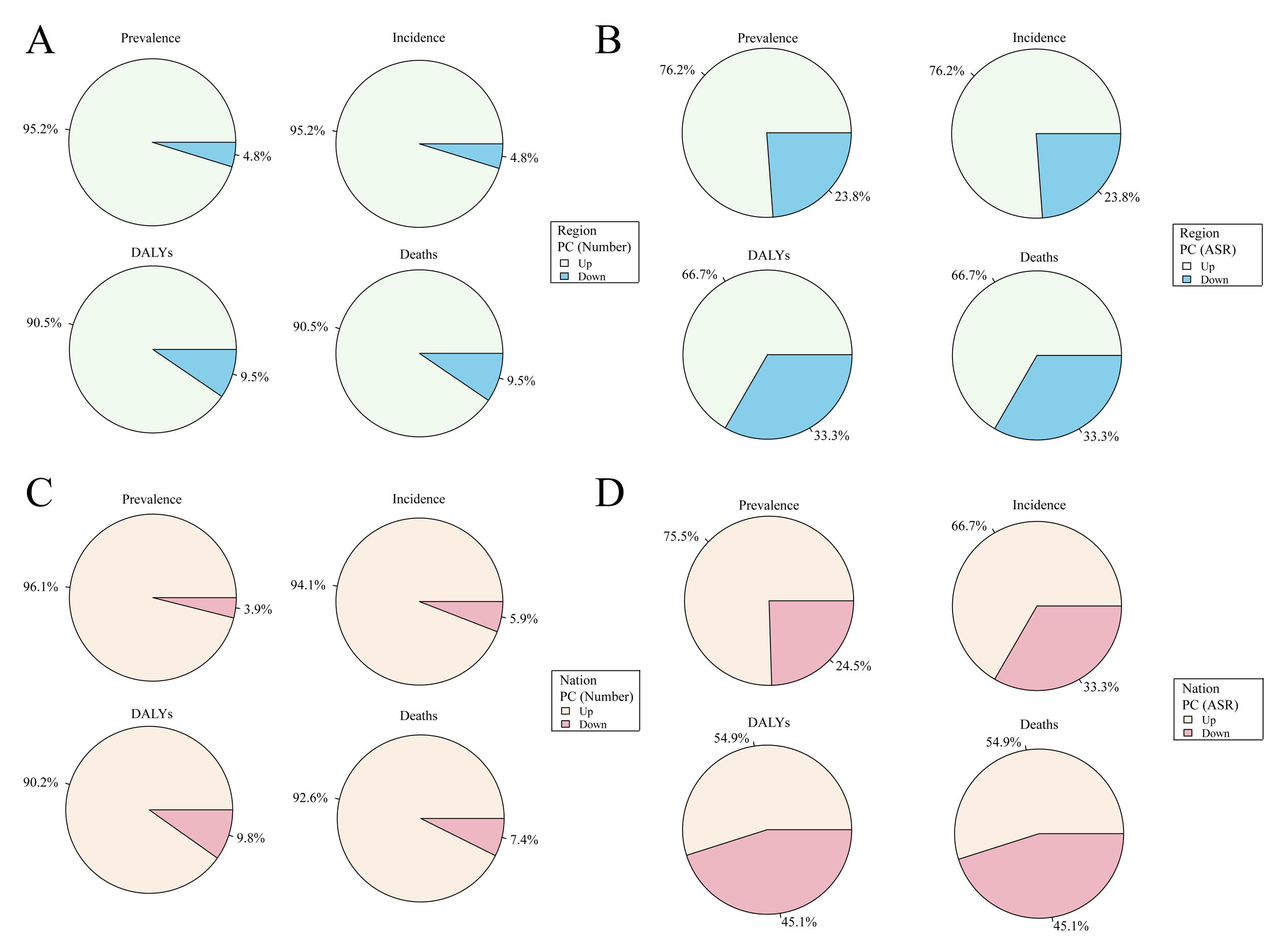
**

**Fig S1. Trends in the PC of cases and ASR for ILD&PS burden in 21 regions and 204 countries, 1990 to 2021. (A-B)** Trends of PC in the number and ASR of ILD&PS burden across 21 regions; **(C-D)** Trends of PC in the number and ASR of ILD&PS burden across 204 countries.

PC, Percentage change; ASR, Age-standardized rate; ILD&PS, Interstitial lung disease and pulmonary sarcoidosis; DALYs, Disability adjusted life years.


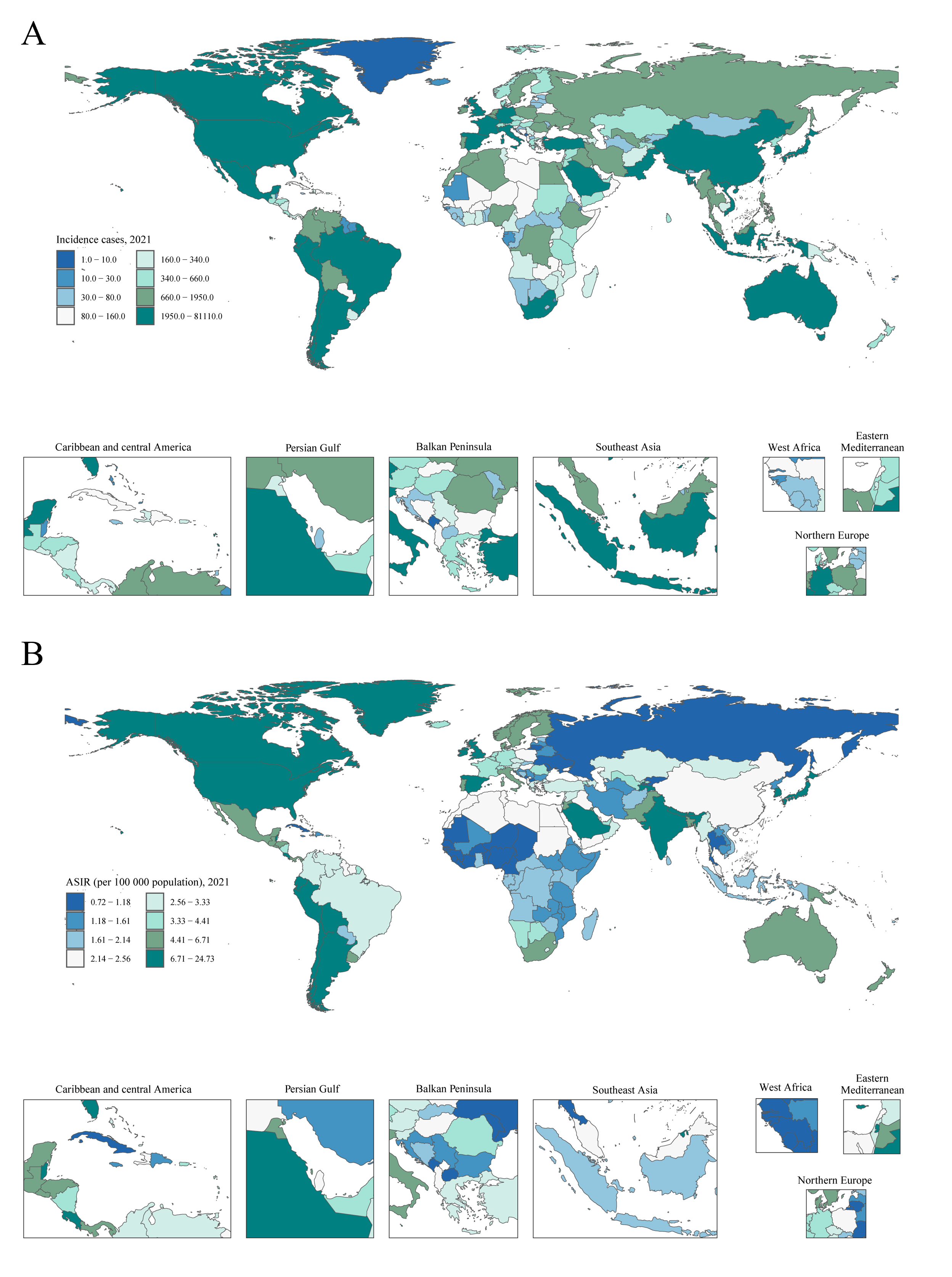


**Fig S2. Incidence and age-standardized rates of ILD&PS in 204 countries and territories. (A)** Incidence cases in 2021; **(B)** ASIR per 100,000 population in 2021.

ILD&PS, Interstitial lung disease and pulmonary sarcoidosis; ASIR, Age-standardized incidence rate.

**
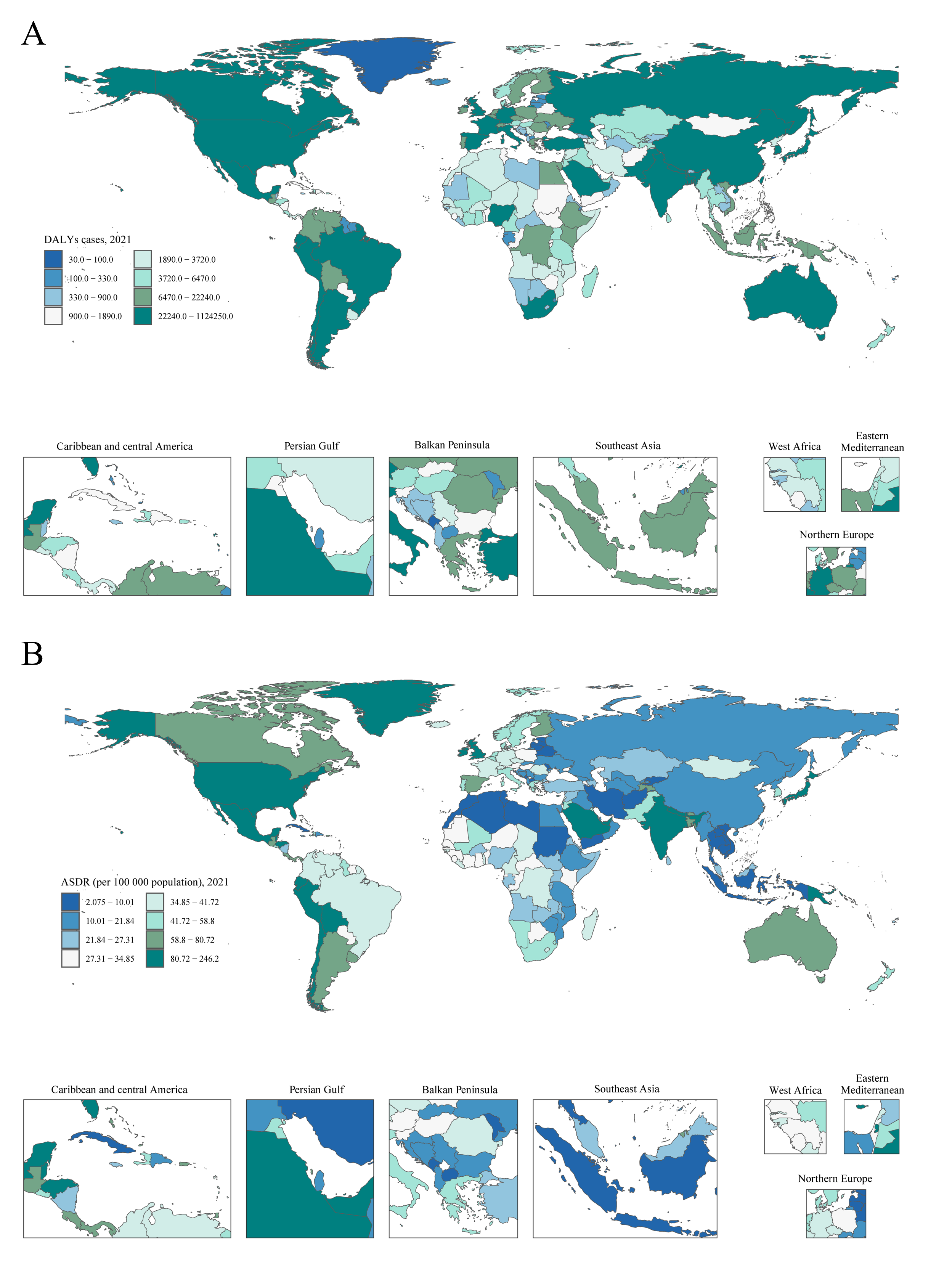
**

**Fig S3. DLAYs and age-standardized rates for ILD&PS in 204 countries and territories. (A)** DLAYs cases in 2021; **(B)** ASDR per 100,000 population in 2021.

ILD&PS, Interstitial lung disease and pulmonary sarcoidosis; DALYs, Disability adjusted life years; ASDR, Age-standardized DALYs rate.

**
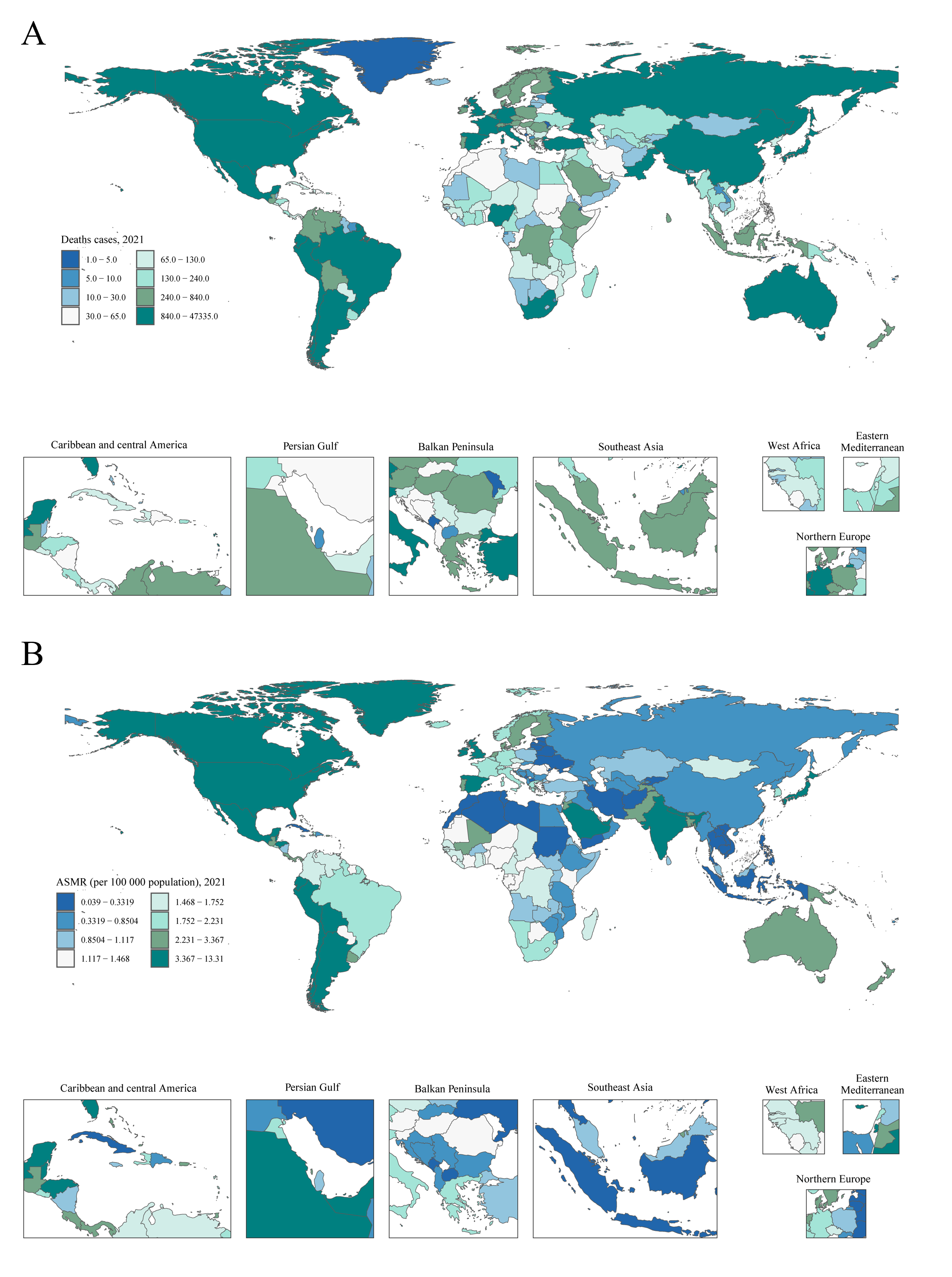
**

**Fig S4. Deaths and age-standardized rates for ILD&PS in 204 countries and territories. (A)** Deaths cases in 2021; **(B)** ASMR per 100,000 population in 2021.

ILD&PS, Interstitial lung disease and pulmonary sarcoidosis; ASMR, Age-standardized mortality rate.

**
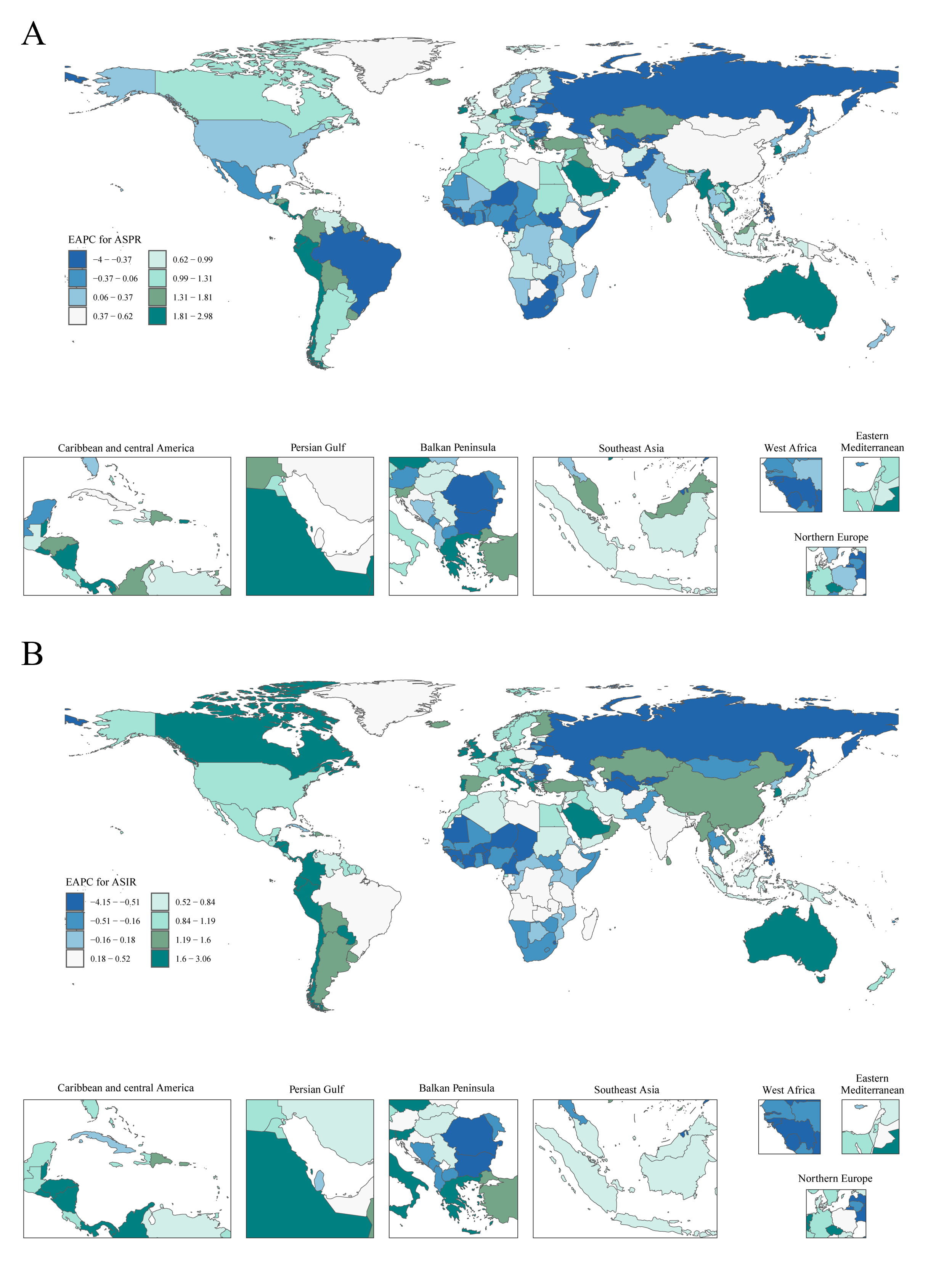
**

**Fig S5. EAPC of ASPR and ASIR for ILD&PS in 204 countries and territories, 1990-2021. (A)** ASPR in 204 countries and territories. **(B)** ASIR in 204 countries and territories.

ILD&PS, Interstitial lung disease and pulmonary sarcoidosis; EAPC, Estimated annual percentage changes; ASPR, Age-standardized prevalence rate; ASIR, Age-standardized incidence rate.

**
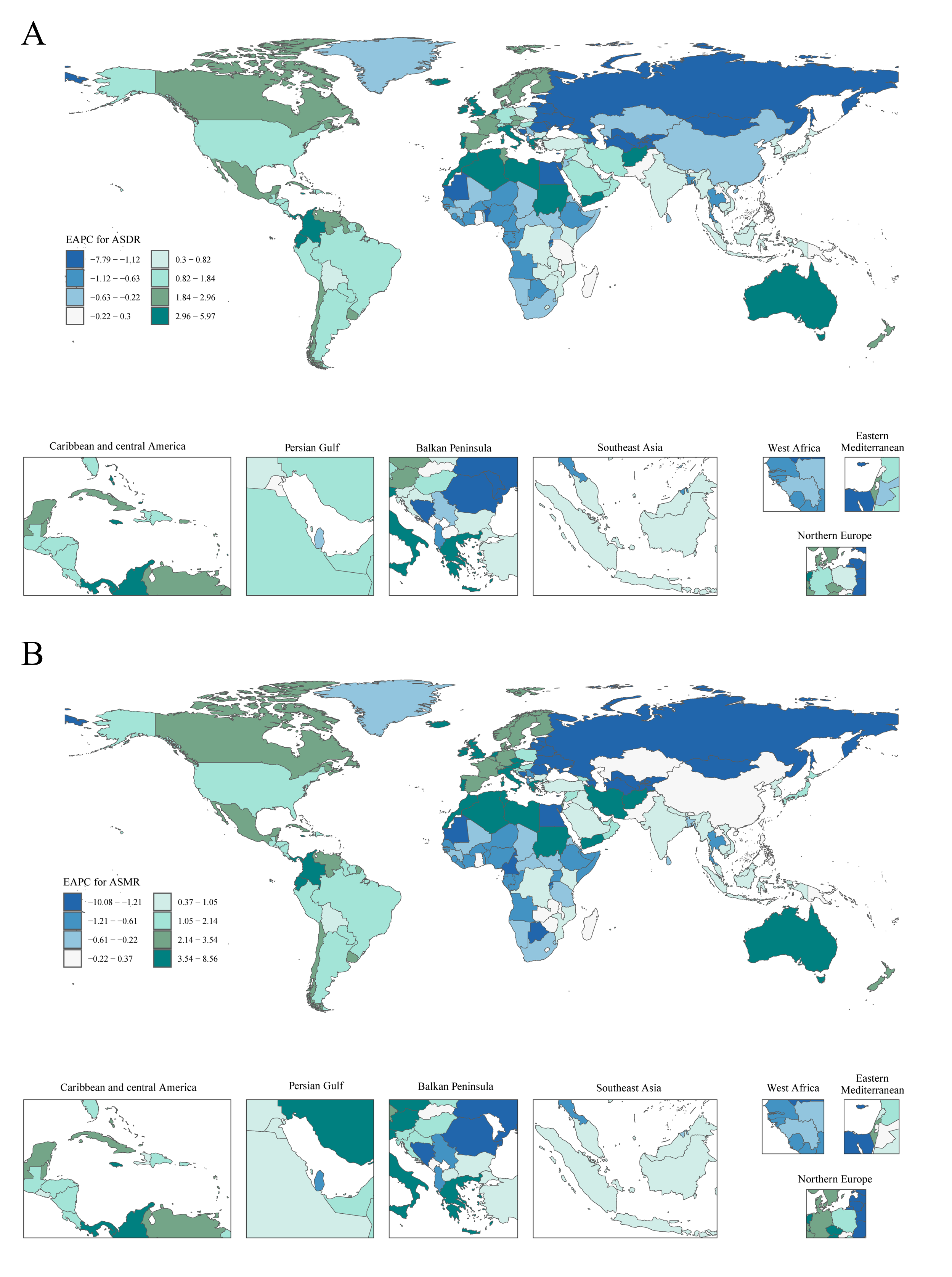
**

**Fig S6. EAPC of ASDR and ASMR for ILD&PS in 204 countries and territories, 1990-2021. (A)** ASDR in 204 countries and territories. **(B)** ASMR in 204 countries and territories.

ILD&PS, Interstitial lung disease and pulmonary sarcoidosis; EAPC, Estimated annual percentage changes; ASDR, Age-standardized DALYs rate; ASMR, Age-standardized mortality rate.


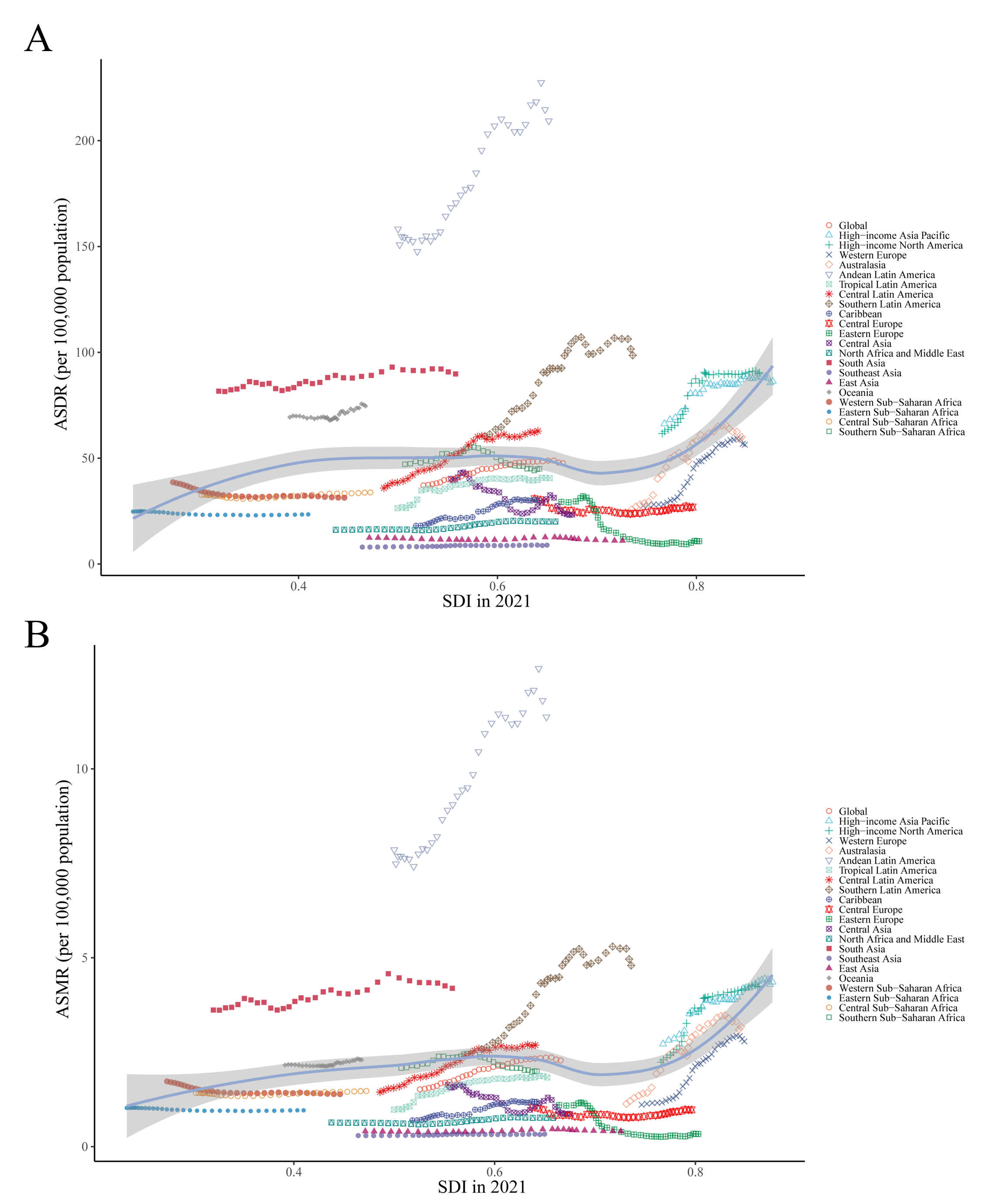


**Fig S7. ASDR and ASMR for ILD&PS by SDI in 2021, globally and in 21 regions, 1990 to 2021. (A)** ASDR across 21 regions according to SDI in 2021; **(B)** ASMR across 21 regions according to SDI in 2021.

ASDR, Age-standardized DALYs rate; ASMR, Age-standardized mortality rate; DALYs, Disability adjusted life years; SDI, socio-demographic index.

**
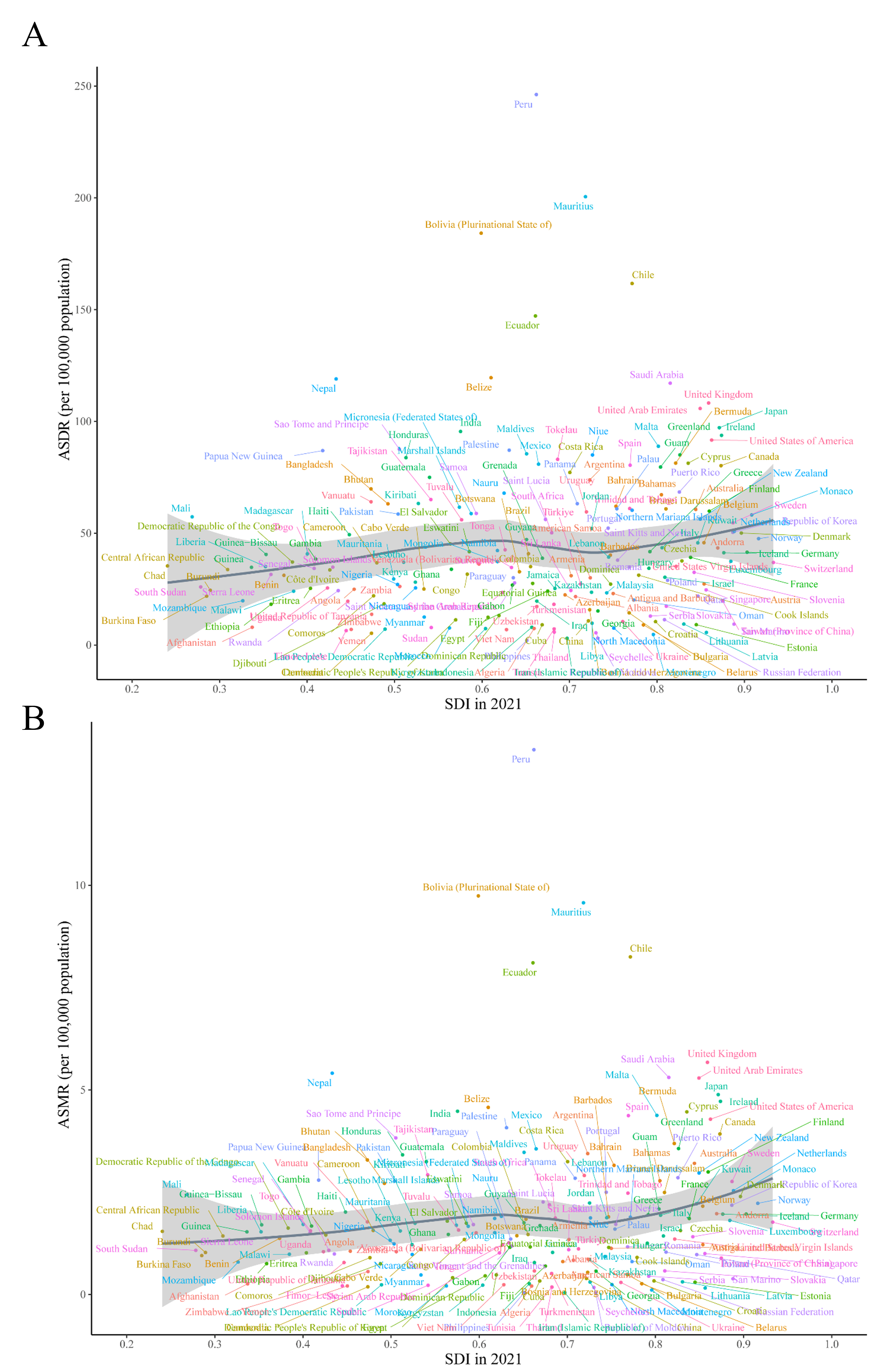
**

**Fig S8. ASDR and ASMR of ILD&PS by the 2021 SDI in 204 countries and territories, 1990 to 2021.** **(A)** ASDR in 204 countries and territories; **(B)** ASMR in 204 countries and territories.

ILD&PS, Interstitial lung disease and pulmonary sarcoidosis; ASDR, Age-standardized DALYs rate; ASMR, Age-standardized mortality rate; DALYs, Disability adjusted life years; SDI, Socio-demographic index.

**
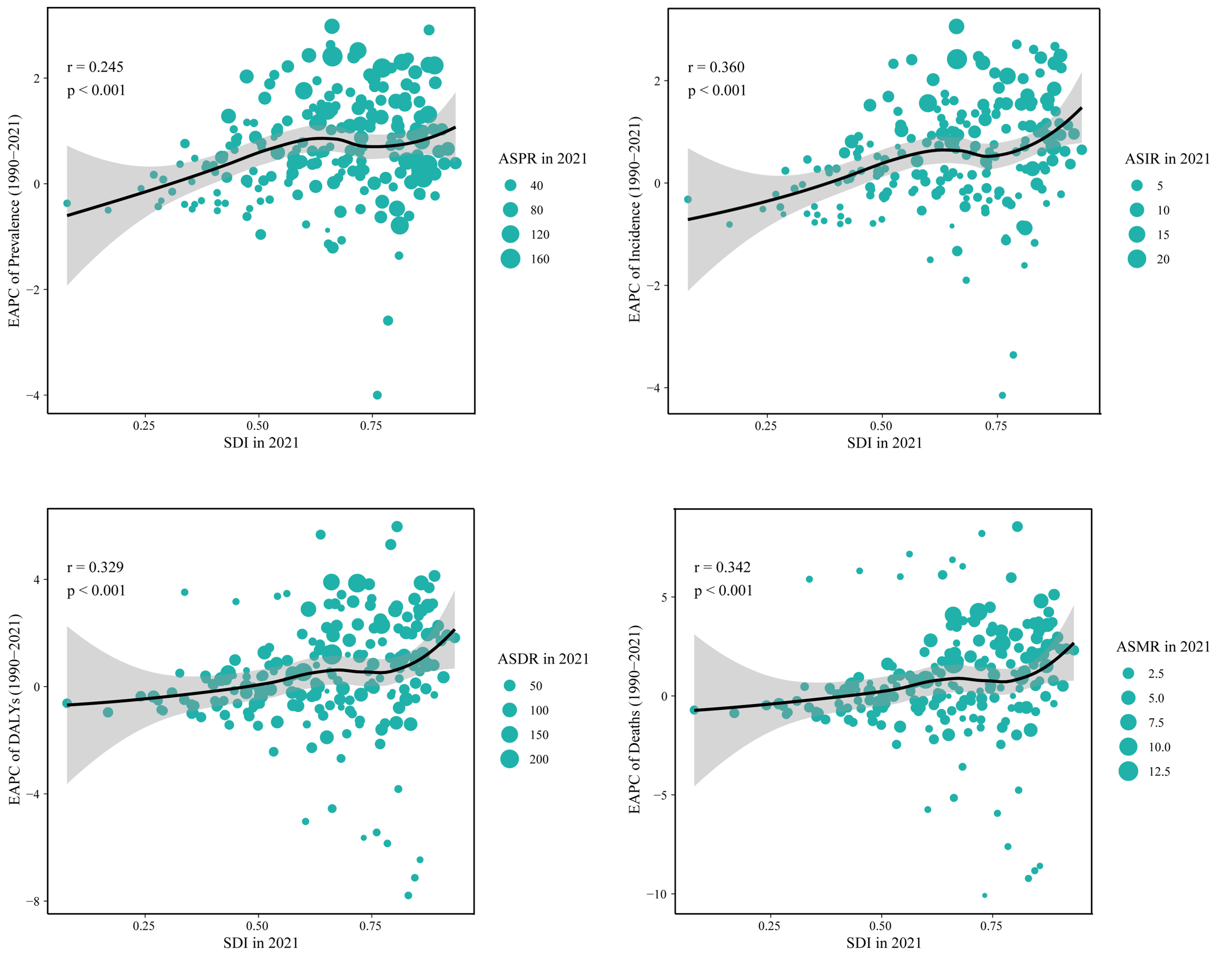
**

**Fig S9. Correlation between the SDI and EAPC of ILD&PS burden, by ASR for 204 countries in 2021.**

SDI, socio-demographic index; EAPC, Estimated annual percentage changes; ILD&PS, Interstitial lung disease and pulmonary sarcoidosis; ASR, Age-standardized rate; ASPR, Age-standardized prevalence rate; ASIR, Age-standardized incidence rate; ASDR, Age-standardized DALYs rate; DALYs, Disability adjusted life years; ASMR, Age-standardized mortality rate.

**
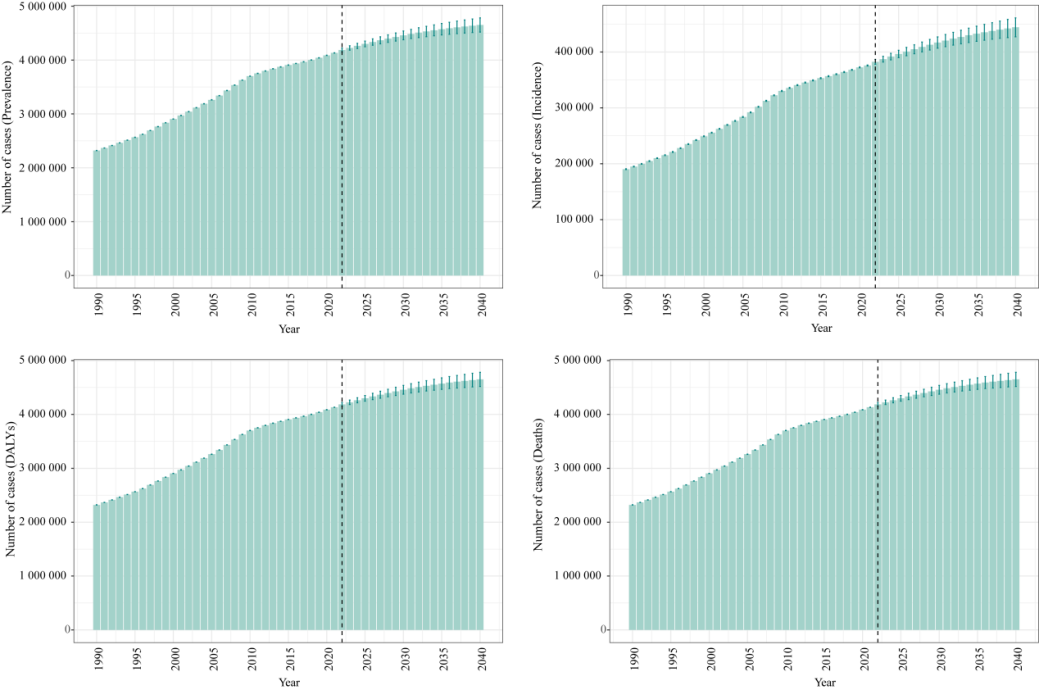
**

**Fig S10. Global trends in the projected quantitative burden of ILD&PS.**

ILD&PS, Interstitial lung disease and pulmonary sarcoidosis
